# Demographic variation in self-rated physical health across 22 countries: findings from the Global Flourishing Study

**DOI:** 10.1186/s44263-025-00141-1

**Published:** 2025-04-30

**Authors:** Matt Bradshaw, Blake Victor Kent, Jeff Levin, Jennifer Susan Wortham, Noémie Le Pertel, Tyler J. VanderWeele, Byron R. Johnson

**Affiliations:** 1 Institute for Studies of Religion, Baylor University, One Bear Place #97326, Waco, TX 76798-7326 USA; 2 Department of Sociology and Anthropology, Westmont College, Santa Barbara, CA USA; 3 Institute for Studies of Religion and Medical Humanities Program, Baylor University, Waco, TX USA; 4 Department of Psychiatry and Behavioral Sciences, Duke University School of Medicine, Durham, NC USA; 5 Human Flourishing Program, Institute for Quantitative Social Science, Harvard University, Cambridge, MA USA; 6 Department of Biostatistics and Department of Epidemiology, Harvard T.H. Chan School of Public Health, Boston, MA USA; 7 Institute for Studies of Religion, Baylor University, Waco, TX USA; 8 School of Public Policy, Pepperdine University, Malibu, CA USA

**Keywords:** Well-being, Culture, Cross-national, Age, Gender, Marital status, Socioeconomic status, Religion, Public health

## Abstract

**Background:**

Relatively few studies have examined how self-rated physical health (SRH) varies across: (a) countries around the world and (b) demographic characteristics in diverse nations and cultures.

**Methods:**

The current study addresses these issues by providing a cross-national random effects meta-analysis of SRH using data from the Global Flourishing Study (GFS), an international survey of 202,898 individuals from 22 geographically, economically, and culturally diverse countries collected in 2022–2023.

**Results:**

On a scale of 0–10 (0 = poor and 10 = excellent), the mean SRH ranged from 5.97 in Japan to 8.29 in Indonesia. Three of the four largest SRH means were found in developing, non-Western countries (Indonesia, Nigeria, and Kenya), while the five lowest were in economically developed nations (Germany, Australia, Sweden, the UK, and Japan). Countries also differed in the degree of variation around the mean. SRH was more evenly dispersed in nations like Israel, Poland, and the USA and more unequally distributed in places like Egypt, Tanzania, and India. SRH also varied across demographic characteristics. Results from a random effects meta-analysis of all 22 countries showed that SRH varied across age, gender, marital status, employment, education, religious service attendance, and immigration status in at least some countries. In general, SRH tended to be higher among: (a) younger individuals; (b) males; (c) those who were single/never married, married, or had domestic partnerships (compared with other groups such as widowed, divorced, or separated); (d) employed individuals and students; (e) people with more years of education; and (f) those who attended religious services. There was considerable heterogeneity across countries in the associations between demographic characteristics and SRH, however, suggesting that country-level contexts are important. Results were similar when weighted based on the population size in each country.

**Conclusions:**

While being mindful of challenges due to varying cultural contexts and possible interpretations and translations of key survey questions, findings suggest substantial variation in SRH across countries and meaningful demographic characteristics. This study lays the foundation for future longitudinal GFS studies on the causes and correlates of SRH in a global context.

**Supplementary Information:**

The online version contains supplementary material available at 10.1186/s44263-025-00141-1.

## Background

Research on human flourishing has increased rapidly in recent years [1–6]. There are still weaknesses in this literature, however, including relatively few longitudinal studies and a limited number of publications in countries and cultures outside of the USA and Europe [7–9]. The Global Flourishing Study (GFS) seeks to address these shortcomings by providing an intended five waves of panel data on many different aspects of human flourishing (e.g., mental and physical health, happiness and life satisfaction, meaning and purpose, character and virtue, close social relationships, and financial stability) in 22 geographically, economically, and culturally diverse countries around the world on a sample of over 200,000 individuals [7, 10]. The first wave of data collection is complete, and the second is underway.

The current study focuses on one specific aspect of human flourishing: self-rated physical health (SRH). This widely used indicator of health status has been shown to predict morbidity and mortality [11, 12], mental health [13, 14], medical conditions and functional limitations [15, 16], and healthcare utilization [17, 18]. It is routinely employed by economists, epidemiologists, medical and health professionals, psychologists, public health scholars, and sociologists interested in studying disease risk factors in large and diverse populations [11, 19–21]. SRH has also been examined in different countries and cultures [15, 22, 23].

SRH appears to vary around the world [15, 24–29], with nations such as Australia, Sweden, and the USA reporting better health compared with others like Germany, Israel, and Japan [30]. The vast majority of research has been conducted in developed and Western countries, however, with most focusing on Europe and the USA [22, 31–37]. Relatively few studies have examined nations in other parts of the world [15, 23, 25, 27, 29, 38]. Among the countries and territories included in the GFS, numerous papers have been published using data from Australia [15, 39], Germany [36, 40], Hong Kong/China [28, 41], India [42, 43], Israel [44, 45], Japan [15, 46], Poland [47, 48], South Africa [29, 49, 50], Spain [51, 52], Sweden [53, 54], the UK [55, 56], and the USA [29, 57, 58]. In contrast, we know far less about Argentina [29, 59], Brazil [29, 60], Egypt [61], Indonesia [62], Mexico [29, 63], Nigeria [64], the Philippines [65], and Türkiye [66] and virtually nothing about Kenya and Tanzania. Further, much of this work is based on individual countries, and cross-national comparisons are difficult due to varying study designs, measures, and samples. Given our limited knowledge of many nations, the first objective here is to examine levels of SRH across GFS countries.

In addition to documenting patterns of SRH around the world, it is also important to begin understanding the causes of this variation. Existing cross-national research suggests that SRH is shaped in unique ways by a variety of factors across countries, cultures, and contexts [25, 29, 32, 33, 35]. These include differences in (a) actual morbidity, (b) knowledge about health that may shape self-assessments, (c) reference groups for health comparisons based on population characteristics (e.g., age structure, longevity, illness), (d) the meaning assigned to health in different cultures and contexts, (e) survey question interpretation and understanding, (f) willingness to report positive or negative aspects of one’s life or talk about sensitive issues, and (g) other variables that are correlated with SRH such as demographic factors, socioeconomic status (SES), and various social and cultural influences [15, 20, 22, 23, 27, 30, 67]. The present study does not investigate these types of associations in a causal fashion; instead, the second objective is to lay the initial descriptive groundwork by documenting variations in SRH across sociodemographic characteristics in the entire GFS sample and for each individual country.

Fully understanding cross-national trends is the ultimate long-term goal of the GFS, and many diverse lines of inquiry will build on this baseline study in the coming years. Future papers will employ multivariate models and longitudinal panel data to examine the effects of known correlates including (a) demographic characteristics such as age, gender, and race/ethnicity [21, 32, 57, 58]; (b) SES, neighborhood conditions, and social capital [29, 68–72]; (c) social relationships and support, as well as a sense of personal control [73, 74]; (d) cultural influences such as religious practices and beliefs [75, 76]; and (e) macro-level factors including Gross Domestic Product (GDP), income inequality, fertility and mortality, levels of government in involvement in population health and health care, individualistic/collectivistic cultures, and social capital [33, 77–79]. These are important because they shape exposure to adverse conditions and events that could undermine health, as well as resources that may promote well-being [80–82]. With the second wave of data arriving in 2025, the GFS will offer a unique opportunity to examine key predictors of SRH in a large and globally diverse sample that includes developing and non-Western nations.

Prior to these types of causal analyses, however, it is necessary to document basic descriptive patterns of SRH across the 22 GFS countries using the first wave of data. Two research questions, which were preregistered with the Center for Open Science (COS), are addressed here. First, how are mean levels of SRH ordered across participating countries? Second, how does SRH vary across demographic categories in these countries?

## Methods

### Data

Data come from the first wave of the GFS, which examines the distribution and determinants of well-being across a sample of 202,898 participants from 22 diverse countries. Wave 1 of the GFS collected nationally representative data from the following countries and territories: Argentina, Australia, Brazil, Egypt, Germany, Hong Kong (Special Administrative Region of China, with mainland China also included from 2024 onwards), India, Indonesia, Israel, Japan, Kenya, Mexico, Nigeria, the Philippines, Poland, South Africa, Spain, Sweden, Tanzania, Türkiye, the UK, and the USA. These countries were chosen to (a) maximize coverage of the world's population; (b) ensure geographic, cultural, and religious diversity; and (c) prioritize feasibility and existing data collection infrastructure. Gallup, Inc. conducted the data collection primarily in 2023, although some regions began in 2022; timing varied by country, and more information can be found in the Gallup documentation files [83]. The precise sampling design to ensure nationally representative samples varied by country and further details are available elsewhere [83]. The translation process followed the TRAPD model (translation, review, adjudication, pretesting, and documentation) for cross-cultural survey research (ccsg.isr.umich.edu/chapters/translation/overview). Further details about survey development and methodology are documented in the GFS Questionnaire Development Report [84], Methodology [83], Codebook (https://osf.io/cg76b), and Translations documents [85]. The data are publicly available through COS (https://www.cos.io/gfs). Additional information is provided elsewhere [10, 86, 87].

### Measures

#### Dependent variable

SRH was measured with a single indicator [88]: “In general, how would you rate your PHYSICAL health?” Respondents were instructed to rate their health on a 0–10 scale, where 0 = poor and 10 = excellent. This measure was treated as continuous.

#### Demographic variables

A continuous age variable was collapsed into the following categories: 18–24, 25–29, 30–39, 40–49, 50–59, 60–69, 70–79, and 80 or older. Gender was assessed as male, female, or other. Marital status was assessed as single/never married, married, separated, divorced, widowed, and domestic partner. Employment was assessed as employed, self-employed, retired, student, homemaker, unemployed and searching, and other. Education was assessed as up to 8 years, 9–15 years, and 16 + years. Service attendance was assessed as more than once/week, once/week, one to three times/month, a few times/year, or never. Immigration status was dichotomously assessed with “Were you born in this country, or not?” Religious tradition was measured with categories of Christianity, Islam, Hinduism, Buddhism, Judaism, Sikhism, Baha’i, Jainism, Shinto, Taoism, Confucianism, Primal/Animist/Folk religion, Spiritism, African-derived, some other religion, or no religion/atheist/agnostic; precise response categories varied by country [85]. Racial/ethnic identity was assessed in some, but not all, countries, with response categories varying by country.

### Statistical analysis

Descriptive statistics for the full sample, weighted to be nationally representative within each country, were estimated for each demographic variable. Nationally representative means for SRH were estimated separately for each country and ordered from highest to lowest along with 95% confidence intervals, standard deviations, and Gini coefficients which represent the level of inequality in the SRH variable [89]. Variations in means for SRH across demographic categories were estimated, with all analyses initially conducted by country (see Additional file 1). Primary results consisted of a random effects meta-analysis of country-specific means of SRH in each specific demographic category [90, 91], along with 95% confidence intervals, standard errors, upper and lower limits of a 95% prediction interval across countries, heterogeneity (τ), and *I*^2^ for evidence concerning variation within a particular demographic variable across countries [92]. Forest plots of estimates are available in Additional file 1. This approach was chosen over multilevel or hierarchical modeling because those techniques assume measurement invariance across countries. This was not tenable for the GFS countries, which are essentially a series of 22 separate but coordinated cohort studies carried out in 22 different countries with unique characteristics. The random effects meta-analysis allowed for the pooling of results across countries with heterogeneity maintained at the primary level of analysis. All primary analyses were conducted separately by country, and then, the summary statistics were pooled as if meta-analyzing 22 similar but separate cohort studies. The meta-analysis was conducted in **R** (R Core Team, 2024) using the *metafor* package [93]. Within each country, a global test of variation of SRH across levels of each particular demographic variable was conducted, and a pooled *p*-value across countries was reported concerning evidence for variation within any country [94]. Bonferroni corrected *p*-value thresholds were provided based on the number of demographic variables [95, 96]. Country-specific means in SRH were estimated by religious tradition and race/ethnicity whenever the variables were available with results available in Additional file 1, but these variables were not included in the meta-analysis because the observed response categories varied by country. As a supplementary analysis, a population-weighted meta-analysis was also conducted. All analyses were pre-registered with COS prior to data access (https://osf.io/2z356) and code to reproduce the analyses is openly available in an online COS repository (https://osf.io/vbype) [86, 97].

### Missing data

Missing data on all variables was imputed using multivariate imputation by chained equations, and five imputed datasets were used [98, 99]. To account for variation in the assessment of certain variables across countries (e.g., religious tradition and race/ethnicity), the imputation process was conducted separately in each country. This within-country imputation approach ensured that the imputation models accurately reflected country-specific contexts and assessment methods. Sampling weights were included in the imputation models to account for specific variable missingness that may have been related to the probability of inclusion in the study.

### Accounting for complex sampling design

The GFS used different sampling designs across countries based on the availability of existing panels and recruitment needs [83]. All analyses accounted for the complex survey design by including weights, primary sampling units, and strata.

## Results

### Descriptive statistics

Table 1 provides descriptive statistics for all study variables for the 22 countries combined. Participant ages ranged from the entire adult lifespan (18–80 +). The gender distribution was nearly balanced with 51% female, 49% male, and a small representation from other gender identities (< 1%). A majority of participants were married (53%), 39% were employed by an employer, and 57% attained 9–15 years of education. Regular attendance at religious services varied, with the largest category representing never attend (37%), followed by a few times a year (20%), once a week (19%), more than once a week (13%), and 1–3 times a month (10%). Roughly 94% of participants were native-born. The USA accounted for the largest percentage of the full sample (19%), while Türkiye had the smallest at 1%. Additional file 1: Tables S1a–S22a provide nationally representative descriptive statistics for each country separately.

**Table 1:** Nationally representative descriptive statistics of the observed sample

Variable	Proportion	Frequency
Age		
18–24	0.13	27,007
25–29	0.10	20,700
30–39	0.20	40,256
40–49	0.17	34,464
50–59	0.16	31,793
60–69	0.14	27,763
70–79	0.08	1776
80 or older	0.02	4119
Missing	0.00	20
Gender		
Male	0.49	98,411
Female	0.51	103,488
Other	0.00	602
Missing	0.00	397
Marital status		
Single/never been married	0.26	52,115
Married	0.53	107,354
Separated	0.03	5195
Divorced	0.06	11,654
Widowed	0.05	9823
Domestic partner	0.07	14,931
Missing	0.01	1826
Employment		
Employed for an employer	0.39	78,815
Self-employed	0.18	36,362
Retired	0.14	29,303
Student	0.05	10,726
Homemaker	0.11	21,677
Unemployed and looking for a job	0.08	16,790
None of these/other	0.04	8431
Missing	0.00	793
Education		
Up to 8 years	0.22	45,078
9–15 years	0.57	115,096
16+ years	0.21	42,578
Missing	0.00	146
Service attendance		
>1/week	0.13	26,537
1/week	0.19	39,157
1–3/month	0.10	19,749
A few times a year	0.20	41,436
Never	0.37	75,297
Missing	0.00	722
Immigration status		
Born in this country	0.94	190,998
Born in another country	0.05	9791
Missing	0.01	2110
Country		
Argentina	0.03	6724
Australia	0.02	3844
Brazil	0.07	13,204
Egypt	0.02	4729
Germany	0.05	9506
Hong Kong	0.01	3012
India	0.06	12,765
Indonesia	0.03	6992
Israel	0.02	3669
Japan	0.10	20,543
Kenya	0.06	11,389
Mexico	0.03	5776
Nigeria	0.03	6827
Philippines	0.03	5292
Poland	0.05	10,389
South Africa	0.01	2651
Spain	0.03	6290
Sweden	0.07	15,068
Tanzania	0.04	9075
Türkiye	0.01	1473
UK	0.03	5368
USA	0.19	38,312

Country-specific descriptive statistics are available in Additional file 1

### Levels of SRH across countries

Table 2 shows average levels of SRH for each country in descending order. Indonesia had the highest mean (8.29; 95% CI 8.22, 8.36), with three additional countries having means of 8.00 or higher: Nigeria (8.27; 95% CI 8.17, 8.37), Israel (8.08; 95% CI 7.94, 8.22), and Kenya (8.06; 95% CI 7.98, 8.15). The five lowest were all economically developed countries: Germany (6.62; 95% CI 6.57, 6.68), Australia (6.49; 95% CI 6.41, 6.58), Sweden (6.41; 95% CI 6.37, 6.45), the UK (6.37; 95% CI 6.28, 6.46), and Japan (5.97; 95% CI 5.94, 6.01). Standard deviations were lowest in Israel (1.89), Poland (1.90), and the USA (1.93), and highest in Egypt (2.80), Tanzania (3.02), and India (3.42). Indonesia had the lowest Gini coefficient (0.12), suggesting that SRH was more evenly dispersed among a wider range of survey participants compared with countries that had a higher Gini, such as Türkiye (0.22), Egypt (0.23), and India (0.26).

**Table 2:** Ordered means of self-rated physical health for each country

Country	Mean	LCI	UCI	SD	Gini
Indonesia	8.29	8.22	8.36	1.97	0.12
Nigeria	8.27	8.17	8.37	2.19	0.13
Israel	8.08	7.94	8.22	1.89	0.12
Kenya	8.06	7.98	8.15	2.66	0.16
Mexico	7.79	7.72	7.85	1.96	0.13
Tanzania	7.78	7.66	7.89	3.02	0.19
Philippines	7.69	7.62	7.76	2.14	0.15
Poland	7.68	7.57	7.79	1.90	0.14
South Africa	7.52	7.37	7.68	2.72	0.19
Argentina	7.26	7.18	7.33	2.19	0.16
Brazil	7.16	7.11	7.21	2.31	0.18
Hong Kong	7.12	7.03	7.22	1.99	0.15
India	7.00	6.92	7.09	3.42	0.26
Spain	6.84	6.77	6.90	2.07	0.16
Egypt	6.81	6.71	6.92	2.80	0.23
USA	6.81	6.76	6.86	1.93	0.15
Türkiye	6.70	6.52	6.88	2.72	0.22
Germany	6.62	6.57	6.68	2.10	0.17
Australia	6.49	6.41	6.58	2.06	0.17
Sweden	6.41	6.37	6.45	2.07	0.18
UK	6.37	6.28	6.46	2.29	0.20
Japan	5.97	5.94	6.01	2.09	0.19

### Demographic correlates of SRH

Table 3 shows results from a random effects meta-analysis of country-specific means of SRH for all 22 countries for each demographic category with every country given equal weight regardless of population size (population-weighted analyses are provided in Additional file 1: Table S23 and discussed below). Means, 95% confidence intervals (CI), standard errors (SE), lower (LL) and upper (UL) prediction intervals, heterogeneity (τ), *I*^2^, and global *p*-values were computed separately for each demographic variable.

**Table 3:** Random effects meta-analysis of self-rated physical health means by demographic categories (pooled across 22 countries)

					Prediction interval				
Variable	Category	Est	95% CI	SE	LL	UL	Heterogeneity (τ)	*I*^2^	Global *p*-value
Age group									<.001**
	18–24	7.62	(7.29,7.96)	0.17	6.26	8.78	0.79	98.9	
	25–29	7.51	(7.17,7.84)	0.17	6.14	8.65	0.78	98.6	
	30–39	7.36	(7.02,7.69)	0.17	5.99	8.60	0.79	99.3	
	40–49	7.12	(6.83,7.42)	0.15	5.79	8.31	0.70	98.8	
	50–59	6.93	(6.66,7.21)	0.14	5.74	7.92	0.64	98.5	
	60–69	6.72	(6.44,7.01)	0.15	5.72	7.84	0.66	98.3	
	70–79	6.55	(6.26,6.85)	0.15	5.18	7.61	0.67	97.6	
	80 or older	6.44	(6.21,6.67)	0.12	5.25	7.11	0.42	78.8	
Gender									<.001**
	Male	7.34	(7.06,7.63)	0.15	5.85	8.28	0.68	99.5	
	Female	7.09	(6.81,7.38)	0.15	6.09	8.30	0.68	99.5	
	Other	6.31	(5.90,6.72)	0.21	5.46	7.33	0.56	60.0	
Marital status									<.001**
	Married	7.24	(6.97,7.50)	0.13	6.17	8.35	0.63	99.5	
	Separated	6.93	(6.61,7.26)	0.17	5.88	8.12	0.72	93.1	
	Divorced	6.80	(6.48,7.11)	0.16	5.62	7.91	0.71	96.4	
	Widowed	6.58	(6.29,6.87)	0.15	5.35	7.88	0.66	94.0	
	Domestic partner	7.22	(6.89,7.55)	0.17	5.56	8.22	0.72	98.0	
	Single, never married	7.34	(6.96,7.73)	0.20	5.63	8.59	0.92	99.5	
Employment status									<.001**
	Employed for an employer	7.41	(7.12,7.70)	0.15	6.03	8.38	0.68	99.5	
	Self-employed	7.42	(7.17,7.67)	0.13	6.00	8.35	0.59	98.2	
	Retired	6.62	(6.36,6.88)	0.13	5.79	7.77	0.59	97.8	
	Student	7.61	(7.26,7.96)	0.18	6.35	9.04	0.83	98.0	
	Homemaker	6.88	(6.56,7.19)	0.16	5.68	8.35	0.73	97.9	
	Unemployed and looking for a job	6.92	(6.46,7.37)	0.23	4.89	8.32	1.07	98.7	
	None of these/other	6.45	(5.96,6.93)	0.25	3.80	8.37	1.11	97.3	
Education									<.001**
	Up to 8 years	6.95	(6.63,7.27)	0.16	5.28	8.25	0.74	98.2	
	9–15 years	7.30	(6.96,7.63)	0.17	5.88	8.55	0.80	99.7	
	16+ years	7.50	(7.24,7.77)	0.13	6.33	8.80	0.62	99.2	
Religious service attendance									<.001**
	>1/week	7.54	(7.24,7.83)	0.15	6.42	9.16	0.70	98.0	
	1/week	7.48	(7.27,7.69)	0.11	6.64	8.32	0.48	97.3	
	1–3/month	7.27	(7.05,7.48)	0.11	6.18	8.17	0.50	95.5	
	A few times a year	7.18	(6.94,7.43)	0.13	6.07	8.02	0.59	98.6	
	Never	6.95	(6.67,7.23)	0.14	5.92	8.31	0.64	99.3	
Immigration status									<.001**
	Born in this country	7.21	(6.92,7.50)	0.15	5.97	8.29	0.70	99.7	
	Born in another country	7.14	(6.87,7.41)	0.14	6.12	8.12	0.59	93.8	

*p < .05; **p < .007 (Bonferroni corrected threshold)

There was a progressive decline in SRH with age: mean scores decreased from 7.62 (95% CI 7.29, 7.96) for the 18–24 age group to 6.44 (95% CI 6.21, 6.67) for those aged 80 or older. Some neighboring categories were not significantly different, but there were meaningful differences between younger versus older age groups. Females reported slightly lower SRH compared with men (7.09 and 7.34, respectively), but the 95% confidence intervals overlapped (CI 7.06, 7.63 for males and CI 6.81, 7.38 for females). Single/never married individuals reported the highest SRH (mean: 7.34, 95% CI 6.96, 7.73), closely followed by married (mean: 7.24, 95% CI 6.97, 7.50) and domestic partnerships (mean: 7.22, 95% CI 6.89, 7.55). The lowest score was for widowed (mean: 6.58, 95% CI 6.29, 6.87). Based on confidence intervals, the only significant differences were for widowed compared with single/never married, married, and domestic partners. When considering employment status, three groups—students (mean: 7.61, 95% CI 7.26, 7.96), self-employed (Mean: 7.42, 95% CI 7.17, 7.67), and employed for an employer (mean: 7.41, 95% CI 7.12, 7.70)—had relatively high scores. These were followed by unemployed and looking for a job (mean: 6.92, 95% CI 6.46, 7.37), homemakers (mean 6.88, 95% CI 6.56, 7.19), and retired (mean: 6.62, 95% CI 6.36, 6.88). The lowest SRH was observed for none of these/other (mean: 6.45, 95% CI 5.96, 6.93). The means for the top three groups were considerably larger compared with retired and none of these/other. SRH showed a slight increase across education (from 6.95 for up to 8 years to 7.50 for 16 + years), but these results were not significant based on overlapping confidence intervals. Religious service attendance was positively correlated with SRH. The mean for never attending was 6.95 (95% CI 6.67, 7.23), which was significantly lower than the means for attending once a week (7.48, 95% CI 7.27, 7.69) and more than once a week (7.54, 95% CI 7.24, 7.83). The difference in immigration status was comparatively small.

Additional file 1: Table S23 complements these results by providing a population-weighted meta-analysis, where each country’s results were weighted according to its 2023 population size (i.e., India had a greater influence on the results since it was the largest country). Compared with Table 3, the patterns for age, gender, employment, education, religious service attendance, and immigration status were comparable although the means were somewhat different. The results for marital status were slightly different. In Table 3, the highest SRH was observed for single/never married, but in the population-weighted findings, the highest was for married.

### Differences in demographic correlates of SRH across countries

Table 3 also provides information about variations in these associations across countries. The global *p*-values for each set of demographic characteristics were significant beyond the Bonferroni corrected threshold of 0.007, indicating evidence that each set of variables was associated with SRH in at least one country (not necessarily all countries, however). The heterogeneity (τ) statistics provide an estimate of how much the mean of SRH in each specific demographic category varied across countries, with larger numbers indicating more heterogeneity. *I*^2^ statistics are also provided for interested readers.

When evaluating age groups, τ values were 0.78–0.79 for those 39 and under, 0.64–0.70 for individuals 40–79, and 0.42 for the 80 or older age category. This means that SRH varied more across countries in younger age groups compared with older ones and that age may become a more consistent predictor of SRH in the later years of life. For gender, τ estimates were identical for both males and females (0.68), suggesting a moderate level of heterogeneity across countries. The τ for every category of marital status was between 0.63 and 0.72 except for one: single/never married (τ = 0.92). This larger estimate suggests more variation in SRH across countries for individuals in this category. There was a wide range of heterogeneity estimates for employment status, ranging from 0.59 for self-employed and retired to 1.11 for none of these/others. Based on these results, SRH varied more across countries for unemployed and looking for a job and none of these/others. For education, τ ranged from 0.62 for 16 + years to 0.80 for 9–15 years, with up to 8 years being 0.74, suggesting that SRH varied more across countries among individuals with 15 or fewer years of education. With respect to religious service attendance, there was more variation in SRH across countries for individuals who attended more than once a week (τ = 0.70). Finally, the τ for non-immigrants and immigrants were 0.70 and 0.59, respectively, which suggests that SRH was more similar across countries for immigrants compared with non-immigrants.

Additional file 1: Tables S1b–S22b mirror Table 3 but show findings for each country separately (see Figures S1–34 as well). These provide additional insight into country-specific variations in SRH across demographic characteristics. Key findings from these tables and further discussion of variation across countries and which countries differ from the meta-analytic patterns are discussed below.

## Discussion

Relatively few studies have used nationally representative data to examine how SRH varies across: (a) countries around the world and (b) demographic characteristics in diverse nations and cultures [15, 25, 32, 38]. The current study addresses these issues by analyzing data from the first wave of the GFS. Three of the four largest SRH means were found in developing, non-Western countries (Indonesia, Nigeria, and Kenya), while the five lowest were in economically developed countries (Germany, Australia, Sweden, the UK, and Japan). Confidence intervals for some countries overlapped, and language translation issues mean that question-wording may have different connotations across countries despite a concerted effort by Gallup to minimize these effects, so these differences should not be overstated or interpreted as precise rankings. They do suggest, however, that high per capita income does not necessarily translate into better SRH. Countries also differed in variation around the mean. SRH was more evenly dispersed in countries like Israel, Poland, and the USA and more unequally distributed in places like Egypt, Tanzania, and India. SRH also varied across demographic characteristics. Results from a random effects meta-analysis of all 22 countries showed that SRH was associated with each demographic characteristic in at least one country. As shown in Tables S1b–S22b and Figures S1–34 in Additional file 1, however, patterns differed substantially across countries.

Existing research shows that age is associated with SRH [21, 25, 58], and in the GFS data, it was correlated with SRH in every nation except Brazil. Looking across individual countries, several followed the overall (and perhaps expected) general trend of decreasing SRH as age increased that was shown in the meta-analysis. Notable exceptions were (a) Australia, Japan, and Sweden, where the highest SRH was reported by both younger and older individuals compared with those in the middle categories (a *U*-shaped relationship); (b) Hong Kong, where no clear pattern existed; and (c) the USA, where the highest SRH scores were for the three oldest age groups. The latter finding is unique to the USA but has also been reported in a previous study [100], so future research should identify factors that promote SRH among older adults in the USA in hopes of using this knowledge in other countries. Possible explanations include financial security, more frequent religious attendance, and adequate leisure time. In addition, a recent study in the USA found that physical health and functional limitations become weaker predictors of SRH as individuals age, while social factors become more salient [21]. No studies have examined this possibility in other countries, but future cross-national research of this type may identify mechanisms that promote SRH across the entire life course and around the world.

For gender, there were significant differences in all countries except Australia, Germany, Hong Kong, Indonesia, Kenya, Nigeria, the Philippines, Poland, South Africa, the UK, and the USA. Within individual countries, there was a general trend of males reporting higher SRH than females. This was found in Argentina, Brazil, Egypt, India, Israel, Mexico, Spain, Sweden, Tanzania, and Türkiye. Seven of these countries are developing, non-Western nations, where life may be difficult for women. For example, women in developing countries often face higher risks of maternal mortality, receive inadequate healthcare, lack access to prenatal and postnatal services, and suffer from malnutrition [101]. Gender-based violence [102] and discrimination [103] may also be more common. One noteworthy exception to the overall trend was Japan, where females reported higher SRH than males. Possible explanations may include perceptions of resilience or good health even in the face of challenges, interpreting longer life expectancy as good health, relatively high socioeconomic status, and pressure to present themselves as healthy regardless of actual health status [104, 105]. Future research should examine these possibilities in detail. Overall, economic development seems to play a role in shaping gender equality in SRH, but it is clearly not the only factor that matters.

Previous research shows that marital status is associated with health [106, 107], and in the current data, there was strong evidence of correlation with SRH in every country except Argentina, Indonesia, Mexico, the Philippines, Spain, and Türkiye. Broad trends in the meta-analysis included single/never been married, married, and domestic partnerships tending to report somewhat higher SRH compared with separated, divorced, or widowed. A similar pattern was observed in several countries. In some, the highest SRH was reported by married (e.g., Australia, Brazil, Hong Kong, Japan, Sweden, the UK, and the USA), while in others, it was higher in single/never been married or domestic partner (e.g., Egypt, Germany, India, Israel, Kenya, Nigeria, Poland, South Africa, Spain, and Tanzania). For some of these differences, the statistical evidence was more limited, however. In general, these results are consistent with research showing that separated, divorced, or widowed individuals may be at risk for poor health [106, 108]. Marital status appears to shape health through a variety of mechanisms including social connections and support, risky behaviors, economic and material conditions, psychological well-being, and access to healthcare and insurance, among others [106]. From a global perspective, marital status is embedded in broader social institutions and contexts (e.g., beliefs and practices regarding gender roles, expectations for traditional family life, religious contexts, educational and economic opportunities) that may moderate its association with health and well-being [109–111], and many of the mechanisms may vary across nations and cultures. Future research should conduct detailed examinations of how different cultural and structural factors across nations shape the relationship between marital status and SRH.

There was notable evidence of an association between employment status and SRH in every country except Indonesia, Mexico, Nigeria, and the Philippines (all developing nations). The meta-analysis results suggested that SRH was highest among students, the self-employed, and those employed by an employer, and lowest among retired and none of these/others. These broad trends were present in numerous individual countries as well. The results for retired could be tied to age since a control was not included for this potentially confounding variable. A notable exception to these broad trends was retired not being among the lowest SRH scores in Australia, Brazil, Sweden, and the USA. This could be due to adequate retirement systems that provide financial security and access to healthcare (at least in some of these countries). However, other countries in the sample meet these criteria as well, so future research should attempt to shed light on these findings. Overall, these results are consistent with previous research showing that employment is associated with better health [112].

In line with existing studies [113, 114], education was a significant correlate of SRH in most countries with the exception of Brazil, Indonesia, Mexico, Nigeria, and the Philippines (all developing nations). In many countries—including Argentina, Egypt, Germany, India, Japan, Poland, South Africa, Spain, Sweden, Tanzania, Türkiye, the UK, and the USA—SRH was highest in the 16 + years of education category. These countries are diverse with respect to regions of the world, economic conditions, majority religious tradition, etc., so education clearly matters in many different contexts and cultures. In Australia, SRH was highest among those with the lowest and highest levels of education. In Hong Kong, the lowest SRH was observed for the 16 + education category, perhaps due to greater job demands, stress, work hours, and burnout that may accompany occupations with high levels of education and skills [115]. Future research should seek to identify unique aspects of life in Hong Kong that may explain this finding. Together, these results suggest that higher levels of education are associated with better health in most, but not all, countries and contexts.

With respect to religious service attendance, significant associations were found in every country except India, Indonesia, Mexico, South Africa, Tanzania, and Türkiye, primarily developing nations. Similar to the overall findings from the meta-analysis, in most cases more frequent attendance was associated with better SRH. This is consistent with the majority of existing studies on this topic [116]. The general trend of higher SRH among those who attended was even present in some “secular” nations such as Sweden and the UK. In contrast, the lowest levels of SRH were observed among individuals who attended more than once a week in Poland [117]. In predominantly Catholic countries like Poland, suffering and poor health may be seen as an inevitable or even virtuous part of life, potentially influenced by doctrines around the acceptance of suffering. In other words, some religious teachings may emphasize the redemptive value of suffering, which could lead individuals to underreport their health status or accept poor health as part of their faith [118]. Future research will be needed to confirm these processes, however.

Immigration has recently been conceptualized as a social determinant of health [119]. In the current study, however, differences in SRH were not especially large regarding immigration in the meta-analysis, with evidence of association with SRH only in a minority of countries (and these associations appear complex). In some countries including Australia, Germany, Spain, and the UK, immigrants had higher SRH. In contrast, SRH was lower among immigrants in Hong Kong, Israel, and the Philippines. This pattern of null and contrasting results may explain the weak findings for this variable in the meta-analysis. Immigrants routinely face challenges in their new home countries including finding employment and housing, gaining access to health insurance, and experiencing harmful rhetoric from non-immigrants and intimidation in healthcare settings [120], all of which could undermine their health and explain the findings for some countries. In contexts where immigrants report better health, this may be due to selection effects where healthy people are more likely to migrate, desirable health behaviors are more common among migrants compared with the native population, and social and cultural factors such as strong family and communities bonds are higher among immigrants [121, 122]. Given that immigrants report better health in some countries and worse in others, future research should carefully examine both cases.

Religious tradition and race/ethnicity were not included in the meta-analysis, but they were examined in the country-specific analyses. In many cases, there were a large number of categories for each, so summarizing the results was difficult. The full results are provided in Tables S1b–S22b, but here is a brief summary. Religious tradition was associated with differences in SRH in many diverse countries including Australia, Brazil, Germany, Hong Kong, India, Indonesia, Japan, Kenya, Mexico, Nigeria, the Philippines, Poland, South Africa, Spain, Sweden, Tanzania, Türkiye, the UK, and the USA. It is important to note, however, that there may also be significant religious differences in countries by “denomination” or other indicators of group membership that were not captured by the current analyses. For a review of existing research in this area, see the *Handbook of Religion and Health* [116]. Race/ethnicity was significant in Australia, Egypt, Kenya, the Philippines, South Africa, and Türkiye (data was not available in Germany, Japan, Spain, and Sweden). Moving forward, religion and race scholars with expertise in each nation should examine these findings in detail and offer insights based on their knowledge of local cultures and contexts.

This study has several strengths. First, it draws on a new data source that includes nationally representative samples in 22 countries that were collected in 2022–2023. We know relatively little about several of the countries in the study, so these findings provide new insights into variations in SRH around the world that can supplement work that has already been done [25, 28, 104, 123, 124]. Second, all survey items included in the study, including SRH, were carefully chosen and evaluated by leading scholars from around the world, and then extensively pretested by Gallup personnel on the ground in each country [83–85]. This helped to ensure that the data met the highest standards for relevance and reliability. Third, the GFS is a large survey that contains nationally representative samples, so the estimates of SRH should be reasonably accurate. Fourth, the sample includes countries that are (a) geographically dispersed across North America, South America, Europe, Asia, and Australia/Oceania; (b) both economically developed and developing; and (c) culturally and religiously diverse, including countries with majorities of several major religious traditions including Christianity, Islam, Hinduism, and Judaism. This means that the findings are relevant to diverse individuals and groups of people around the world.

Despite these strengths, this study also has limitations. First, global research is difficult for many reasons including language barriers and translation issues [125], and these were present during the GFS data collection process. As described in the Methodology and Translations documents [83], Gallup conducted extensive pretesting and translation work in hopes of obtaining comparable meanings of survey items across countries, but this may not always be possible. Some words and concepts simply do not have clear analogs in different languages and countries. Different cultures also have distinct ways of viewing health, as well as different norms regarding speaking or reporting about a variety of issues [22, 23]. Further, at least one study suggests that macro-level contextual factors like individualism/collectivism may moderate the connection between social relations and health across countries [33]. The same may be true for additional contextual variables and individual-level correlates of health, so future research should examine these possibilities. The unique diversity in the GFS is one of its key strengths, but it also makes interpreting the findings in a fair and objective way difficult. Together, this means that direct, strict comparisons of statistics across countries should be made with caution. This is also necessary when interpreting cross-national differences because of different modes of assessment, differing interpretations of response scales, and seasonal effects arising from data being collected in different countries at different times of the year. Second, this study is cross-sectional and only analyzes the first wave of GFS data. This baseline survey was released on 2/13/24, and data collection for the second wave is ongoing. The findings reported here provide a descriptive snapshot at one point in time and should not be interpreted causally. Third, due to the scale of the project, it was not possible to include large, multi-item measures in the survey. Fourth, this study only examined individual-level variables. As discussed below, contextual and country-level factors may also be important and should be the topic of follow-up research.

Future work should build on these findings in several ways. First, scholars conducting independent research in each country should seek to compare their findings with those reported here in hopes of better understanding the dynamics that shape SRH in each country. This is beyond the scope of this paper but would be valuable for future studies. Second, subsequent research should attempt to determine why SRH is relatively high in countries such as Indonesia and low in ones like Japan. These findings do not necessarily align with life expectancy across countries, so teasing out the reasons for different reports of SRH should be a topic of future research. If possible, attempts should be made to identify causes (e.g., real differences in morbidity, varying population-based reference groups, differences in survey question interpretation, and willingness to talk about sensitive issues like health). Previous research has also linked contextual factors including the level of government involvement in population health, GDP, income inequality, social capital, and individualism/collectivism with health and well-being across nations [33, 77–79], and these may offer fruitful areas of inquiry for future research seeking to understand the causes of cross-national variation. Third, follow-up research with at least two waves of GFS data will be better able to examine how various factors, including the demographic variables analyzed here, shape longitudinal trends in SRH. The primary goal of the GFS is to identify causal mechanisms underlying differences in human flourishing around the world, and this should be a focus of future work as soon as two waves of data are available. Fourth, we need more research on developing countries that do not have Christian majorities. The current study is among the first to report findings from large nationally representative samples in several countries, and these results are the foundation for future work. Fifth, authors should seek to convey their findings to audiences beyond other scholars, such as governmental agencies around the world, non-governmental organizations, and various media outlets. For this work to have a positive impact on the lives of people, it needs to reach those with the resources, power, and desire to make the world a better place.

## Conclusions

SRH appears to vary across countries. Three of the four highest SRH means were found in developing, non-Western countries, while the five lowest were in economically developed nations. SRH also varies across key demographic characteristics including age, gender, marital status, employment, education, religious service attendance, and immigration status in at least some countries. There was considerable heterogeneity across nations, however, suggesting that country-level contexts are important factors that shape these relationships.

## Supplementary Information

Additional file 1: Demographic Variation in Self-Rated Physical Health Across 22 Countries: Findings from the Global Flourishing Study. Table S1a: Nationally-Representative Descriptive Statistics of the Observed Sample (Argentina). Table S1b: Variations Across Demographic Characteristics (Argentina). Table S2a: Nationally-Representative Descriptive Statistics of the Observed Sample (Australia). Table S2b: Variations Across Demographic Characteristics (Australia). Table S3a: Nationally-Representative Descriptive Statistics of the Observed Sample (Brazil). Table S3b: Variations Across Demographic Characteristics (Brazil). Table S4a: Nationally-Representative Descriptive Statistics of the Observed Sample (Egypt). Table S4b: Variations Across Demographic Characteristics (Egypt). Table S5a: Nationally-Representative Descriptive Statistics of the Observed Sample (Germany). Table S5b: Variations Across Demographic Characteristics (Germany). Table S6a: Nationally-Representative Descriptive Statistics of the Observed Sample (Hong Kong). Table S6b: Variations Across Demographic Characteristics (Hong Kong). Table S7a: Nationally-Representative Descriptive Statistics of the Observed Sample (India). Table S7b: Variations Across Demographic Characteristics (India). Table S8a: Nationally-Representative Descriptive Statistics of the Observed Sample (Indonesia). Table S8b: Variations Across Demographic Characteristics (Indonesia). Table S9a: Nationally-Representative Descriptive Statistics of the Observed Sample (Israel). Table S9b: Variations Across Demographic Characteristics (Israel). Table S9b: Variations Across Demographic Characteristics (Israel). Table S10b: Variations Across Demographic Characteristics (Japan). Table S11a: Nationally-Representative Descriptive Statistics of the Observed Sample (Kenya). Table S11b: Variations Across Demographic Characteristics (Kenya). Table S12a: Nationally-Representative Descriptive Statistics of the Observed Sample (Mexico). Table S12b: Variations Across Demographic Characteristics (Mexico). Table S13a: Nationally-Representative Descriptive Statistics of the Observed Sample (Nigeria). Table S13b: Variations Across Demographic Characteristics (Nigeria). Table S14a: Nationally-Representative Descriptive Statistics of the Observed Sample (Philippines). Table S14b: Variations Across Demographic Characteristics (Philippines). Table S15a: Nationally-Representative Descriptive Statistics of the Observed Sample (Poland). Table S15b: Variations Across Demographic Characteristics (Poland). Table S16a: Nationally-Representative Descriptive Statistics of the Observed Sample (South Africa). Table S16b: Variations Across Demographic Characteristics (South Africa). Table S17a: Nationally-Representative Descriptive Statistics of the Observed Sample (Spain). Table S17b: Variations Across Demographic Characteristics (Spain). Table S18a: Nationally-Representative Descriptive Statistics of the Observed Sample (Sweden). Table S18b: Variations Across Demographic Characteristics (Sweden). Table S19a: Nationally-Representative Descriptive Statistics of the Observed Sample (Tanzania). Table S19b: Variations Across Demographic Characteristics (Tanzania). Table S19b: Variations Across Demographic Characteristics (Tanzania). Table S20b: Variations Across Demographic Characteristics (Türkiye). Table S21a: Nationally-Representative Descriptive Statistics of the Observed Sample (United Kingdom). Table S21b: Variations Across Demographic Characteristics (United Kingdom). Table S22a: Nationally-Representative Descriptive Statistics of the Observed Sample (United States). Table S22b: Variations Across Demographic Characteristics (United States). Table S23. Population weighted meta-analysis of results demographic group means. Figure S1. Forest plot for “Age group”-“18–24.” Figure S2. Forest plot for “Age group”-“25–29.” Figure S3. Forest plot for “Age group”-“30–39.” Figure S4. Forest plot for “Age group”-“40–49.” Figure S5. Forest plot for “Age group”-“50–59.” Figure S6. Forest plot for “Age group”-“60–69.” Figure S7. Forest plot for “Age group”-“70–79.” Figure S8. Forest plot for “Age group”-“80 or older.” Figure S9. Forest plot for “Gender”-“Male.” Figure S10. Forest plot for “Gender”-“Female.” Figure S11. Forest plot for “Gender”-“Other.” Figure S12. Forest plot for “Marital status”-“Single, never married.” Figure S13. Forest plot for “Marital status”-“Married.” Figure S14. Forest plot for “Marital status”-“Separated.” Figure S15. Forest plot for “Marital status”-“Divorced.” Figure S16. Forest plot for “Marital status”-“Widowed.” Figure S17. Forest plot for “Marital status”-“Domestic partner.” Figure S18. Forest plot for “Employment status”-“Employed for an employer.” Figure S19. Forest plot for “Employment status”-“Self-employed.” Figure S20. Forest plot for “Employment status”-“Retired.” Figure S21. Forest plot for “Employment status”-“Student.” Figure S22. Forest plot for “Employment status”-“Homemaker.” Figure S23. Forest plot for “Employment status”-“Unemployed and looking for a job.” Figure S24. Forest plot for “Employment status”-“None of these/others.” Figure S25. Forest plot for “Education status”-“Up to 8 years.” Figure S26. Forest plot for “Education status”-“9–15 years.” Figure S27. Forest plot for “Education status”-“16+ years.” Figure S28. Forest plot for “Religious service attendance”-“>1/week.” Figure S29. Forest plot for “Religious service attendance”-“1/week.” Figure S30. Forest plot for “Religious service attendance”-“1–3/week.” Figure S31. Forest plot for “Religious service attendance”-“A few times a year.” Figure S2. Forest plot for “Religious service attendance”-“Never.” Figure S33. Forest plot for “Immigration status”-“Born in this country.” Figure S34. Forest plot for “Immigration status”-“Born in another country.”

## Data Availability

Data for Wave 1 of the GFS is available through the Center for Open Science (https://www.cos.io/gfs) upon submission of a pre-registration, and will be openly available without pre-registration beginning February 2025. Subsequent waves of the GFS will similarly be made available. Please see https://www.cos.io/gfs-access-data for more information about data access. All analyses were pre-registered with COS prior to data access (https://osf.io/2z356). Code to reproduce the analyses is openly available in an online COS repository (https://osf.io/vbype).
