# Supplementary material for: Demographic variation in self-rated physical health across 22 countries: findings from the Global Flourishing Study
Source: BMC Glob Public Health. 2025 Apr 30;3:38. doi: 10.1186/s44263-025-00141-1 (PMC12042321; doi:10.1186/s44263-025-00141-1)

**Additional File 1 for Demographic Variation in Self-Rated Physical Health Across 22 Countries: Findings from the Global Flourishing Study**

Table a for each country shows weighted descriptive statistics for all independent variables examined in this study using nonimputed Wave 1 data. These include age, gender, marital status, employment, education, religious service attendance, immigration status, and religion.

Table b for each country shows weighted means/proportions, standard errors, and 95% confidence intervals across all of the demographic variables examined in this study using Wave 1 data. It also includes global F (Wald) test probabilities for the overall joint significance of each set of indicators for the demographic variables. These results were estimated using five imputed datasets. The multiple imputation process included all study variables and sampling weights.

*Note: Estimates for variables with df<1 were excluded from the tables because of inflated standard errors and confidence intervals.*

**Table S1a: Nationally-Representative Descriptive Statistics of the Observed Sample (Argentina)**

| Variable | Proportion | Frequency |
| --- | --- | --- |
| Age |  |  |
| 18-24 | 0.16 | 1108 |
| 25-29 | 0.11 | 719 |
| 30-39 | 0.21 | 1432 |
| 40-49 | 0.19 | 1254 |
| 50-59 | 0.15 | 1014 |
| 60-69 | 0.11 | 730 |
| 70-79 | 0.05 | 356 |
| 80 or Older | 0.02 | 112 |
| Missing | . | . |
| Gender |  |  |
| Male | 0.47 | 3143 |
| Female | 0.53 | 3542 |
| Other | 0.00 | 21 |
| Missing | 0.00 | 18 |
| Marital Status |  |  |
| Single/Never Been Married | 0.35 | 2381 |
| Married | 0.23 | 1565 |
| Separated | 0.07 | 455 |
| Divorced | 0.05 | 321 |
| Widowed | 0.06 | 401 |
| Domestic Partner | 0.23 | 1514 |
| Missing | 0.01 | 88 |
| Employment |  |  |
| Employed for an Employer | 0.36 | 2440 |
| Self-Employed | 0.26 | 1748 |
| Retired | 0.11 | 773 |
| Student | 0.05 | 354 |
| Homemaker | 0.10 | 639 |
| Unemployed and Looking for a Job | 0.08 | 569 |
| None of These/Other | 0.03 | 179 |
| Missing | 0.00 | 22 |
| Education |  |  |
| Up to 8 Years | 0.34 | 2263 |
| 9-15 Years | 0.57 | 3823 |
| 16+ Years | 0.09 | 635 |
| Missing | 0.00 | 3 |
| Service Attendance |  |  |
| >1/Week | 0.08 | 532 |
| 1/Week | 0.12 | 773 |
| 1-3/Month | 0.07 | 461 |
| A Few Times a Year | 0.29 | 1949 |
| Never | 0.44 | 2982 |
| Missing | 0.00 | 27 |
| Immigration Status |  |  |
| Born in This Country | 0.94 | 6346 |
| Born in Another Country | 0.05 | 348 |
| Missing | 0.00 | 29 |
| Religion |  |  |
| Christianity | 0.74 | 4992 |
| Islam | 0.00 | 9 |
| Hinduism | 0.00 | 6 |
| Buddhism | 0.01 | 35 |
| Judaism | 0.01 | 40 |
| Sikhism | 0.00 | 0 |
| Baha'i | . | . |
| Jainism | . | . |
| Shinto | . | . |
| Taoism | 0.00 | 2 |
| Confucianism | 0.00 | 0 |
| Primal, Animist, or Folk Religion | 0.00 | 19 |
| Spiritism | . | . |
| African-Derived | . | . |
| Chinese | . | . |
| Some Other Religion | 0.02 | 156 |
| No Religion/Atheist/Agnostic | 0.20 | 1352 |
| Missing | 0.02 | 111 |
| Race/Ethnicity |  |  |
| Asian | 0.01 | 43 |
| Black | 0.01 | 95 |
| Indigenous | 0.02 | 129 |
| Mestizo(a) | 0.27 | 1801 |
| Mullato(a) | 0.01 | 75 |
| White | 0.51 | 3406 |
| Other | 0.02 | 104 |
| Missing | 0.16 | 1070 |

**Table S1b: Variations Across Demographic Characteristics (Argentina)**

| Variable | Mean | SE | LCI | UCI | Global p-value |
| --- | --- | --- | --- | --- | --- |
| Age |  |  |  |  |  |
| 18-24 | 7.45 | 0.10 | 7.25 | 7.66 | 0.00 |
| 25-29 | 7.62 | 0.12 | 7.39 | 7.85 | . |
| 30-39 | 7.36 | 0.08 | 7.20 | 7.51 | . |
| 40-49 | 7.31 | 0.09 | 7.14 | 7.47 | . |
| 50-59 | 7.04 | 0.10 | 6.85 | 7.23 | . |
| 60-69 | 6.88 | 0.13 | 6.62 | 7.14 | . |
| 70-79 | 6.77 | 0.21 | 6.36 | 7.18 | . |
| 80 or Older | 7.07 | 0.32 | 6.44 | 7.71 | . |
| Gender |  |  |  |  |  |
| Male | 7.47 | 0.05 | 7.37 | 7.58 | 0.00 |
| Female | 7.07 | 0.06 | 6.95 | 7.18 | . |
| Other | 6.31 | 0.35 | 5.52 | 7.10 | . |
| Marital Status |  |  |  |  |  |
| Single/Never Been Married | 7.37 | 0.07 | 7.24 | 7.50 | 0.07 |
| Married | 7.15 | 0.08 | 6.99 | 7.31 | . |
| Separated | 7.27 | 0.14 | 6.98 | 7.55 | . |
| Divorced | 7.01 | 0.18 | 6.66 | 7.37 | . |
| Widowed | 6.95 | 0.17 | 6.61 | 7.29 | . |
| Domestic Partner | 7.32 | 0.08 | 7.16 | 7.48 | . |
| Employment |  |  |  |  |  |
| Employed for an Employer | 7.48 | 0.06 | 7.36 | 7.59 | 0.00 |
| Self-Employed | 7.37 | 0.07 | 7.24 | 7.51 | . |
| Retired | 6.69 | 0.14 | 6.42 | 6.96 | . |
| Student | 7.17 | 0.14 | 6.89 | 7.45 | . |
| Homemaker | 7.25 | 0.14 | 6.98 | 7.51 | . |
| Unemployed and Looking for a Job | 7.09 | 0.16 | 6.78 | 7.40 | . |
| None of These/Other | 6.27 | 0.32 | 5.65 | 6.90 | . |
| Education |  |  |  |  |  |
| Up to 8 Years | 7.21 | 0.09 | 7.03 | 7.39 | 0.00 |
| 9-15 Years | 7.24 | 0.04 | 7.16 | 7.32 | . |
| 16+ Years | 7.52 | 0.07 | 7.37 | 7.66 | . |
| Service Attendance |  |  |  |  |  |
| >1/Week | 7.79 | 0.14 | 7.50 | 8.07 | 0.00 |
| 1/Week | 7.45 | 0.11 | 7.23 | 7.67 | . |
| 1-3/Month | 7.08 | 0.16 | 6.76 | 7.40 | . |
| A Few Times a Year | 7.28 | 0.07 | 7.14 | 7.43 | . |
| Never | 7.12 | 0.06 | 7.01 | 7.24 | . |
| Immigration Status |  |  |  |  |  |
| Born in This Country | 7.24 | 0.04 | 7.16 | 7.32 | 0.03 |
| Born in Another Country | 7.56 | 0.15 | 7.27 | 7.85 | . |
| Religion |  |  |  |  |  |
| Christianity | 7.29 | 0.05 | 7.20 | 7.38 | 0.03 |
| Islam | 7.97 | 1.52 | 3.00 | 12.93 | . |
| Hinduism | 7.71 | 0.51 | 5.53 | 9.89 | . |
| Buddhism | 7.51 | 0.28 | 6.88 | 8.13 | . |
| Judaism | 7.81 | 0.31 | 7.17 | 8.46 | . |
| Sikhism | 7.00 | . | . | . | . |
| Baha'i | . | . | . | . | . |
| Jainism | . | . | . | . | . |
| Shinto | . | . | . | . | . |
| Taoism | . | . | . | . | . |
| Confucianism | 9.64 | . | . | . | . |
| Primal, Animist, or Folk Religion | 7.50 | 0.86 | 5.56 | 9.43 | . |
| Spiritism | . | . | . | . | . |
| African-Derived | . | . | . | . | . |
| Chinese | . | . | . | . | . |
| Some Other Religion | 7.00 | 0.33 | 6.35 | 7.65 | . |
| No Religion/Atheist/Agnostic | 7.14 | 0.09 | 6.97 | 7.31 | . |
| Race/Ethnicity |  |  |  |  |  |
| Asian | 8.15 | 0.37 | 7.37 | 8.92 | 0.26 |
| Black | 7.92 | 0.43 | 7.00 | 8.83 | . |
| Indigenous | 7.40 | 0.33 | 6.72 | 8.07 | . |
| Mestizo(a) | 7.28 | 0.08 | 7.14 | 7.43 | . |
| Mullato(a) | 7.11 | 0.55 | 6.01 | 8.21 | . |
| White | 7.22 | 0.05 | 7.11 | 7.32 | . |
| Other | 7.02 | 0.39 | 6.22 | 7.83 | . |

**Table S2a: Nationally-Representative Descriptive Statistics of the Observed Sample (Australia)**

| Variable | Proportion | Frequency |
| --- | --- | --- |
| Age |  |  |
| 18-24 | 0.09 | 345 |
| 25-29 | 0.07 | 282 |
| 30-39 | 0.17 | 641 |
| 40-49 | 0.16 | 618 |
| 50-59 | 0.18 | 691 |
| 60-69 | 0.15 | 589 |
| 70-79 | 0.13 | 498 |
| 80 or Older | 0.05 | 178 |
| Missing | 0.00 | 2 |
| Gender |  |  |
| Male | 0.48 | 1861 |
| Female | 0.50 | 1941 |
| Other | 0.01 | 36 |
| Missing | 0.00 | 6 |
| Marital Status |  |  |
| Single/Never Been Married | 0.22 | 855 |
| Married | 0.47 | 1797 |
| Separated | 0.04 | 158 |
| Divorced | 0.09 | 332 |
| Widowed | 0.06 | 215 |
| Domestic Partner | 0.12 | 450 |
| Missing | 0.01 | 38 |
| Employment |  |  |
| Employed for an Employer | 0.49 | 1881 |
| Self-Employed | 0.10 | 380 |
| Retired | 0.24 | 912 |
| Student | 0.05 | 190 |
| Homemaker | 0.04 | 137 |
| Unemployed and Looking for a Job | 0.03 | 134 |
| None of These/Other | 0.05 | 206 |
| Missing | 0.00 | 4 |
| Education |  |  |
| Up to 8 Years | 0.02 | 70 |
| 9-15 Years | 0.63 | 2434 |
| 16+ Years | 0.35 | 1330 |
| Missing | 0.00 | 10 |
| Service Attendance |  |  |
| >1/Week | 0.04 | 162 |
| 1/Week | 0.08 | 299 |
| 1-3/Month | 0.04 | 135 |
| A Few Times a Year | 0.17 | 656 |
| Never | 0.67 | 2584 |
| Missing | 0.00 | 7 |
| Immigration Status |  |  |
| Born in This Country | 0.77 | 2953 |
| Born in Another Country | 0.23 | 885 |
| Missing | 0.00 | 6 |
| Religion |  |  |
| Christianity | 0.41 | 1592 |
| Islam | 0.01 | 45 |
| Hinduism | 0.01 | 31 |
| Buddhism | 0.01 | 36 |
| Judaism | 0.01 | 26 |
| Sikhism | 0.00 | 8 |
| Baha'i | 0.00 | 7 |
| Jainism | . | . |
| Shinto | . | . |
| Taoism | 0.00 | 5 |
| Confucianism | . | . |
| Primal, Animist, or Folk Religion | 0.01 | 23 |
| Spiritism | . | . |
| African-Derived | . | . |
| Chinese | . | . |
| Some Other Religion | 0.01 | 39 |
| No Religion/Atheist/Agnostic | 0.53 | 2020 |
| Missing | 0.00 | 15 |
| Race/Ethnicity |  |  |
| Aboriginal | 0.01 | 53 |
| Australian | 0.51 | 1946 |
| Australian /British/European | 0.27 | 1047 |
| Chinese | 0.02 | 75 |
| Indian | 0.02 | 58 |
| Japanese | 0.00 | 1 |
| Malay | 0.00 | 11 |
| Sinhalese | 0.00 | 1 |
| Spanish | 0.00 | 2 |
| Sri Lankan Moor | 0.00 | 1 |
| Sri Lankan Tamil | 0.00 | 7 |
| Vietnamese | 0.00 | 7 |
| Taiwanese/Holo | . | . |
| Russian | 0.00 | 7 |
| Samoan | 0.00 | 4 |
| New Zealander | 0.02 | 91 |
| Other European | 0.09 | 357 |
| Other | 0.04 | 163 |
| Missing | 0.00 | 14 |

**Table S2b: Variations Across Demographic Characteristics (Australia)**

| Variable | Mean | SE | LCI | UCI | Global p-value |
| --- | --- | --- | --- | --- | --- |
| Age |  |  |  |  |  |
| 18-24 | 6.58 | 0.17 | 6.25 | 6.91 | 0.00 |
| 25-29 | 6.41 | 0.18 | 6.05 | 6.77 | . |
| 30-39 | 6.31 | 0.12 | 6.06 | 6.55 | . |
| 40-49 | 6.32 | 0.10 | 6.13 | 6.52 | . |
| 50-59 | 6.39 | 0.09 | 6.21 | 6.58 | . |
| 60-69 | 6.61 | 0.10 | 6.42 | 6.79 | . |
| 70-79 | 6.74 | 0.11 | 6.54 | 6.95 | . |
| 80 or Older | 7.01 | 0.15 | 6.72 | 7.30 | . |
| Gender |  |  |  |  |  |
| Male | 6.60 | 0.06 | 6.49 | 6.71 | 0.03 |
| Female | 6.40 | 0.07 | 6.27 | 6.52 | . |
| Other | 6.00 | 0.49 | 4.95 | 7.05 | . |
| Marital Status |  |  |  |  |  |
| Single/Never Been Married | 6.04 | 0.10 | 5.84 | 6.25 | 0.00 |
| Married | 6.77 | 0.06 | 6.66 | 6.88 | . |
| Separated | 5.92 | 0.24 | 5.46 | 6.39 | . |
| Divorced | 6.31 | 0.14 | 6.03 | 6.58 | . |
| Widowed | 6.38 | 0.18 | 6.03 | 6.73 | . |
| Domestic Partner | 6.63 | 0.13 | 6.36 | 6.89 | . |
| Employment |  |  |  |  |  |
| Employed for an Employer | 6.63 | 0.05 | 6.53 | 6.74 | 0.00 |
| Self-Employed | 7.01 | 0.12 | 6.78 | 7.25 | . |
| Retired | 6.60 | 0.08 | 6.44 | 6.76 | . |
| Student | 6.31 | 0.22 | 5.88 | 6.73 | . |
| Homemaker | 5.58 | 0.31 | 4.96 | 6.20 | . |
| Unemployed and Looking for a Job | 5.52 | 0.33 | 4.87 | 6.17 | . |
| None of These/Other | 5.17 | 0.23 | 4.72 | 5.62 | . |
| Education |  |  |  |  |  |
| Up to 8 Years | 6.97 | 0.40 | 6.14 | 7.81 | 0.00 |
| 9-15 Years | 6.32 | 0.06 | 6.20 | 6.43 | . |
| 16+ Years | 6.79 | 0.05 | 6.69 | 6.89 | . |
| Service Attendance |  |  |  |  |  |
| >1/Week | 7.08 | 0.19 | 6.70 | 7.46 | 0.00 |
| 1/Week | 6.61 | 0.15 | 6.31 | 6.90 | . |
| 1-3/Month | 6.70 | 0.18 | 6.34 | 7.07 | . |
| A Few Times a Year | 6.64 | 0.09 | 6.46 | 6.82 | . |
| Never | 6.39 | 0.05 | 6.29 | 6.50 | . |
| Immigration Status |  |  |  |  |  |
| Born in This Country | 6.42 | 0.05 | 6.32 | 6.51 | 0.00 |
| Born in Another Country | 6.75 | 0.08 | 6.59 | 6.91 | . |
| Religion |  |  |  |  |  |
| Christianity | 6.63 | 0.06 | 6.50 | 6.75 | 0.00 |
| Islam | 6.63 | 0.37 | 5.87 | 7.39 | . |
| Hinduism | 6.63 | 0.44 | 5.71 | 7.55 | . |
| Buddhism | 6.68 | 0.39 | 5.88 | 7.48 | . |
| Judaism | 6.89 | 0.30 | 6.28 | 7.50 | . |
| Sikhism | 6.17 | . | . | . | . |
| Baha'i | 8.20 | 0.36 | 5.08 | 11.32 | . |
| Jainism | . | . | . | . | . |
| Shinto | . | . | . | . | . |
| Taoism | 8.12 | . | . | . | . |
| Confucianism | . | . | . | . | . |
| Primal, Animist, or Folk Religion | 5.48 | 0.72 | 3.84 | 7.12 | . |
| Spiritism | . | . | . | . | . |
| African-Derived | . | . | . | . | . |
| Chinese | . | . | . | . | . |
| Some Other Religion | 5.66 | 0.73 | 4.13 | 7.18 | . |
| No Religion/Atheist/Agnostic | 6.39 | 0.06 | 6.27 | 6.51 | . |
| Race/Ethnicity |  |  |  |  |  |
| Aboriginal | 5.13 | 0.51 | 4.07 | 6.19 | 0.00 |
| Australian | 6.42 | 0.06 | 6.30 | 6.54 | . |
| Australian /British/European | 6.51 | 0.08 | 6.35 | 6.67 | . |
| Chinese | 7.00 | 0.24 | 6.51 | 7.48 | . |
| Indian | 6.79 | 0.31 | 6.16 | 7.42 | . |
| Japanese | . | . | . | . | . |
| Malay | 7.78 | 0.52 | 5.55 | 10.01 | . |
| Sinhalese | 9.27 | . | . | . | . |
| Spanish | 7.43 | . | . | . | . |
| Sri Lankan Moor | . | . | . | . | . |
| Sri Lankan Tamil | 6.08 | 1.91 | -0.19 | 12.34 | . |
| Vietnamese | 7.31 | 0.32 | 4.56 | 10.06 | . |
| Taiwanese/Holo | . | . | . | . | . |
| Russian | 6.58 | 0.50 | 4.91 | 8.25 | . |
| Samoan | 3.22 | . | . | . | . |
| New Zealander | 6.39 | 0.28 | 5.82 | 6.96 | . |
| Other European | 6.85 | 0.14 | 6.58 | 7.12 | . |
| Other | 6.53 | 0.20 | 6.12 | 6.93 | . |

**Table S3a: Nationally-Representative Descriptive Statistics of the Observed Sample (Brazil)**

| Variable | Proportion | Frequency |
| --- | --- | --- |
| Age |  |  |
| 18-24 | 0.15 | 1986 |
| 25-29 | 0.11 | 1468 |
| 30-39 | 0.22 | 2908 |
| 40-49 | 0.20 | 2638 |
| 50-59 | 0.16 | 2131 |
| 60-69 | 0.11 | 1435 |
| 70-79 | 0.04 | 510 |
| 80 or Older | 0.01 | 126 |
| Missing | . | . |
| Gender |  |  |
| Male | 0.48 | 6320 |
| Female | 0.52 | 6820 |
| Other | 0.00 | 35 |
| Missing | 0.00 | 30 |
| Marital Status |  |  |
| Single/Never Been Married | 0.33 | 4347 |
| Married | 0.35 | 4646 |
| Separated | 0.04 | 594 |
| Divorced | 0.07 | 865 |
| Widowed | 0.03 | 408 |
| Domestic Partner | 0.16 | 2081 |
| Missing | 0.02 | 263 |
| Employment |  |  |
| Employed for an Employer | 0.28 | 3756 |
| Self-Employed | 0.22 | 2918 |
| Retired | 0.12 | 1536 |
| Student | 0.05 | 624 |
| Homemaker | 0.10 | 1305 |
| Unemployed and Looking for a Job | 0.18 | 2419 |
| None of These/Other | 0.03 | 448 |
| Missing | 0.02 | 199 |
| Education |  |  |
| Up to 8 Years | 0.24 | 3139 |
| 9-15 Years | 0.58 | 7665 |
| 16+ Years | 0.18 | 2390 |
| Missing | 0.00 | 10 |
| Service Attendance |  |  |
| >1/Week | 0.18 | 2386 |
| 1/Week | 0.17 | 2272 |
| 1-3/Month | 0.11 | 1398 |
| A Few Times a Year | 0.30 | 3978 |
| Never | 0.24 | 3110 |
| Missing | 0.00 | 61 |
| Immigration Status |  |  |
| Born in This Country | 0.96 | 12688 |
| Born in Another Country | 0.01 | 153 |
| Missing | 0.03 | 363 |
| Religion |  |  |
| Christianity | 0.75 | 9911 |
| Islam | 0.00 | 6 |
| Hinduism | 0.00 | 1 |
| Buddhism | 0.00 | 37 |
| Judaism | 0.00 | 31 |
| Sikhism | . | . |
| Baha'i | 0.00 | 2 |
| Jainism | 0.00 | 2 |
| Shinto | 0.00 | 1 |
| Taoism | 0.00 | 2 |
| Confucianism | 0.00 | 6 |
| Primal, Animist, or Folk Religion | 0.00 | 15 |
| Spiritism | 0.05 | 696 |
| African-Derived | 0.04 | 525 |
| Chinese | . | . |
| Some Other Religion | 0.01 | 144 |
| No Religion/Atheist/Agnostic | 0.13 | 1712 |
| Missing | 0.01 | 113 |
| Race/Ethnicity |  |  |
| Branca | 0.39 | 5169 |
| Preta | 0.12 | 1615 |
| Parda | 0.39 | 5125 |
| Amarela | 0.02 | 238 |
| Indigena | 0.01 | 131 |
| Other | 0.00 | 61 |
| Missing | 0.07 | 865 |

**Table S3b: Variations Across Demographic Characteristics (Brazil)**

| Variable | Mean | SE | LCI | UCI | Global p-value |
| --- | --- | --- | --- | --- | --- |
| Age |  |  |  |  |  |
| 18-24 | 7.13 | 0.06 | 7.00 | 7.25 | 0.03 |
| 25-29 | 7.13 | 0.07 | 6.99 | 7.28 | . |
| 30-39 | 7.24 | 0.05 | 7.15 | 7.34 | . |
| 40-49 | 7.09 | 0.06 | 6.97 | 7.21 | . |
| 50-59 | 7.06 | 0.07 | 6.91 | 7.20 | . |
| 60-69 | 7.18 | 0.10 | 7.00 | 7.37 | . |
| 70-79 | 7.64 | 0.16 | 7.33 | 7.95 | . |
| 80 or Older | 7.01 | 0.30 | 6.39 | 7.62 | . |
| Gender |  |  |  |  |  |
| Male | 7.43 | 0.04 | 7.36 | 7.50 | 0.00 |
| Female | 6.91 | 0.04 | 6.84 | 6.99 | . |
| Other | 6.54 | 0.65 | 5.19 | 7.90 | . |
| Marital Status |  |  |  |  |  |
| Single/Never Been Married | 7.04 | 0.05 | 6.94 | 7.13 | 0.00 |
| Married | 7.40 | 0.04 | 7.32 | 7.48 | . |
| Separated | 6.99 | 0.13 | 6.73 | 7.24 | . |
| Divorced | 6.99 | 0.11 | 6.76 | 7.21 | . |
| Widowed | 7.19 | 0.21 | 6.79 | 7.60 | . |
| Domestic Partner | 7.00 | 0.06 | 6.88 | 7.12 | . |
| Employment |  |  |  |  |  |
| Employed for an Employer | 7.36 | 0.04 | 7.28 | 7.44 | 0.00 |
| Self-Employed | 7.34 | 0.05 | 7.24 | 7.44 | . |
| Retired | 7.27 | 0.09 | 7.09 | 7.44 | . |
| Student | 7.33 | 0.12 | 7.10 | 7.56 | . |
| Homemaker | 6.74 | 0.11 | 6.53 | 6.94 | . |
| Unemployed and Looking for a Job | 6.85 | 0.07 | 6.72 | 6.99 | . |
| None of These/Other | 6.58 | 0.17 | 6.24 | 6.92 | . |
| Education |  |  |  |  |  |
| Up to 8 Years | 7.20 | 0.07 | 7.06 | 7.33 | 0.06 |
| 9-15 Years | 7.11 | 0.03 | 7.05 | 7.18 | . |
| 16+ Years | 7.25 | 0.05 | 7.16 | 7.35 | . |
| Service Attendance |  |  |  |  |  |
| >1/Week | 7.61 | 0.07 | 7.48 | 7.74 | 0.00 |
| 1/Week | 7.36 | 0.06 | 7.24 | 7.49 | . |
| 1-3/Month | 7.17 | 0.08 | 7.02 | 7.32 | . |
| A Few Times a Year | 7.08 | 0.05 | 6.99 | 7.17 | . |
| Never | 6.76 | 0.06 | 6.65 | 6.88 | . |
| Immigration Status |  |  |  |  |  |
| Born in This Country | 7.16 | 0.03 | 7.10 | 7.21 | 0.61 |
| Born in Another Country | 7.29 | 0.26 | 6.78 | 7.80 | . |
| Religion |  |  |  |  |  |
| Christianity | 7.21 | 0.03 | 7.15 | 7.28 | 0.00 |
| Islam | 7.52 | 1.03 | 5.20 | 9.83 | . |
| Hinduism | 7.30 | 0.60 | 2.15 | 12.45 | . |
| Buddhism | 7.27 | 0.65 | 5.95 | 8.60 | . |
| Judaism | 6.74 | 0.61 | 5.48 | 8.01 | . |
| Sikhism | . | . | . | . | . |
| Baha'i | 10.00 | 0.00 | 10.00 | 10.00 | . |
| Jainism | 7.11 | 1.13 | 2.25 | 11.97 | . |
| Shinto | 7.00 | . | . | . | . |
| Taoism | 8.04 | 0.64 | 5.29 | 10.79 | . |
| Confucianism | 7.90 | 0.93 | 3.91 | 11.88 | . |
| Primal, Animist, or Folk Religion | 7.66 | 1.05 | 5.42 | 9.91 | . |
| Spiritism | 7.15 | 0.12 | 6.91 | 7.38 | . |
| African-Derived | 6.94 | 0.13 | 6.69 | 7.19 | . |
| Chinese | . | . | . | . | . |
| Some Other Religion | 7.35 | 0.26 | 6.84 | 7.86 | . |
| No Religion/Atheist/Agnostic | 6.89 | 0.07 | 6.75 | 7.03 | . |
| Race/Ethnicity |  |  |  |  |  |
| Branca | 7.14 | 0.04 | 7.06 | 7.23 | 0.56 |
| Preta | 7.11 | 0.08 | 6.96 | 7.27 | . |
| Parda | 7.21 | 0.04 | 7.13 | 7.29 | . |
| Amarela | 6.86 | 0.22 | 6.43 | 7.28 | . |
| Indigena | 7.11 | 0.31 | 6.50 | 7.73 | . |
| Other | 6.93 | 0.32 | 6.28 | 7.58 | . |

**Table S4a: Nationally-Representative Descriptive Statistics of the Observed Sample (Egypt)**

| Variable | Proportion | Frequency |
| --- | --- | --- |
| Age |  |  |
| 18-24 | 0.20 | 960 |
| 25-29 | 0.13 | 607 |
| 30-39 | 0.25 | 1204 |
| 40-49 | 0.19 | 897 |
| 50-59 | 0.13 | 613 |
| 60-69 | 0.08 | 387 |
| 70-79 | 0.01 | 54 |
| 80 or Older | 0.00 | 7 |
| Missing | . | . |
| Gender |  |  |
| Male | 0.51 | 2394 |
| Female | 0.49 | 2334 |
| Other | . | . |
| Missing | 0.00 | 0 |
| Marital Status |  |  |
| Single/Never Been Married | 0.20 | 947 |
| Married | 0.72 | 3387 |
| Separated | 0.01 | 39 |
| Divorced | 0.02 | 101 |
| Widowed | 0.05 | 238 |
| Domestic Partner | . | . |
| Missing | 0.00 | 17 |
| Employment |  |  |
| Employed for an Employer | 0.27 | 1267 |
| Self-Employed | 0.19 | 892 |
| Retired | 0.05 | 253 |
| Student | 0.06 | 297 |
| Homemaker | 0.37 | 1772 |
| Unemployed and Looking for a Job | 0.05 | 224 |
| None of These/Other | 0.00 | 21 |
| Missing | 0.00 | 3 |
| Education |  |  |
| Up to 8 Years | 0.53 | 2486 |
| 9-15 Years | 0.34 | 1599 |
| 16+ Years | 0.14 | 643 |
| Missing | 0.00 | 1 |
| Service Attendance |  |  |
| >1/Week | 0.18 | 839 |
| 1/Week | 0.20 | 960 |
| 1-3/Month | 0.08 | 368 |
| A Few Times a Year | 0.10 | 458 |
| Never | 0.44 | 2091 |
| Missing | 0.00 | 12 |
| Immigration Status |  |  |
| Born in This Country | 1.00 | 4713 |
| Born in Another Country | 0.00 | 16 |
| Missing | 0.00 | 1 |
| Religion |  |  |
| Christianity | 0.03 | 120 |
| Islam | 0.97 | 4607 |
| Hinduism | . | . |
| Buddhism | . | . |
| Judaism | . | . |
| Sikhism | . | . |
| Baha'i | . | . |
| Jainism | . | . |
| Shinto | . | . |
| Taoism | 0.00 | 0 |
| Confucianism | . | . |
| Primal, Animist, or Folk Religion | . | . |
| Spiritism | . | . |
| African-Derived | . | . |
| Chinese | . | . |
| Some Other Religion | . | . |
| No Religion/Atheist/Agnostic | . | . |
| Missing | 0.00 | 1 |
| Race/Ethnicity |  |  |
| Arab | 0.97 | 4585 |
| Turkish | 0.00 | 9 |
| Greek | 0.00 | 1 |
| Abazas | . | . |
| Bedouin Arab | 0.00 | 4 |
| Swiss | . | . |
| Nubian | 0.01 | 27 |
| Other | . | . |
| Missing | 0.02 | 102 |

**Table S4b: Variations Across Demographic Characteristics (Egypt)**

| Variable | Mean | SE | LCI | UCI | Global p-value |
| --- | --- | --- | --- | --- | --- |
| Age |  |  |  |  |  |
| 18-24 | 7.73 | 0.11 | 7.51 | 7.95 | 0.00 |
| 25-29 | 7.07 | 0.14 | 6.78 | 7.35 | . |
| 30-39 | 6.92 | 0.09 | 6.73 | 7.10 | . |
| 40-49 | 6.48 | 0.11 | 6.27 | 6.69 | . |
| 50-59 | 6.06 | 0.11 | 5.83 | 6.29 | . |
| 60-69 | 6.04 | 0.20 | 5.65 | 6.43 | . |
| 70-79 | 4.94 | 0.41 | 4.08 | 5.80 | . |
| 80 or Older | . | . | . | . | . |
| Gender |  |  |  |  |  |
| Male | 7.11 | 0.08 | 6.95 | 7.27 | 0.00 |
| Female | 6.50 | 0.06 | 6.38 | 6.62 | . |
| Other | . | . | . | . | . |
| Marital Status |  |  |  |  |  |
| Single/Never Been Married | 7.81 | 0.10 | 7.61 | 8.01 | 0.00 |
| Married | 6.63 | 0.06 | 6.50 | 6.76 | . |
| Separated | 5.81 | 0.44 | 4.86 | 6.76 | . |
| Divorced | 5.72 | 0.32 | 5.08 | 6.36 | . |
| Widowed | 6.01 | 0.22 | 5.58 | 6.44 | . |
| Domestic Partner | . | . | . | . | . |
| Employment |  |  |  |  |  |
| Employed for an Employer | 7.08 | 0.10 | 6.88 | 7.29 | 0.00 |
| Self-Employed | 7.06 | 0.12 | 6.83 | 7.29 | . |
| Retired | 5.71 | 0.26 | 5.20 | 6.22 | . |
| Student | 8.08 | 0.14 | 7.80 | 8.36 | . |
| Homemaker | 6.40 | 0.07 | 6.26 | 6.54 | . |
| Unemployed and Looking for a Job | 7.06 | 0.21 | 6.64 | 7.48 | . |
| None of These/Other | 7.10 | 0.52 | 5.38 | 8.81 | . |
| Education |  |  |  |  |  |
| Up to 8 Years | 6.36 | 0.08 | 6.20 | 6.52 | 0.00 |
| 9-15 Years | 7.24 | 0.06 | 7.12 | 7.36 | . |
| 16+ Years | 7.48 | 0.13 | 7.22 | 7.74 | . |
| Service Attendance |  |  |  |  |  |
| >1/Week | 7.07 | 0.11 | 6.86 | 7.29 | 0.00 |
| 1/Week | 7.03 | 0.12 | 6.79 | 7.27 | . |
| 1-3/Month | 6.73 | 0.20 | 6.33 | 7.13 | . |
| A Few Times a Year | 7.14 | 0.15 | 6.85 | 7.43 | . |
| Never | 6.55 | 0.08 | 6.40 | 6.70 | . |
| Immigration Status |  |  |  |  |  |
| Born in This Country | 6.81 | 0.05 | 6.71 | 6.92 | 0.55 |
| Born in Another Country | 6.40 | 0.51 | 4.95 | 7.85 | . |
| Religion |  |  |  |  |  |
| Christianity | 6.19 | 0.27 | 5.63 | 6.75 | 0.06 |
| Islam | 6.83 | 0.05 | 6.72 | 6.93 | . |
| Hinduism | . | . | . | . | . |
| Buddhism | . | . | . | . | . |
| Judaism | . | . | . | . | . |
| Sikhism | . | . | . | . | . |
| Baha'i | . | . | . | . | . |
| Jainism | . | . | . | . | . |
| Shinto | . | . | . | . | . |
| Taoism | 5.00 | . | . | . | . |
| Confucianism | . | . | . | . | . |
| Primal, Animist, or Folk Religion | . | . | . | . | . |
| Spiritism | . | . | . | . | . |
| African-Derived | . | . | . | . | . |
| Chinese | . | . | . | . | . |
| Some Other Religion | . | . | . | . | . |
| No Religion/Atheist/Agnostic | . | . | . | . | . |
| Race/Ethnicity |  |  |  |  |  |
| Arab | 6.81 | 0.05 | 6.70 | 6.91 | 0.00 |
| Turkish | 7.53 | 0.90 | 4.59 | 10.46 | . |
| Greek | 7.00 | . | . | . | . |
| Abazas | . | . | . | . | . |
| Bedouin Arab | 6.86 | . | . | . | . |
| Swiss | . | . | . | . | . |
| Nubian | 7.35 | . | . | . | . |
| Other | . | . | . | . | . |

**Table S5a: Nationally-Representative Descriptive Statistics of the Observed Sample (Germany)**

| Variable | Proportion | Frequency |
| --- | --- | --- |
| Age |  |  |
| 18-24 | 0.09 | 829 |
| 25-29 | 0.08 | 774 |
| 30-39 | 0.15 | 1438 |
| 40-49 | 0.16 | 1494 |
| 50-59 | 0.18 | 1729 |
| 60-69 | 0.20 | 1915 |
| 70-79 | 0.12 | 1137 |
| 80 or Older | 0.02 | 190 |
| Missing | . | . |
| Gender |  |  |
| Male | 0.49 | 4641 |
| Female | 0.51 | 4843 |
| Other | 0.00 | 11 |
| Missing | 0.00 | 11 |
| Marital Status |  |  |
| Single/Never Been Married | 0.28 | 2627 |
| Married | 0.50 | 4784 |
| Separated | 0.02 | 219 |
| Divorced | 0.08 | 767 |
| Widowed | 0.04 | 409 |
| Domestic Partner | 0.07 | 619 |
| Missing | 0.01 | 81 |
| Employment |  |  |
| Employed for an Employer | 0.52 | 4950 |
| Self-Employed | 0.07 | 712 |
| Retired | 0.26 | 2480 |
| Student | 0.06 | 605 |
| Homemaker | 0.03 | 251 |
| Unemployed and Looking for a Job | 0.03 | 288 |
| None of These/Other | 0.02 | 204 |
| Missing | 0.00 | 14 |
| Education |  |  |
| Up to 8 Years | 0.02 | 235 |
| 9-15 Years | 0.64 | 6094 |
| 16+ Years | 0.33 | 3164 |
| Missing | 0.00 | 13 |
| Service Attendance |  |  |
| >1/Week | 0.03 | 285 |
| 1/Week | 0.04 | 424 |
| 1-3/Month | 0.06 | 550 |
| A Few Times a Year | 0.25 | 2362 |
| Never | 0.62 | 5876 |
| Missing | 0.00 | 9 |
| Immigration Status |  |  |
| Born in This Country | 0.92 | 8722 |
| Born in Another Country | 0.08 | 744 |
| Missing | 0.00 | 40 |
| Religion |  |  |
| Christianity | 0.53 | 5052 |
| Islam | 0.04 | 351 |
| Hinduism | 0.00 | 12 |
| Buddhism | 0.01 | 51 |
| Judaism | 0.00 | 19 |
| Sikhism | 0.00 | 5 |
| Baha'i | 0.00 | 3 |
| Jainism | . | . |
| Shinto | 0.00 | 2 |
| Taoism | 0.00 | 0 |
| Confucianism | 0.00 | 4 |
| Primal, Animist, or Folk Religion | 0.00 | 34 |
| Spiritism | . | . |
| African-Derived | . | . |
| Chinese | . | . |
| Some Other Religion | 0.01 | 60 |
| No Religion/Atheist/Agnostic | 0.40 | 3815 |
| Missing | 0.01 | 99 |
| Race/Ethnicity |  |  |
| No Data | . | . |

**Table S5b: Variations Across Demographic Characteristics (Germany)**

| Variable | Mean | SE | LCI | UCI | Global p-value |
| --- | --- | --- | --- | --- | --- |
| Age |  |  |  |  |  |
| 18-24 | 7.04 | 0.09 | 6.86 | 7.21 | 0.00 |
| 25-29 | 7.19 | 0.08 | 7.03 | 7.35 | . |
| 30-39 | 7.01 | 0.06 | 6.90 | 7.13 | . |
| 40-49 | 6.70 | 0.07 | 6.56 | 6.84 | . |
| 50-59 | 6.45 | 0.07 | 6.32 | 6.58 | . |
| 60-69 | 6.27 | 0.06 | 6.15 | 6.39 | . |
| 70-79 | 6.24 | 0.09 | 6.06 | 6.42 | . |
| 80 or Older | 6.29 | 0.20 | 5.89 | 6.68 | . |
| Gender |  |  |  |  |  |
| Male | 6.69 | 0.04 | 6.61 | 6.76 | 0.01 |
| Female | 6.56 | 0.04 | 6.48 | 6.64 | . |
| Other | 5.62 | 0.48 | 4.58 | 6.66 | . |
| Marital Status |  |  |  |  |  |
| Single/Never Been Married | 6.68 | 0.05 | 6.57 | 6.78 | 0.00 |
| Married | 6.67 | 0.04 | 6.60 | 6.75 | . |
| Separated | 6.53 | 0.14 | 6.25 | 6.81 | . |
| Divorced | 6.07 | 0.10 | 5.87 | 6.27 | . |
| Widowed | 6.24 | 0.15 | 5.94 | 6.53 | . |
| Domestic Partner | 6.95 | 0.10 | 6.76 | 7.14 | . |
| Employment |  |  |  |  |  |
| Employed for an Employer | 6.86 | 0.03 | 6.79 | 6.93 | 0.00 |
| Self-Employed | 7.06 | 0.09 | 6.88 | 7.24 | . |
| Retired | 6.05 | 0.06 | 5.94 | 6.17 | . |
| Student | 7.32 | 0.10 | 7.13 | 7.50 | . |
| Homemaker | 6.00 | 0.18 | 5.65 | 6.35 | . |
| Unemployed and Looking for a Job | 5.77 | 0.19 | 5.41 | 6.14 | . |
| None of These/Other | 6.09 | 0.19 | 5.71 | 6.46 | . |
| Education |  |  |  |  |  |
| Up to 8 Years | 6.17 | 0.18 | 5.83 | 6.52 | 0.00 |
| 9-15 Years | 6.41 | 0.03 | 6.34 | 6.48 | . |
| 16+ Years | 7.07 | 0.04 | 6.98 | 7.15 | . |
| Service Attendance |  |  |  |  |  |
| >1/Week | 7.05 | 0.16 | 6.73 | 7.36 | 0.00 |
| 1/Week | 7.20 | 0.11 | 6.98 | 7.43 | . |
| 1-3/Month | 7.02 | 0.11 | 6.81 | 7.23 | . |
| A Few Times a Year | 6.72 | 0.05 | 6.62 | 6.83 | . |
| Never | 6.48 | 0.04 | 6.41 | 6.55 | . |
| Immigration Status |  |  |  |  |  |
| Born in This Country | 6.59 | 0.03 | 6.53 | 6.64 | 0.00 |
| Born in Another Country | 7.03 | 0.09 | 6.85 | 7.22 | . |
| Religion |  |  |  |  |  |
| Christianity | 6.60 | 0.04 | 6.53 | 6.67 | 0.00 |
| Islam | 6.76 | 0.17 | 6.42 | 7.10 | . |
| Hinduism | 7.00 | 0.73 | 3.84 | 10.16 | . |
| Buddhism | 6.84 | 0.24 | 6.35 | 7.33 | . |
| Judaism | 6.42 | 0.64 | 4.80 | 8.05 | . |
| Sikhism | . | . | . | . | . |
| Baha'i | 9.71 | . | . | . | . |
| Jainism | . | . | . | . | . |
| Shinto | 6.00 | . | . | . | . |
| Taoism | 9.00 | . | . | . | . |
| Confucianism | 8.77 | . | . | . | . |
| Primal, Animist, or Folk Religion | 6.32 | 0.36 | 5.56 | 7.08 | . |
| Spiritism | . | . | . | . | . |
| African-Derived | . | . | . | . | . |
| Chinese | . | . | . | . | . |
| Some Other Religion | 5.68 | 0.34 | 4.99 | 6.37 | . |
| No Religion/Atheist/Agnostic | 6.65 | 0.04 | 6.56 | 6.74 | . |
| Race/Ethnicity |  |  |  |  |  |
| No Data | . | . | . | . | . |

**Table S6a: Nationally-Representative Descriptive Statistics of the Observed Sample (Hong Kong)**

| Variable | Proportion | Frequency |
| --- | --- | --- |
| Age |  |  |
| 18-24 | 0.07 | 217 |
| 25-29 | 0.07 | 198 |
| 30-39 | 0.17 | 507 |
| 40-49 | 0.19 | 580 |
| 50-59 | 0.24 | 711 |
| 60-69 | 0.21 | 620 |
| 70-79 | 0.05 | 164 |
| 80 or Older | 0.00 | 15 |
| Missing | . | . |
| Gender |  |  |
| Male | 0.46 | 1390 |
| Female | 0.54 | 1620 |
| Other | 0.00 | 2 |
| Missing | . | . |
| Marital Status |  |  |
| Single/Never Been Married | 0.24 | 723 |
| Married | 0.69 | 2080 |
| Separated | 0.01 | 21 |
| Divorced | 0.03 | 105 |
| Widowed | 0.01 | 45 |
| Domestic Partner | 0.01 | 37 |
| Missing | 0.00 | 1 |
| Employment |  |  |
| Employed for an Employer | 0.68 | 2056 |
| Self-Employed | 0.08 | 245 |
| Retired | 0.14 | 423 |
| Student | 0.02 | 55 |
| Homemaker | 0.04 | 114 |
| Unemployed and Looking for a Job | 0.02 | 62 |
| None of These/Other | 0.01 | 39 |
| Missing | 0.01 | 18 |
| Education |  |  |
| Up to 8 Years | 0.14 | 433 |
| 9-15 Years | 0.67 | 2031 |
| 16+ Years | 0.18 | 547 |
| Missing | . | . |
| Service Attendance |  |  |
| >1/Week | 0.08 | 237 |
| 1/Week | 0.19 | 567 |
| 1-3/Month | 0.11 | 332 |
| A Few Times a Year | 0.18 | 543 |
| Never | 0.44 | 1332 |
| Missing | 0.00 | 1 |
| Immigration Status |  |  |
| Born in This Country | 0.88 | 2637 |
| Born in Another Country | 0.11 | 321 |
| Missing | 0.02 | 53 |
| Religion |  |  |
| Christianity | 0.25 | 757 |
| Islam | 0.03 | 86 |
| Hinduism | 0.01 | 20 |
| Buddhism | 0.12 | 349 |
| Judaism | 0.00 | 10 |
| Sikhism | 0.00 | 2 |
| Baha'i | 0.00 | 3 |
| Jainism | 0.00 | 0 |
| Shinto | 0.01 | 19 |
| Taoism | 0.03 | 97 |
| Confucianism | 0.00 | 11 |
| Primal, Animist, or Folk Religion | 0.01 | 27 |
| Spiritism | . | . |
| African-Derived | . | . |
| Chinese | 0.04 | 106 |
| Some Other Religion | 0.00 | 4 |
| No Religion/Atheist/Agnostic | 0.50 | 1518 |
| Missing | 0.00 | 5 |
| Race/Ethnicity |  |  |
| Chinese (Cantonese) | 0.64 | 1930 |
| Chinese (Chaoshan) | 0.07 | 201 |
| Chinese (Fujianese) | 0.04 | 117 |
| Chinese (Hakka) | 0.04 | 121 |
| Chinese (Shanghainese) | 0.03 | 89 |
| Chinese (Other Ethnicity) | 0.09 | 264 |
| East Asian (Korean, Japanese) | 0.00 | 10 |
| Southeast Asian (Filipino, Indonesian, Thailand) | 0.02 | 46 |
| South Asian (Indian, Nepalese, Pakistani) | 0.01 | 17 |
| Taiwanese | 0.00 | 14 |
| White | 0.00 | 15 |
| Other | 0.00 | 4 |
| Missing | 0.06 | 184 |

**Table S6b: Variations Across Demographic Characteristics (Hong Kong)**

| Variable | Mean | SE | LCI | UCI | Global p-value |
| --- | --- | --- | --- | --- | --- |
| Age |  |  |  |  |  |
| 18-24 | 7.43 | 0.11 | 7.21 | 7.65 | 0.00 |
| 25-29 | 6.90 | 0.21 | 6.47 | 7.32 | . |
| 30-39 | 6.66 | 0.11 | 6.45 | 6.87 | . |
| 40-49 | 7.09 | 0.10 | 6.90 | 7.28 | . |
| 50-59 | 7.50 | 0.08 | 7.34 | 7.66 | . |
| 60-69 | 7.26 | 0.13 | 6.99 | 7.52 | . |
| 70-79 | 6.48 | 0.35 | 5.76 | 7.19 | . |
| 80 or Older | 6.41 | 0.62 | 1.01 | 11.81 | . |
| Gender |  |  |  |  |  |
| Male | 7.23 | 0.07 | 7.09 | 7.38 | 0.05 |
| Female | 7.03 | 0.07 | 6.90 | 7.16 | . |
| Other | . | . | . | . | . |
| Marital Status |  |  |  |  |  |
| Single/Never Been Married | 6.33 | 0.09 | 6.16 | 6.51 | 0.00 |
| Married | 7.46 | 0.06 | 7.35 | 7.58 | . |
| Separated | 5.43 | 1.53 | 1.61 | 9.24 | . |
| Divorced | 6.84 | 0.33 | 6.19 | 7.49 | . |
| Widowed | 5.80 | 0.39 | 4.99 | 6.61 | . |
| Domestic Partner | 6.77 | 0.40 | 5.96 | 7.57 | . |
| Employment |  |  |  |  |  |
| Employed for an Employer | 7.29 | 0.05 | 7.20 | 7.39 | 0.00 |
| Self-Employed | 7.50 | 0.22 | 7.06 | 7.94 | . |
| Retired | 6.59 | 0.18 | 6.24 | 6.95 | . |
| Student | 6.73 | 0.26 | 6.20 | 7.25 | . |
| Homemaker | 6.56 | 0.26 | 6.04 | 7.08 | . |
| Unemployed and Looking for a Job | 6.07 | 0.26 | 5.54 | 6.60 | . |
| None of These/Other | 5.33 | 0.88 | 3.53 | 7.14 | . |
| Education |  |  |  |  |  |
| Up to 8 Years | 7.31 | 0.20 | 6.92 | 7.70 | 0.00 |
| 9-15 Years | 7.17 | 0.06 | 7.06 | 7.28 | . |
| 16+ Years | 6.81 | 0.08 | 6.64 | 6.97 | . |
| Service Attendance |  |  |  |  |  |
| >1/Week | 9.18 | 0.10 | 8.98 | 9.37 | 0.00 |
| 1/Week | 7.65 | 0.11 | 7.42 | 7.87 | . |
| 1-3/Month | 7.59 | 0.12 | 7.36 | 7.82 | . |
| A Few Times a Year | 6.81 | 0.12 | 6.57 | 7.05 | . |
| Never | 6.55 | 0.07 | 6.41 | 6.69 | . |
| Immigration Status |  |  |  |  |  |
| Born in This Country | 7.21 | 0.05 | 7.12 | 7.30 | 0.00 |
| Born in Another Country | 6.42 | 0.22 | 5.99 | 6.86 | . |
| Religion |  |  |  |  |  |
| Christianity | 7.23 | 0.10 | 7.03 | 7.42 | 0.00 |
| Islam | 7.91 | 0.53 | 6.86 | 8.96 | . |
| Hinduism | 8.02 | 0.63 | 6.67 | 9.36 | . |
| Buddhism | 7.85 | 0.15 | 7.56 | 8.14 | . |
| Judaism | 8.71 | 0.35 | 7.93 | 9.49 | . |
| Sikhism | . | . | . | . | . |
| Baha'i | 8.04 | 0.68 | 5.83 | 10.26 | . |
| Jainism | 9.00 | . | . | . | . |
| Shinto | 8.46 | 0.24 | 7.89 | 9.02 | . |
| Taoism | 7.17 | 0.27 | 6.63 | 7.72 | . |
| Confucianism | 7.19 | 0.41 | 6.29 | 8.09 | . |
| Primal, Animist, or Folk Religion | 6.87 | 0.24 | 6.39 | 7.35 | . |
| Spiritism | . | . | . | . | . |
| African-Derived | . | . | . | . | . |
| Chinese | 7.65 | 0.20 | 7.25 | 8.05 | . |
| Some Other Religion | 4.59 | 3.12 | -22.39 | 31.58 | . |
| No Religion/Atheist/Agnostic | 6.79 | 0.06 | 6.66 | 6.92 | . |
| Race/Ethnicity |  |  |  |  |  |
| Chinese (Cantonese) | 7.05 | 0.06 | 6.93 | 7.17 | 0.27 |
| Chinese (Chaoshan) | 7.21 | 0.22 | 6.79 | 7.63 | . |
| Chinese (Fujianese) | 7.35 | 0.19 | 6.96 | 7.73 | . |
| Chinese (Hakka) | 6.89 | 0.22 | 6.45 | 7.32 | . |
| Chinese (Shanghainese) | 7.50 | 0.32 | 6.86 | 8.15 | . |
| Chinese (Other Ethnicity) | 7.28 | 0.16 | 6.96 | 7.60 | . |
| East Asian (Korean, Japanese) | 8.42 | 0.68 | 6.23 | 10.61 | . |
| Southeast Asian (Filipino, Indonesian, Thailand) | 8.10 | 0.69 | 6.67 | 9.53 | . |
| South Asian (Indian, Nepalese, Pakistani) | 6.54 | 0.71 | 4.84 | 8.24 | . |
| Taiwanese | 7.70 | 1.24 | 4.15 | 11.25 | . |
| White | 7.00 | 0.54 | 5.80 | 8.21 | . |
| Other | 7.07 | 0.42 | 5.68 | 8.46 | . |

**Table S7a: Nationally-Representative Descriptive Statistics of the Observed Sample (India)**

| Variable | Proportion | Frequency |
| --- | --- | --- |
| Age |  |  |
| 18-24 | 0.20 | 2543 |
| 25-29 | 0.13 | 1640 |
| 30-39 | 0.24 | 3109 |
| 40-49 | 0.18 | 2275 |
| 50-59 | 0.12 | 1574 |
| 60-69 | 0.09 | 1188 |
| 70-79 | 0.03 | 370 |
| 80 or Older | 0.01 | 67 |
| Missing | . | . |
| Gender |  |  |
| Male | 0.51 | 6473 |
| Female | 0.49 | 6292 |
| Other | . | . |
| Missing | . | . |
| Marital Status |  |  |
| Single/Never Been Married | 0.16 | 2065 |
| Married | 0.77 | 9848 |
| Separated | 0.00 | 45 |
| Divorced | 0.00 | 25 |
| Widowed | 0.03 | 445 |
| Domestic Partner | 0.02 | 269 |
| Missing | 0.01 | 69 |
| Employment |  |  |
| Employed for an Employer | 0.21 | 2660 |
| Self-Employed | 0.27 | 3401 |
| Retired | 0.02 | 286 |
| Student | 0.04 | 532 |
| Homemaker | 0.33 | 4221 |
| Unemployed and Looking for a Job | 0.07 | 902 |
| None of These/Other | 0.06 | 715 |
| Missing | 0.00 | 48 |
| Education |  |  |
| Up to 8 Years | 0.89 | 11422 |
| 9-15 Years | 0.09 | 1194 |
| 16+ Years | 0.01 | 145 |
| Missing | 0.00 | 4 |
| Service Attendance |  |  |
| >1/Week | 0.23 | 2875 |
| 1/Week | 0.25 | 3166 |
| 1-3/Month | 0.21 | 2740 |
| A Few Times a Year | 0.16 | 2090 |
| Never | 0.14 | 1823 |
| Missing | 0.01 | 71 |
| Immigration Status |  |  |
| Born in This Country | 0.99 | 12629 |
| Born in Another Country | 0.01 | 110 |
| Missing | 0.00 | 26 |
| Religion |  |  |
| Christianity | 0.02 | 306 |
| Islam | 0.12 | 1555 |
| Hinduism | 0.81 | 10362 |
| Buddhism | 0.02 | 230 |
| Judaism | . | . |
| Sikhism | 0.01 | 127 |
| Baha'i | . | . |
| Jainism | 0.00 | 10 |
| Shinto | 0.00 | 1 |
| Taoism | . | . |
| Confucianism | . | . |
| Primal, Animist, or Folk Religion | 0.00 | 30 |
| Spiritism | . | . |
| African-Derived | . | . |
| Chinese | . | . |
| Some Other Religion | 0.01 | 67 |
| No Religion/Atheist/Agnostic | 0.00 | 13 |
| Missing | 0.00 | 62 |
| Race/Ethnicity |  |  |
| General | 0.28 | 3538 |
| Other Backward Caste | 0.33 | 4177 |
| Schedule Caste | 0.28 | 3599 |
| Schedule Tribe | 0.09 | 1185 |
| Other | . | . |
| Missing | 0.02 | 267 |

**Table S7b: Variations Across Demographic Characteristics (India)**

| Variable | Mean | SE | LCI | UCI | Global p-value |
| --- | --- | --- | --- | --- | --- |
| Age |  |  |  |  |  |
| 18-24 | 7.94 | 0.09 | 7.77 | 8.10 | 0.00 |
| 25-29 | 7.52 | 0.09 | 7.35 | 7.69 | . |
| 30-39 | 7.14 | 0.07 | 7.01 | 7.27 | . |
| 40-49 | 6.85 | 0.08 | 6.68 | 7.01 | . |
| 50-59 | 6.20 | 0.12 | 5.97 | 6.43 | . |
| 60-69 | 5.69 | 0.14 | 5.42 | 5.97 | . |
| 70-79 | 5.77 | 0.25 | 5.27 | 6.26 | . |
| 80 or Older | 6.42 | 0.44 | 5.51 | 7.32 | . |
| Gender |  |  |  |  |  |
| Male | 7.19 | 0.05 | 7.08 | 7.30 | 0.00 |
| Female | 6.81 | 0.06 | 6.69 | 6.92 | . |
| Other | . | . | . | . | . |
| Marital Status |  |  |  |  |  |
| Single/Never Been Married | 7.94 | 0.09 | 7.77 | 8.11 | 0.00 |
| Married | 6.89 | 0.05 | 6.80 | 6.98 | . |
| Separated | 5.88 | 0.49 | 4.84 | 6.92 | . |
| Divorced | 7.49 | 0.33 | 6.67 | 8.32 | . |
| Widowed | 5.55 | 0.21 | 5.13 | 5.96 | . |
| Domestic Partner | 6.58 | 0.29 | 5.99 | 7.16 | . |
| Employment |  |  |  |  |  |
| Employed for an Employer | 6.99 | 0.08 | 6.82 | 7.15 | 0.00 |
| Self-Employed | 7.21 | 0.08 | 7.05 | 7.37 | . |
| Retired | 6.41 | 0.26 | 5.89 | 6.93 | . |
| Student | 8.05 | 0.15 | 7.76 | 8.34 | . |
| Homemaker | 6.79 | 0.07 | 6.65 | 6.93 | . |
| Unemployed and Looking for a Job | 7.05 | 0.15 | 6.77 | 7.34 | . |
| None of These/Other | 6.74 | 0.15 | 6.45 | 7.04 | . |
| Education |  |  |  |  |  |
| Up to 8 Years | 6.91 | 0.05 | 6.83 | 7.00 | 0.00 |
| 9-15 Years | 7.74 | 0.09 | 7.57 | 7.90 | . |
| 16+ Years | 7.86 | 0.17 | 7.51 | 8.21 | . |
| Service Attendance |  |  |  |  |  |
| >1/Week | 6.91 | 0.08 | 6.74 | 7.07 | 0.07 |
| 1/Week | 7.19 | 0.08 | 7.04 | 7.34 | . |
| 1-3/Month | 6.93 | 0.08 | 6.77 | 7.10 | . |
| A Few Times a Year | 6.98 | 0.09 | 6.81 | 7.16 | . |
| Never | 6.95 | 0.11 | 6.74 | 7.16 | . |
| Immigration Status |  |  |  |  |  |
| Born in This Country | 7.01 | 0.04 | 6.93 | 7.09 | 0.01 |
| Born in Another Country | 5.89 | 0.42 | 5.03 | 6.74 | . |
| Religion |  |  |  |  |  |
| Christianity | 6.91 | 0.22 | 6.47 | 7.35 | 0.00 |
| Islam | 6.85 | 0.13 | 6.60 | 7.10 | . |
| Hinduism | 7.02 | 0.05 | 6.93 | 7.12 | . |
| Buddhism | 7.31 | 0.22 | 6.87 | 7.75 | . |
| Judaism | . | . | . | . | . |
| Sikhism | 6.59 | 0.48 | 5.51 | 7.68 | . |
| Baha'i | . | . | . | . | . |
| Jainism | . | . | . | . | . |
| Shinto | 10.00 | . | . | . | . |
| Taoism | . | . | . | . | . |
| Confucianism | . | . | . | . | . |
| Primal, Animist, or Folk Religion | 7.06 | 0.55 | 5.74 | 8.38 | . |
| Spiritism | . | . | . | . | . |
| African-Derived | . | . | . | . | . |
| Chinese | . | . | . | . | . |
| Some Other Religion | 7.69 | 0.81 | 5.68 | 9.69 | . |
| No Religion/Atheist/Agnostic | 5.39 | . | . | . | . |
| Race/Ethnicity |  |  |  |  |  |
| General | 6.91 | 0.08 | 6.76 | 7.07 | 0.40 |
| Other Backward Caste | 7.04 | 0.07 | 6.91 | 7.17 | . |
| Schedule Caste | 6.99 | 0.08 | 6.84 | 7.15 | . |
| Schedule Tribe | 7.15 | 0.12 | 6.91 | 7.39 | . |
| Other | . | . | . | . | . |

**Table S8a: Nationally-Representative Descriptive Statistics of the Observed Sample (Indonesia)**

| Variable | Proportion | Frequency |
| --- | --- | --- |
| Age |  |  |
| 18-24 | 0.17 | 1216 |
| 25-29 | 0.12 | 849 |
| 30-39 | 0.23 | 1591 |
| 40-49 | 0.23 | 1576 |
| 50-59 | 0.17 | 1169 |
| 60-69 | 0.07 | 490 |
| 70-79 | 0.01 | 83 |
| 80 or Older | 0.00 | 17 |
| Missing | . | . |
| Gender |  |  |
| Male | 0.50 | 3461 |
| Female | 0.50 | 3513 |
| Other | 0.00 | 7 |
| Missing | 0.00 | 11 |
| Marital Status |  |  |
| Single/Never Been Married | 0.20 | 1381 |
| Married | 0.69 | 4846 |
| Separated | 0.01 | 82 |
| Divorced | 0.03 | 196 |
| Widowed | 0.06 | 425 |
| Domestic Partner | 0.00 | 18 |
| Missing | 0.01 | 45 |
| Employment |  |  |
| Employed for an Employer | 0.19 | 1323 |
| Self-Employed | 0.31 | 2187 |
| Retired | 0.01 | 78 |
| Student | 0.04 | 272 |
| Homemaker | 0.31 | 2138 |
| Unemployed and Looking for a Job | 0.08 | 529 |
| None of These/Other | 0.06 | 448 |
| Missing | 0.00 | 18 |
| Education |  |  |
| Up to 8 Years | 0.44 | 3079 |
| 9-15 Years | 0.50 | 3491 |
| 16+ Years | 0.06 | 419 |
| Missing | 0.00 | 2 |
| Service Attendance |  |  |
| >1/Week | 0.38 | 2667 |
| 1/Week | 0.36 | 2529 |
| 1-3/Month | 0.11 | 786 |
| A Few Times a Year | 0.09 | 659 |
| Never | 0.05 | 332 |
| Missing | 0.00 | 18 |
| Immigration Status |  |  |
| Born in This Country | 1.00 | 6958 |
| Born in Another Country | 0.00 | 34 |
| Missing | . | . |
| Religion |  |  |
| Christianity | 0.07 | 504 |
| Islam | 0.92 | 6406 |
| Hinduism | 0.01 | 73 |
| Buddhism | 0.00 | 3 |
| Judaism | . | . |
| Sikhism | . | . |
| Baha'i | . | . |
| Jainism | . | . |
| Shinto | . | . |
| Taoism | 0.00 | 1 |
| Confucianism | . | . |
| Primal, Animist, or Folk Religion | . | . |
| Spiritism | . | . |
| African-Derived | . | . |
| Chinese | . | . |
| Some Other Religion | 0.00 | 1 |
| No Religion/Atheist/Agnostic | . | . |
| Missing | 0.00 | 4 |
| Race/Ethnicity |  |  |
| Banjar/Melayu Banjar | 0.05 | 320 |
| Betawi | 0.04 | 251 |
| Bugis | 0.03 | 243 |
| Jawa | 0.41 | 2846 |
| Madura | 0.04 | 262 |
| Minangkabau | 0.04 | 273 |
| Sunda/Parahyangan | 0.17 | 1172 |
| Bali | 0.01 | 69 |
| Batak | 0.02 | 165 |
| Makasar | 0.01 | 91 |
| Other | 0.18 | 1262 |
| Missing | 0.01 | 38 |

**Table S8b: Variations Across Demographic Characteristics (Indonesia)**

| Variable | Mean | SE | LCI | UCI | Global p-value |
| --- | --- | --- | --- | --- | --- |
| Age |  |  |  |  |  |
| 18-24 | 8.32 | 0.07 | 8.19 | 8.45 | 0.00 |
| 25-29 | 8.58 | 0.07 | 8.43 | 8.73 | . |
| 30-39 | 8.53 | 0.05 | 8.43 | 8.64 | . |
| 40-49 | 8.32 | 0.07 | 8.19 | 8.45 | . |
| 50-59 | 7.93 | 0.09 | 7.74 | 8.11 | . |
| 60-69 | 7.83 | 0.16 | 7.51 | 8.15 | . |
| 70-79 | 7.70 | 0.33 | 7.01 | 8.39 | . |
| 80 or Older | . | . | . | . | . |
| Gender |  |  |  |  |  |
| Male | 8.28 | 0.05 | 8.18 | 8.38 | 0.06 |
| Female | 8.30 | 0.04 | 8.22 | 8.38 | . |
| Other | . | . | . | . | . |
| Marital Status |  |  |  |  |  |
| Single/Never Been Married | 8.26 | 0.06 | 8.13 | 8.38 | 0.01 |
| Married | 8.35 | 0.04 | 8.27 | 8.43 | . |
| Separated | 8.23 | 0.31 | 7.60 | 8.86 | . |
| Divorced | 7.97 | 0.24 | 7.50 | 8.44 | . |
| Widowed | 7.91 | 0.15 | 7.62 | 8.20 | . |
| Domestic Partner | 6.90 | 1.43 | 3.15 | 10.64 | . |
| Employment |  |  |  |  |  |
| Employed for an Employer | 8.26 | 0.08 | 8.10 | 8.43 | 0.05 |
| Self-Employed | 8.35 | 0.05 | 8.25 | 8.45 | . |
| Retired | 7.85 | 0.23 | 7.36 | 8.34 | . |
| Student | 8.16 | 0.13 | 7.89 | 8.42 | . |
| Homemaker | 8.35 | 0.05 | 8.26 | 8.45 | . |
| Unemployed and Looking for a Job | 8.15 | 0.13 | 7.89 | 8.42 | . |
| None of These/Other | 8.06 | 0.14 | 7.77 | 8.34 | . |
| Education |  |  |  |  |  |
| Up to 8 Years | 8.26 | 0.07 | 8.12 | 8.39 | 0.30 |
| 9-15 Years | 8.32 | 0.04 | 8.25 | 8.39 | . |
| 16+ Years | 8.22 | 0.07 | 8.08 | 8.37 | . |
| Service Attendance |  |  |  |  |  |
| >1/Week | 8.34 | 0.06 | 8.23 | 8.45 | 0.03 |
| 1/Week | 8.33 | 0.06 | 8.22 | 8.44 | . |
| 1-3/Month | 8.18 | 0.08 | 8.02 | 8.34 | . |
| A Few Times a Year | 8.03 | 0.08 | 7.86 | 8.19 | . |
| Never | 8.35 | 0.15 | 8.04 | 8.65 | . |
| Immigration Status |  |  |  |  |  |
| Born in This Country | 8.29 | 0.04 | 8.22 | 8.36 | 0.81 |
| Born in Another Country | 8.11 | 0.24 | 7.55 | 8.68 | . |
| Religion |  |  |  |  |  |
| Christianity | 8.00 | 0.14 | 7.74 | 8.27 | 0.00 |
| Islam | 8.31 | 0.04 | 8.24 | 8.39 | . |
| Hinduism | 8.37 | 0.16 | 8.02 | 8.73 | . |
| Buddhism | 5.50 | . | . | . | . |
| Judaism | . | . | . | . | . |
| Sikhism | . | . | . | . | . |
| Baha'i | . | . | . | . | . |
| Jainism | . | . | . | . | . |
| Shinto | . | . | . | . | . |
| Taoism | 7.00 | . | . | . | . |
| Confucianism | . | . | . | . | . |
| Primal, Animist, or Folk Religion | . | . | . | . | . |
| Spiritism | . | . | . | . | . |
| African-Derived | . | . | . | . | . |
| Chinese | . | . | . | . | . |
| Some Other Religion | 8.00 | . | . | . | . |
| No Religion/Atheist/Agnostic | . | . | . | . | . |
| Race/Ethnicity |  |  |  |  |  |
| Banjar/Melayu Banjar | 8.11 | 0.16 | 7.79 | 8.43 | 0.49 |
| Betawi | 8.42 | 0.15 | 8.11 | 8.74 | . |
| Bugis | 8.33 | 0.12 | 8.09 | 8.57 | . |
| Jawa | 8.35 | 0.05 | 8.25 | 8.45 | . |
| Madura | 8.22 | 0.20 | 7.82 | 8.63 | . |
| Minangkabau | 7.96 | 0.20 | 7.57 | 8.36 | . |
| Sunda/Parahyangan | 8.23 | 0.10 | 8.05 | 8.42 | . |
| Bali | 8.23 | 0.14 | 7.91 | 8.54 | . |
| Batak | 8.06 | 0.13 | 7.80 | 8.32 | . |
| Makasar | 8.28 | 0.28 | 7.61 | 8.95 | . |
| Other | 8.33 | 0.09 | 8.15 | 8.50 | . |

**Table S9a: Nationally-Representative Descriptive Statistics of the Observed Sample (Israel)**

| Variable | Proportion | Frequency |
| --- | --- | --- |
| Age |  |  |
| 18-24 | 0.15 | 553 |
| 25-29 | 0.11 | 407 |
| 30-39 | 0.18 | 666 |
| 40-49 | 0.17 | 616 |
| 50-59 | 0.15 | 542 |
| 60-69 | 0.13 | 469 |
| 70-79 | 0.09 | 336 |
| 80 or Older | 0.02 | 79 |
| Missing | . | . |
| Gender |  |  |
| Male | 0.49 | 1791 |
| Female | 0.51 | 1872 |
| Other | 0.00 | 0 |
| Missing | 0.00 | 6 |
| Marital Status |  |  |
| Single/Never Been Married | 0.23 | 834 |
| Married | 0.56 | 2056 |
| Separated | 0.01 | 48 |
| Divorced | 0.07 | 258 |
| Widowed | 0.06 | 212 |
| Domestic Partner | 0.05 | 193 |
| Missing | 0.02 | 69 |
| Employment |  |  |
| Employed for an Employer | 0.49 | 1793 |
| Self-Employed | 0.12 | 424 |
| Retired | 0.16 | 576 |
| Student | 0.11 | 388 |
| Homemaker | 0.06 | 211 |
| Unemployed and Looking for a Job | 0.04 | 148 |
| None of These/Other | 0.03 | 118 |
| Missing | 0.00 | 10 |
| Education |  |  |
| Up to 8 Years | 0.06 | 224 |
| 9-15 Years | 0.41 | 1517 |
| 16+ Years | 0.52 | 1926 |
| Missing | 0.00 | 2 |
| Service Attendance |  |  |
| >1/Week | 0.18 | 649 |
| 1/Week | 0.14 | 495 |
| 1-3/Month | 0.10 | 374 |
| A Few Times a Year | 0.28 | 1014 |
| Never | 0.31 | 1122 |
| Missing | 0.00 | 14 |
| Immigration Status |  |  |
| Born in This Country | 0.76 | 2796 |
| Born in Another Country | 0.24 | 868 |
| Missing | 0.00 | 5 |
| Religion |  |  |
| Christianity | 0.01 | 39 |
| Islam | 0.18 | 656 |
| Hinduism | . | . |
| Buddhism | . | . |
| Judaism | 0.79 | 2897 |
| Sikhism | . | . |
| Baha'i | 0.00 | 2 |
| Jainism | . | . |
| Shinto | . | . |
| Taoism | 0.00 | 1 |
| Confucianism | . | . |
| Primal, Animist, or Folk Religion | 0.00 | 1 |
| Spiritism | . | . |
| African-Derived | . | . |
| Chinese | . | . |
| Some Other Religion | 0.00 | 5 |
| No Religion/Atheist/Agnostic | 0.02 | 64 |
| Missing | 0.00 | 4 |
| Race/Ethnicity |  |  |
| Jewish | 0.80 | 2926 |
| Arab | 0.18 | 674 |
| Other | 0.01 | 39 |
| Missing | 0.01 | 30 |

**Table S9b: Variations Across Demographic Characteristics (Israel)**

| Variable | Mean | SE | LCI | UCI | Global p-value |
| --- | --- | --- | --- | --- | --- |
| Age |  |  |  |  |  |
| 18-24 | 8.79 | 0.11 | 8.57 | 9.00 | 0.00 |
| 25-29 | 8.67 | 0.13 | 8.40 | 8.94 | . |
| 30-39 | 8.61 | 0.10 | 8.41 | 8.80 | . |
| 40-49 | 8.17 | 0.11 | 7.95 | 8.39 | . |
| 50-59 | 7.93 | 0.11 | 7.71 | 8.15 | . |
| 60-69 | 7.53 | 0.13 | 7.26 | 7.80 | . |
| 70-79 | 6.52 | 0.13 | 6.26 | 6.78 | . |
| 80 or Older | 5.85 | 0.25 | 5.33 | 6.37 | . |
| Gender |  |  |  |  |  |
| Male | 8.27 | 0.08 | 8.10 | 8.43 | 0.00 |
| Female | 7.90 | 0.08 | 7.75 | 8.05 | . |
| Other | 7.00 | . | . | . | . |
| Marital Status |  |  |  |  |  |
| Single/Never Been Married | 8.60 | 0.11 | 8.38 | 8.81 | 0.00 |
| Married | 8.10 | 0.08 | 7.94 | 8.25 | . |
| Separated | 8.04 | 0.20 | 7.60 | 8.47 | . |
| Divorced | 7.53 | 0.16 | 7.22 | 7.85 | . |
| Widowed | 6.58 | 0.20 | 6.17 | 6.98 | . |
| Domestic Partner | 8.08 | 0.13 | 7.82 | 8.33 | . |
| Employment |  |  |  |  |  |
| Employed for an Employer | 8.34 | 0.08 | 8.19 | 8.49 | 0.00 |
| Self-Employed | 8.18 | 0.12 | 7.94 | 8.41 | . |
| Retired | 6.81 | 0.14 | 6.54 | 7.08 | . |
| Student | 8.80 | 0.13 | 8.53 | 9.07 | . |
| Homemaker | 7.43 | 0.28 | 6.85 | 8.00 | . |
| Unemployed and Looking for a Job | 8.35 | 0.22 | 7.90 | 8.81 | . |
| None of These/Other | 8.40 | 0.17 | 8.05 | 8.75 | . |
| Education |  |  |  |  |  |
| Up to 8 Years | 6.94 | 0.27 | 6.38 | 7.50 | 0.00 |
| 9-15 Years | 8.21 | 0.08 | 8.04 | 8.37 | . |
| 16+ Years | 8.11 | 0.08 | 7.95 | 8.27 | . |
| Service Attendance |  |  |  |  |  |
| >1/Week | 8.68 | 0.09 | 8.50 | 8.86 | 0.00 |
| 1/Week | 8.16 | 0.12 | 7.93 | 8.39 | . |
| 1-3/Month | 7.87 | 0.11 | 7.65 | 8.09 | . |
| A Few Times a Year | 7.87 | 0.09 | 7.68 | 8.06 | . |
| Never | 7.95 | 0.11 | 7.73 | 8.17 | . |
| Immigration Status |  |  |  |  |  |
| Born in This Country | 8.29 | 0.07 | 8.14 | 8.44 | 0.00 |
| Born in Another Country | 7.40 | 0.12 | 7.17 | 7.64 | . |
| Religion |  |  |  |  |  |
| Christianity | 8.07 | 0.49 | 6.47 | 9.68 | 0.79 |
| Islam | 7.94 | 0.19 | 7.53 | 8.35 | . |
| Hinduism | . | . | . | . | . |
| Buddhism | . | . | . | . | . |
| Judaism | 8.11 | 0.08 | 7.95 | 8.26 | . |
| Sikhism | . | . | . | . | . |
| Baha'i | 10.00 | . | . | . | . |
| Jainism | . | . | . | . | . |
| Shinto | . | . | . | . | . |
| Taoism | 10.00 | . | . | . | . |
| Confucianism | . | . | . | . | . |
| Primal, Animist, or Folk Religion | 8.00 | . | . | . | . |
| Spiritism | . | . | . | . | . |
| African-Derived | . | . | . | . | . |
| Chinese | . | . | . | . | . |
| Some Other Religion | 7.86 | . | . | . | . |
| No Religion/Atheist/Agnostic | 8.21 | 0.23 | 7.69 | 8.73 | . |
| Race/Ethnicity |  |  |  |  |  |
| Jewish | 8.11 | 0.08 | 7.95 | 8.26 | 0.24 |
| Arab | 7.93 | 0.19 | 7.54 | 8.33 | . |
| Other | 8.48 | 0.25 | 7.71 | 9.26 | . |

**Table S10a: Nationally-Representative Descriptive Statistics of the Observed Sample (Japan)**

| Variable | Proportion | Frequency |
| --- | --- | --- |
| Age |  |  |
| 18-24 | 0.08 | 1589 |
| 25-29 | 0.04 | 806 |
| 30-39 | 0.14 | 2851 |
| 40-49 | 0.16 | 3363 |
| 50-59 | 0.18 | 3770 |
| 60-69 | 0.20 | 4118 |
| 70-79 | 0.17 | 3554 |
| 80 or Older | 0.02 | 493 |
| Missing | . | . |
| Gender |  |  |
| Male | 0.48 | 9847 |
| Female | 0.52 | 10602 |
| Other | 0.00 | 28 |
| Missing | 0.00 | 66 |
| Marital Status |  |  |
| Single/Never Been Married | 0.24 | 5004 |
| Married | 0.58 | 11837 |
| Separated | 0.01 | 190 |
| Divorced | 0.10 | 2126 |
| Widowed | 0.06 | 1179 |
| Domestic Partner | 0.01 | 144 |
| Missing | 0.00 | 64 |
| Employment |  |  |
| Employed for an Employer | 0.53 | 10853 |
| Self-Employed | 0.09 | 1748 |
| Retired | 0.12 | 2535 |
| Student | 0.02 | 491 |
| Homemaker | 0.06 | 1276 |
| Unemployed and Looking for a Job | 0.03 | 622 |
| None of These/Other | 0.15 | 2983 |
| Missing | 0.00 | 36 |
| Education |  |  |
| Up to 8 Years | 0.03 | 567 |
| 9-15 Years | 0.72 | 14893 |
| 16+ Years | 0.25 | 5083 |
| Missing | . | . |
| Service Attendance |  |  |
| >1/Week | 0.02 | 316 |
| 1/Week | 0.02 | 348 |
| 1-3/Month | 0.04 | 862 |
| A Few Times a Year | 0.15 | 3112 |
| Never | 0.77 | 15788 |
| Missing | 0.01 | 117 |
| Immigration Status |  |  |
| Born in This Country | 0.95 | 19548 |
| Born in Another Country | 0.01 | 158 |
| Missing | 0.04 | 837 |
| Religion |  |  |
| Christianity | 0.02 | 381 |
| Islam | 0.00 | 10 |
| Hinduism | 0.00 | 5 |
| Buddhism | 0.33 | 6709 |
| Judaism | 0.00 | 10 |
| Sikhism | 0.00 | 6 |
| Baha'i | 0.00 | 2 |
| Jainism | 0.00 | 11 |
| Shinto | 0.02 | 469 |
| Taoism | 0.00 | 7 |
| Confucianism | 0.00 | 17 |
| Primal, Animist, or Folk Religion | 0.00 | 19 |
| Spiritism | . | . |
| African-Derived | . | . |
| Chinese | . | . |
| Some Other Religion | 0.00 | 46 |
| No Religion/Atheist/Agnostic | 0.61 | 12497 |
| Missing | 0.02 | 355 |
| Race/Ethnicity |  |  |
| No Data | . | . |

**Table S10b: Variations Across Demographic Characteristics (Japan)**

| Variable | Mean | SE | LCI | UCI | Global p-value |
| --- | --- | --- | --- | --- | --- |
| Age |  |  |  |  |  |
| 18-24 | 6.25 | 0.07 | 6.12 | 6.38 | 0.00 |
| 25-29 | 6.13 | 0.08 | 5.97 | 6.29 | . |
| 30-39 | 5.99 | 0.05 | 5.89 | 6.08 | . |
| 40-49 | 5.79 | 0.04 | 5.70 | 5.87 | . |
| 50-59 | 5.73 | 0.04 | 5.66 | 5.81 | . |
| 60-69 | 5.96 | 0.04 | 5.89 | 6.03 | . |
| 70-79 | 6.23 | 0.04 | 6.16 | 6.31 | . |
| 80 or Older | 6.14 | 0.11 | 5.92 | 6.35 | . |
| Gender |  |  |  |  |  |
| Male | 5.85 | 0.02 | 5.80 | 5.89 | 0.00 |
| Female | 6.09 | 0.02 | 6.05 | 6.14 | . |
| Other | 5.23 | 0.43 | 4.36 | 6.10 | . |
| Marital Status |  |  |  |  |  |
| Single/Never Been Married | 5.63 | 0.03 | 5.57 | 5.70 | 0.00 |
| Married | 6.17 | 0.02 | 6.13 | 6.21 | . |
| Separated | 5.84 | 0.19 | 5.47 | 6.22 | . |
| Divorced | 5.61 | 0.06 | 5.49 | 5.73 | . |
| Widowed | 6.17 | 0.08 | 6.01 | 6.34 | . |
| Domestic Partner | 5.49 | 0.22 | 5.06 | 5.92 | . |
| Employment |  |  |  |  |  |
| Employed for an Employer | 6.03 | 0.02 | 5.98 | 6.07 | 0.00 |
| Self-Employed | 6.00 | 0.06 | 5.88 | 6.11 | . |
| Retired | 5.89 | 0.05 | 5.80 | 5.98 | . |
| Student | 6.55 | 0.10 | 6.35 | 6.75 | . |
| Homemaker | 6.07 | 0.06 | 5.95 | 6.18 | . |
| Unemployed and Looking for a Job | 4.88 | 0.08 | 4.72 | 5.04 | . |
| None of These/Other | 5.94 | 0.04 | 5.86 | 6.02 | . |
| Education |  |  |  |  |  |
| Up to 8 Years | 5.26 | 0.11 | 5.04 | 5.48 | 0.00 |
| 9-15 Years | 5.88 | 0.02 | 5.84 | 5.92 | . |
| 16+ Years | 6.33 | 0.03 | 6.27 | 6.40 | . |
| Service Attendance |  |  |  |  |  |
| >1/Week | 6.40 | 0.14 | 6.12 | 6.68 | 0.00 |
| 1/Week | 6.83 | 0.12 | 6.59 | 7.06 | . |
| 1-3/Month | 6.16 | 0.08 | 6.01 | 6.31 | . |
| A Few Times a Year | 6.06 | 0.04 | 5.99 | 6.14 | . |
| Never | 5.92 | 0.02 | 5.88 | 5.96 | . |
| Immigration Status |  |  |  |  |  |
| Born in This Country | 5.97 | 0.02 | 5.94 | 6.01 | 0.38 |
| Born in Another Country | 6.13 | 0.18 | 5.77 | 6.50 | . |
| Religion |  |  |  |  |  |
| Christianity | 6.37 | 0.12 | 6.13 | 6.61 | 0.00 |
| Islam | 6.37 | 0.83 | 4.39 | 8.36 | . |
| Hinduism | 6.96 | 0.41 | 5.60 | 8.32 | . |
| Buddhism | 6.08 | 0.03 | 6.02 | 6.13 | . |
| Judaism | 5.88 | 1.40 | -1.31 | 13.08 | . |
| Sikhism | 6.29 | 0.64 | 4.70 | 7.89 | . |
| Baha'i | . | . | . | . | . |
| Jainism | 5.71 | 0.77 | 3.51 | 7.90 | . |
| Shinto | 6.04 | 0.11 | 5.83 | 6.25 | . |
| Taoism | 7.10 | 0.65 | 5.58 | 8.62 | . |
| Confucianism | 5.91 | 0.39 | 5.07 | 6.75 | . |
| Primal, Animist, or Folk Religion | 6.01 | 0.41 | 5.13 | 6.90 | . |
| Spiritism | . | . | . | . | . |
| African-Derived | . | . | . | . | . |
| Chinese | . | . | . | . | . |
| Some Other Religion | 5.77 | 0.46 | 4.85 | 6.70 | . |
| No Religion/Atheist/Agnostic | 5.90 | 0.02 | 5.86 | 5.95 | . |
| Race/Ethnicity |  |  |  |  |  |
| No Data | . | . | . | . | . |

**Table S11a: Nationally-Representative Descriptive Statistics of the Observed Sample (Kenya)**

| Variable | Proportion | Frequency |
| --- | --- | --- |
| Age |  |  |
| 18-24 | 0.25 | 2868 |
| 25-29 | 0.18 | 2035 |
| 30-39 | 0.23 | 2564 |
| 40-49 | 0.15 | 1708 |
| 50-59 | 0.09 | 1072 |
| 60-69 | 0.06 | 710 |
| 70-79 | 0.03 | 360 |
| 80 or Older | 0.01 | 67 |
| Missing | 0.00 | 5 |
| Gender |  |  |
| Male | 0.49 | 5567 |
| Female | 0.51 | 5813 |
| Other | 0.00 | 2 |
| Missing | 0.00 | 7 |
| Marital Status |  |  |
| Single/Never Been Married | 0.31 | 3531 |
| Married | 0.58 | 6626 |
| Separated | 0.04 | 467 |
| Divorced | 0.01 | 111 |
| Widowed | 0.04 | 464 |
| Domestic Partner | 0.01 | 146 |
| Missing | 0.00 | 43 |
| Employment |  |  |
| Employed for an Employer | 0.13 | 1467 |
| Self-Employed | 0.32 | 3630 |
| Retired | 0.03 | 319 |
| Student | 0.10 | 1136 |
| Homemaker | 0.13 | 1537 |
| Unemployed and Looking for a Job | 0.28 | 3153 |
| None of These/Other | 0.01 | 138 |
| Missing | 0.00 | 9 |
| Education |  |  |
| Up to 8 Years | 0.39 | 4485 |
| 9-15 Years | 0.54 | 6115 |
| 16+ Years | 0.07 | 783 |
| Missing | 0.00 | 6 |
| Service Attendance |  |  |
| >1/Week | 0.24 | 2774 |
| 1/Week | 0.53 | 6063 |
| 1-3/Month | 0.11 | 1219 |
| A Few Times a Year | 0.08 | 855 |
| Never | 0.04 | 465 |
| Missing | 0.00 | 13 |
| Immigration Status |  |  |
| Born in This Country | 0.99 | 11270 |
| Born in Another Country | 0.01 | 117 |
| Missing | 0.00 | 2 |
| Religion |  |  |
| Christianity | 0.91 | 10334 |
| Islam | 0.08 | 918 |
| Hinduism | . | . |
| Buddhism | 0.00 | 1 |
| Judaism | 0.00 | 3 |
| Sikhism | . | . |
| Baha'i | 0.00 | 1 |
| Jainism | 0.00 | 1 |
| Shinto | . | . |
| Taoism | . | . |
| Confucianism | 0.00 | 3 |
| Primal, Animist, or Folk Religion | 0.00 | 7 |
| Spiritism | . | . |
| African-Derived | . | . |
| Chinese | . | . |
| Some Other Religion | 0.00 | 5 |
| No Religion/Atheist/Agnostic | 0.01 | 108 |
| Missing | 0.00 | 9 |
| Race/Ethnicity |  |  |
| Luhya | 0.17 | 1943 |
| Luo | 0.10 | 1120 |
| Kalenjin | 0.12 | 1377 |
| Kamba | 0.11 | 1299 |
| Kikuyu | 0.19 | 2118 |
| Kisii | 0.07 | 789 |
| Maasai | 0.02 | 237 |
| Meru | 0.06 | 630 |
| Kenan Somali/Somali | 0.03 | 396 |
| Miji Kenda Tribes | 0.06 | 708 |
| Embu | 0.02 | 197 |
| Other | 0.05 | 548 |
| Missing | 0.00 | 27 |

**Table S11b: Variations Across Demographic Characteristics (Kenya)**

| Variable | Mean | SE | LCI | UCI | Global p-value |
| --- | --- | --- | --- | --- | --- |
| Age |  |  |  |  |  |
| 18-24 | 8.58 | 0.05 | 8.49 | 8.68 | 0.00 |
| 25-29 | 8.43 | 0.06 | 8.32 | 8.54 | . |
| 30-39 | 8.18 | 0.06 | 8.07 | 8.30 | . |
| 40-49 | 7.75 | 0.10 | 7.55 | 7.95 | . |
| 50-59 | 7.33 | 0.14 | 7.06 | 7.61 | . |
| 60-69 | 7.18 | 0.18 | 6.82 | 7.54 | . |
| 70-79 | 6.74 | 0.30 | 6.13 | 7.34 | . |
| 80 or Older | 6.25 | 0.81 | 4.51 | 7.99 | . |
| Gender |  |  |  |  |  |
| Male | 8.16 | 0.05 | 8.06 | 8.26 | 0.00 |
| Female | 7.97 | 0.06 | 7.85 | 8.09 | . |
| Other | 10.00 | . | . | . | . |
| Marital Status |  |  |  |  |  |
| Single/Never Been Married | 8.44 | 0.05 | 8.34 | 8.54 | 0.00 |
| Married | 8.00 | 0.05 | 7.89 | 8.11 | . |
| Separated | 7.91 | 0.18 | 7.57 | 8.26 | . |
| Divorced | 7.22 | 0.32 | 6.57 | 7.86 | . |
| Widowed | 6.46 | 0.25 | 5.97 | 6.96 | . |
| Domestic Partner | 7.94 | 0.24 | 7.46 | 8.41 | . |
| Employment |  |  |  |  |  |
| Employed for an Employer | 8.17 | 0.08 | 8.02 | 8.32 | 0.00 |
| Self-Employed | 7.92 | 0.07 | 7.79 | 8.05 | . |
| Retired | 6.66 | 0.29 | 6.09 | 7.22 | . |
| Student | 8.63 | 0.06 | 8.51 | 8.75 | . |
| Homemaker | 7.70 | 0.12 | 7.46 | 7.93 | . |
| Unemployed and Looking for a Job | 8.32 | 0.06 | 8.19 | 8.44 | . |
| None of These/Other | 7.68 | 0.29 | 7.09 | 8.27 | . |
| Education |  |  |  |  |  |
| Up to 8 Years | 7.87 | 0.07 | 7.73 | 8.02 | 0.00 |
| 9-15 Years | 8.21 | 0.05 | 8.12 | 8.30 | . |
| 16+ Years | 8.03 | 0.10 | 7.84 | 8.22 | . |
| Service Attendance |  |  |  |  |  |
| >1/Week | 7.96 | 0.08 | 7.79 | 8.12 | 0.00 |
| 1/Week | 8.19 | 0.05 | 8.09 | 8.29 | . |
| 1-3/Month | 7.92 | 0.10 | 7.73 | 8.10 | . |
| A Few Times a Year | 7.95 | 0.09 | 7.77 | 8.13 | . |
| Never | 7.66 | 0.19 | 7.29 | 8.03 | . |
| Immigration Status |  |  |  |  |  |
| Born in This Country | 8.06 | 0.04 | 7.97 | 8.15 | 0.57 |
| Born in Another Country | 8.21 | 0.26 | 7.69 | 8.73 | . |
| Religion |  |  |  |  |  |
| Christianity | 8.08 | 0.04 | 7.99 | 8.17 | 0.00 |
| Islam | 7.89 | 0.21 | 7.46 | 8.31 | . |
| Hinduism | . | . | . | . | . |
| Buddhism | 6.00 | . | . | . | . |
| Judaism | 9.83 | . | . | . | . |
| Sikhism | . | . | . | . | . |
| Baha'i | 7.00 | . | . | . | . |
| Jainism | 7.00 | . | . | . | . |
| Shinto | . | . | . | . | . |
| Taoism | . | . | . | . | . |
| Confucianism | 7.00 | . | . | . | . |
| Primal, Animist, or Folk Religion | 6.85 | . | . | . | . |
| Spiritism | . | . | . | . | . |
| African-Derived | . | . | . | . | . |
| Chinese | . | . | . | . | . |
| Some Other Religion | 9.53 | 0.13 | 8.39 | 10.67 | . |
| No Religion/Atheist/Agnostic | 8.01 | 0.27 | 7.47 | 8.56 | . |
| Race/Ethnicity |  |  |  |  |  |
| Luhya | 8.09 | 0.07 | 7.94 | 8.23 | 0.00 |
| Luo | 7.73 | 0.13 | 7.46 | 8.00 | . |
| Kalenjin | 8.32 | 0.09 | 8.14 | 8.50 | . |
| Kamba | 8.33 | 0.11 | 8.11 | 8.54 | . |
| Kikuyu | 8.01 | 0.10 | 7.81 | 8.21 | . |
| Kisii | 7.67 | 0.21 | 7.26 | 8.08 | . |
| Maasai | 8.94 | 0.25 | 8.43 | 9.44 | . |
| Meru | 8.08 | 0.18 | 7.72 | 8.43 | . |
| Kenan Somali/Somali | 7.82 | 0.42 | 6.92 | 8.73 | . |
| Miji Kenda Tribes | 7.69 | 0.18 | 7.33 | 8.06 | . |
| Embu | 7.95 | 0.15 | 7.63 | 8.26 | . |
| Other | 8.48 | 0.14 | 8.20 | 8.75 | . |

**Table S12a: Nationally-Representative Descriptive Statistics of the Observed Sample (Mexico)**

| Variable | Proportion | Frequency |
| --- | --- | --- |
| Age |  |  |
| 18-24 | 0.17 | 986 |
| 25-29 | 0.11 | 623 |
| 30-39 | 0.23 | 1312 |
| 40-49 | 0.18 | 1027 |
| 50-59 | 0.15 | 873 |
| 60-69 | 0.11 | 611 |
| 70-79 | 0.05 | 277 |
| 80 or Older | 0.01 | 68 |
| Missing | . | . |
| Gender |  |  |
| Male | 0.48 | 2755 |
| Female | 0.52 | 2997 |
| Other | 0.00 | 3 |
| Missing | 0.00 | 21 |
| Marital Status |  |  |
| Single/Never Been Married | 0.25 | 1432 |
| Married | 0.36 | 2089 |
| Separated | 0.07 | 403 |
| Divorced | 0.04 | 230 |
| Widowed | 0.06 | 347 |
| Domestic Partner | 0.19 | 1109 |
| Missing | 0.03 | 166 |
| Employment |  |  |
| Employed for an Employer | 0.33 | 1921 |
| Self-Employed | 0.19 | 1091 |
| Retired | 0.07 | 386 |
| Student | 0.04 | 247 |
| Homemaker | 0.22 | 1257 |
| Unemployed and Looking for a Job | 0.10 | 564 |
| None of These/Other | 0.03 | 169 |
| Missing | 0.02 | 141 |
| Education |  |  |
| Up to 8 Years | 0.22 | 1291 |
| 9-15 Years | 0.55 | 3180 |
| 16+ Years | 0.23 | 1304 |
| Missing | 0.00 | 1 |
| Service Attendance |  |  |
| >1/Week | 0.11 | 609 |
| 1/Week | 0.22 | 1260 |
| 1-3/Month | 0.12 | 676 |
| A Few Times a Year | 0.36 | 2054 |
| Never | 0.20 | 1134 |
| Missing | 0.01 | 43 |
| Immigration Status |  |  |
| Born in This Country | 0.96 | 5517 |
| Born in Another Country | 0.02 | 108 |
| Missing | 0.03 | 151 |
| Religion |  |  |
| Christianity | 0.84 | 4844 |
| Islam | 0.00 | 2 |
| Hinduism | 0.00 | 3 |
| Buddhism | 0.00 | 6 |
| Judaism | 0.00 | 7 |
| Sikhism | . | . |
| Baha'i | 0.00 | 1 |
| Jainism | 0.00 | 1 |
| Shinto | 0.00 | 2 |
| Taoism | 0.00 | 4 |
| Confucianism | 0.00 | 0 |
| Primal, Animist, or Folk Religion | 0.00 | 20 |
| Spiritism | . | . |
| African-Derived | . | . |
| Chinese | . | . |
| Some Other Religion | 0.01 | 41 |
| No Religion/Atheist/Agnostic | 0.13 | 770 |
| Missing | 0.01 | 75 |
| Race/Ethnicity |  |  |
| White | 0.19 | 1116 |
| Mestizo | 0.48 | 2762 |
| Indigenous | 0.10 | 594 |
| Black | 0.02 | 108 |
| Mulatto | 0.01 | 63 |
| Other | 0.06 | 339 |
| Missing | 0.14 | 794 |

**Table S12b: Variations Across Demographic Characteristics (Mexico)**

| Variable | Mean | SE | LCI | UCI | Global p-value |
| --- | --- | --- | --- | --- | --- |
| Age |  |  |  |  |  |
| 18-24 | 8.00 | 0.07 | 7.87 | 8.14 | 0.00 |
| 25-29 | 7.81 | 0.09 | 7.64 | 7.98 | . |
| 30-39 | 7.96 | 0.07 | 7.84 | 8.09 | . |
| 40-49 | 7.70 | 0.08 | 7.54 | 7.85 | . |
| 50-59 | 7.75 | 0.09 | 7.58 | 7.92 | . |
| 60-69 | 7.35 | 0.12 | 7.12 | 7.59 | . |
| 70-79 | 7.59 | 0.17 | 7.24 | 7.93 | . |
| 80 or Older | 7.48 | 0.50 | 6.47 | 8.49 | . |
| Gender |  |  |  |  |  |
| Male | 7.99 | 0.05 | 7.90 | 8.09 | 0.00 |
| Female | 7.60 | 0.05 | 7.51 | 7.69 | . |
| Other | 6.25 | 0.54 | 4.83 | 7.67 | . |
| Marital Status |  |  |  |  |  |
| Single/Never Been Married | 7.71 | 0.07 | 7.58 | 7.84 | 0.15 |
| Married | 7.81 | 0.06 | 7.70 | 7.92 | . |
| Separated | 7.62 | 0.12 | 7.39 | 7.85 | . |
| Divorced | 7.69 | 0.14 | 7.42 | 7.97 | . |
| Widowed | 7.73 | 0.14 | 7.45 | 8.01 | . |
| Domestic Partner | 7.94 | 0.07 | 7.80 | 8.07 | . |
| Employment |  |  |  |  |  |
| Employed for an Employer | 7.94 | 0.06 | 7.83 | 8.06 | 0.01 |
| Self-Employed | 7.84 | 0.07 | 7.70 | 7.99 | . |
| Retired | 7.61 | 0.13 | 7.37 | 7.86 | . |
| Student | 7.44 | 0.16 | 7.12 | 7.76 | . |
| Homemaker | 7.69 | 0.07 | 7.54 | 7.83 | . |
| Unemployed and Looking for a Job | 7.66 | 0.10 | 7.46 | 7.86 | . |
| None of These/Other | 7.67 | 0.23 | 7.21 | 8.13 | . |
| Education |  |  |  |  |  |
| Up to 8 Years | 7.64 | 0.08 | 7.48 | 7.80 | 0.10 |
| 9-15 Years | 7.82 | 0.04 | 7.74 | 7.90 | . |
| 16+ Years | 7.85 | 0.07 | 7.72 | 7.97 | . |
| Service Attendance |  |  |  |  |  |
| >1/Week | 8.04 | 0.10 | 7.83 | 8.24 | 0.12 |
| 1/Week | 7.76 | 0.07 | 7.62 | 7.91 | . |
| 1-3/Month | 7.73 | 0.10 | 7.53 | 7.93 | . |
| A Few Times a Year | 7.79 | 0.05 | 7.69 | 7.89 | . |
| Never | 7.71 | 0.07 | 7.56 | 7.85 | . |
| Immigration Status |  |  |  |  |  |
| Born in This Country | 7.79 | 0.03 | 7.72 | 7.86 | 0.38 |
| Born in Another Country | 7.60 | 0.21 | 7.18 | 8.01 | . |
| Religion |  |  |  |  |  |
| Christianity | 7.81 | 0.04 | 7.74 | 7.88 | 0.00 |
| Islam | 9.06 | . | . | . | . |
| Hinduism | 8.95 | 0.37 | 7.38 | 10.52 | . |
| Buddhism | 3.48 | 2.22 | -3.50 | 10.46 | . |
| Judaism | 9.31 | 0.26 | 8.61 | 10.01 | . |
| Sikhism | . | . | . | . | . |
| Baha'i | 8.96 | . | . | . | . |
| Jainism | 5.00 | . | . | . | . |
| Shinto | . | . | . | . | . |
| Taoism | . | . | . | . | . |
| Confucianism | 8.00 | . | . | . | . |
| Primal, Animist, or Folk Religion | 8.74 | 0.42 | 7.80 | 9.68 | . |
| Spiritism | . | . | . | . | . |
| African-Derived | . | . | . | . | . |
| Chinese | . | . | . | . | . |
| Some Other Religion | 7.65 | 0.31 | 7.04 | 8.27 | . |
| No Religion/Atheist/Agnostic | 7.62 | 0.08 | 7.46 | 7.78 | . |
| Race/Ethnicity |  |  |  |  |  |
| White | 7.88 | 0.08 | 7.72 | 8.04 | 0.66 |
| Mestizo | 7.75 | 0.04 | 7.66 | 7.84 | . |
| Indigenous | 7.78 | 0.10 | 7.59 | 7.97 | . |
| Black | 8.02 | 0.25 | 7.52 | 8.52 | . |
| Mulatto | 7.55 | 0.36 | 6.83 | 8.27 | . |
| Other | 7.74 | 0.14 | 7.46 | 8.03 | . |

**Table S13a: Nationally-Representative Descriptive Statistics of the Observed Sample (Nigeria)**

| Variable | Proportion | Frequency |
| --- | --- | --- |
| Age |  |  |
| 18-24 | 0.22 | 1533 |
| 25-29 | 0.17 | 1193 |
| 30-39 | 0.28 | 1943 |
| 40-49 | 0.16 | 1059 |
| 50-59 | 0.09 | 619 |
| 60-69 | 0.04 | 296 |
| 70-79 | 0.02 | 133 |
| 80 or Older | 0.01 | 50 |
| Missing | . | . |
| Gender |  |  |
| Male | 0.49 | 3371 |
| Female | 0.51 | 3456 |
| Other | 0.00 | 0 |
| Missing | . | . |
| Marital Status |  |  |
| Single/Never Been Married | 0.34 | 2289 |
| Married | 0.60 | 4065 |
| Separated | 0.02 | 117 |
| Divorced | 0.01 | 71 |
| Widowed | 0.03 | 231 |
| Domestic Partner | 0.00 | 12 |
| Missing | 0.01 | 42 |
| Employment |  |  |
| Employed for an Employer | 0.10 | 699 |
| Self-Employed | 0.57 | 3898 |
| Retired | 0.03 | 178 |
| Student | 0.10 | 650 |
| Homemaker | 0.07 | 499 |
| Unemployed and Looking for a Job | 0.10 | 684 |
| None of These/Other | 0.03 | 211 |
| Missing | 0.00 | 8 |
| Education |  |  |
| Up to 8 Years | 0.38 | 2575 |
| 9-15 Years | 0.60 | 4120 |
| 16+ Years | 0.02 | 130 |
| Missing | 0.00 | 2 |
| Service Attendance |  |  |
| >1/Week | 0.59 | 4049 |
| 1/Week | 0.28 | 1895 |
| 1-3/Month | 0.08 | 531 |
| A Few Times a Year | 0.04 | 254 |
| Never | 0.01 | 77 |
| Missing | 0.00 | 20 |
| Immigration Status |  |  |
| Born in This Country | 0.99 | 6779 |
| Born in Another Country | 0.01 | 47 |
| Missing | 0.00 | 1 |
| Religion |  |  |
| Christianity | 0.51 | 3476 |
| Islam | 0.48 | 3302 |
| Hinduism | . | . |
| Buddhism | . | . |
| Judaism | . | . |
| Sikhism | . | . |
| Baha'i | . | . |
| Jainism | . | . |
| Shinto | 0.00 | 0 |
| Taoism | . | . |
| Confucianism | 0.00 | 0 |
| Primal, Animist, or Folk Religion | 0.00 | 24 |
| Spiritism | . | . |
| African-Derived | . | . |
| Chinese | . | . |
| Some Other Religion | 0.00 | 1 |
| No Religion/Atheist/Agnostic | 0.00 | 15 |
| Missing | 0.00 | 9 |
| Race/Ethnicity |  |  |
| Hausa | 0.34 | 2342 |
| Yoruba | 0.18 | 1230 |
| Igbo (Ibo) | 0.16 | 1112 |
| Edo | 0.02 | 116 |
| Urhobo | 0.01 | 38 |
| Fulani | 0.04 | 266 |
| Kanuri | 0.00 | 31 |
| Tiv | 0.03 | 198 |
| Efik | 0.01 | 48 |
| Ijaw | 0.02 | 110 |
| Igala | 0.01 | 77 |
| Ibibio | 0.03 | 180 |
| Idoma | 0.01 | 61 |
| Other | 0.15 | 1014 |
| Missing | 0.00 | 4 |

**Table S13b: Variations Across Demographic Characteristics (Nigeria)**

| Variable | Mean | SE | LCI | UCI | Global p-value |
| --- | --- | --- | --- | --- | --- |
| Age |  |  |  |  |  |
| 18-24 | 8.49 | 0.07 | 8.35 | 8.62 | 0.00 |
| 25-29 | 8.49 | 0.06 | 8.37 | 8.61 | . |
| 30-39 | 8.36 | 0.06 | 8.23 | 8.48 | . |
| 40-49 | 8.11 | 0.11 | 7.90 | 8.32 | . |
| 50-59 | 7.89 | 0.20 | 7.50 | 8.29 | . |
| 60-69 | 7.95 | 0.31 | 7.33 | 8.57 | . |
| 70-79 | 6.91 | 0.28 | 6.30 | 7.52 | . |
| 80 or Older | 5.83 | 1.45 | 1.09 | 10.57 | . |
| Gender |  |  |  |  |  |
| Male | 8.28 | 0.06 | 8.17 | 8.40 | 0.00 |
| Female | 8.25 | 0.06 | 8.12 | 8.38 | . |
| Other | 9.00 | . | . | . | . |
| Marital Status |  |  |  |  |  |
| Single/Never Been Married | 8.49 | 0.05 | 8.39 | 8.59 | 0.00 |
| Married | 8.22 | 0.07 | 8.09 | 8.34 | . |
| Separated | 7.25 | 0.28 | 6.70 | 7.80 | . |
| Divorced | 7.96 | 0.30 | 7.34 | 8.57 | . |
| Widowed | 7.54 | 0.51 | 6.51 | 8.57 | . |
| Domestic Partner | 8.26 | 0.20 | 6.51 | 10.01 | . |
| Employment |  |  |  |  |  |
| Employed for an Employer | 8.37 | 0.11 | 8.16 | 8.58 | 0.02 |
| Self-Employed | 8.34 | 0.06 | 8.22 | 8.45 | . |
| Retired | 7.89 | 0.39 | 7.10 | 8.68 | . |
| Student | 8.43 | 0.12 | 8.20 | 8.66 | . |
| Homemaker | 7.76 | 0.19 | 7.39 | 8.13 | . |
| Unemployed and Looking for a Job | 8.16 | 0.14 | 7.89 | 8.44 | . |
| None of These/Other | 7.90 | 0.40 | 7.10 | 8.71 | . |
| Education |  |  |  |  |  |
| Up to 8 Years | 8.10 | 0.11 | 7.87 | 8.32 | 0.07 |
| 9-15 Years | 8.37 | 0.04 | 8.29 | 8.44 | . |
| 16+ Years | 8.33 | 0.14 | 8.06 | 8.60 | . |
| Service Attendance |  |  |  |  |  |
| >1/Week | 8.41 | 0.06 | 8.30 | 8.52 | 0.00 |
| 1/Week | 8.15 | 0.11 | 7.94 | 8.36 | . |
| 1-3/Month | 7.79 | 0.16 | 7.47 | 8.11 | . |
| A Few Times a Year | 8.05 | 0.20 | 7.67 | 8.44 | . |
| Never | 7.53 | 0.52 | 6.48 | 8.57 | . |
| Immigration Status |  |  |  |  |  |
| Born in This Country | 8.27 | 0.05 | 8.17 | 8.37 | 0.67 |
| Born in Another Country | 8.07 | 0.44 | 7.12 | 9.03 | . |
| Religion |  |  |  |  |  |
| Christianity | 8.11 | 0.08 | 7.95 | 8.27 | 0.00 |
| Islam | 8.43 | 0.06 | 8.31 | 8.56 | . |
| Hinduism | . | . | . | . | . |
| Buddhism | . | . | . | . | . |
| Judaism | . | . | . | . | . |
| Sikhism | . | . | . | . | . |
| Baha'i | . | . | . | . | . |
| Jainism | . | . | . | . | . |
| Shinto | 6.00 | . | . | . | . |
| Taoism | . | . | . | . | . |
| Confucianism | 9.00 | . | . | . | . |
| Primal, Animist, or Folk Religion | 8.57 | 0.62 | 6.94 | 10.19 | . |
| Spiritism | . | . | . | . | . |
| African-Derived | . | . | . | . | . |
| Chinese | . | . | . | . | . |
| Some Other Religion | 8.00 | . | . | . | . |
| No Religion/Atheist/Agnostic | 7.87 | 0.27 | 5.54 | 10.19 | . |
| Race/Ethnicity |  |  |  |  |  |
| Hausa | 8.43 | 0.08 | 8.28 | 8.58 | 0.02 |
| Yoruba | 8.23 | 0.17 | 7.88 | 8.58 | . |
| Igbo (Ibo) | 8.17 | 0.11 | 7.95 | 8.39 | . |
| Edo | 7.97 | 0.30 | 7.36 | 8.58 | . |
| Urhobo | 8.49 | 0.18 | 8.10 | 8.88 | . |
| Fulani | 8.49 | 0.22 | 8.04 | 8.94 | . |
| Kanuri | 8.27 | 0.14 | 7.95 | 8.59 | . |
| Tiv | 8.54 | 0.22 | 8.08 | 9.00 | . |
| Efik | 8.88 | 0.35 | 8.15 | 9.62 | . |
| Ijaw | 7.43 | 0.45 | 6.41 | 8.46 | . |
| Igala | 7.90 | 0.24 | 7.41 | 8.39 | . |
| Ibibio | 7.66 | 0.31 | 7.01 | 8.31 | . |
| Idoma | 7.91 | 0.39 | 7.12 | 8.71 | . |
| Other | 8.16 | 0.10 | 7.96 | 8.36 | . |

**Table S14a: Nationally-Representative Descriptive Statistics of the Observed Sample (Philippines)**

| Variable | Proportion | Frequency |
| --- | --- | --- |
| Age |  |  |
| 18-24 | 0.20 | 1073 |
| 25-29 | 0.13 | 695 |
| 30-39 | 0.22 | 1160 |
| 40-49 | 0.18 | 972 |
| 50-59 | 0.14 | 732 |
| 60-69 | 0.09 | 495 |
| 70-79 | 0.03 | 143 |
| 80 or Older | 0.00 | 23 |
| Missing | . | . |
| Gender |  |  |
| Male | 0.50 | 2625 |
| Female | 0.50 | 2643 |
| Other | 0.00 | 13 |
| Missing | 0.00 | 11 |
| Marital Status |  |  |
| Single/Never Been Married | 0.23 | 1206 |
| Married | 0.45 | 2385 |
| Separated | 0.05 | 249 |
| Divorced | 0.00 | 9 |
| Widowed | 0.05 | 274 |
| Domestic Partner | 0.22 | 1152 |
| Missing | 0.00 | 16 |
| Employment |  |  |
| Employed for an Employer | 0.26 | 1350 |
| Self-Employed | 0.26 | 1379 |
| Retired | 0.03 | 158 |
| Student | 0.11 | 585 |
| Homemaker | 0.20 | 1049 |
| Unemployed and Looking for a Job | 0.12 | 658 |
| None of These/Other | 0.02 | 113 |
| Missing | . | . |
| Education |  |  |
| Up to 8 Years | 0.22 | 1188 |
| 9-15 Years | 0.70 | 3722 |
| 16+ Years | 0.07 | 381 |
| Missing | 0.00 | 1 |
| Service Attendance |  |  |
| >1/Week | 0.16 | 844 |
| 1/Week | 0.36 | 1929 |
| 1-3/Month | 0.26 | 1374 |
| A Few Times a Year | 0.18 | 929 |
| Never | 0.04 | 210 |
| Missing | 0.00 | 6 |
| Immigration Status |  |  |
| Born in This Country | 1.00 | 5284 |
| Born in Another Country | 0.00 | 8 |
| Missing | . | . |
| Religion |  |  |
| Christianity | 0.93 | 4914 |
| Islam | 0.06 | 297 |
| Hinduism | . | . |
| Buddhism | 0.00 | 4 |
| Judaism | 0.00 | 4 |
| Sikhism | . | . |
| Baha'i | 0.00 | 1 |
| Jainism | . | . |
| Shinto | . | . |
| Taoism | . | . |
| Confucianism | . | . |
| Primal, Animist, or Folk Religion | 0.00 | 5 |
| Spiritism | . | . |
| African-Derived | . | . |
| Chinese | . | . |
| Some Other Religion | 0.01 | 35 |
| No Religion/Atheist/Agnostic | 0.00 | 23 |
| Missing | 0.00 | 9 |
| Race/Ethnicity |  |  |
| Tagalog | 0.32 | 1691 |
| Cebuana | 0.12 | 656 |
| Ilocano/Ilokano | 0.08 | 429 |
| Visayan/Bisaya | 0.14 | 739 |
| Ilonggo/Hiligaynon | 0.08 | 428 |
| Bicolano/Bikolano | 0.06 | 300 |
| Waray | 0.04 | 216 |
| Tausug | 0.02 | 94 |
| Maranao | 0.01 | 39 |
| Maguindanaoan | 0.02 | 84 |
| Chinese-Filipino | 0.00 | 3 |
| Kapampangan | 0.02 | 107 |
| Pangasinese | 0.02 | 107 |
| Zamboangueno | 0.01 | 51 |
| Malay | . | . |
| Masbateno | 0.01 | 54 |
| Aeta | 0.00 | 1 |
| Igorot | 0.01 | 42 |
| Mangyan | 0.00 | 2 |
| Badjao | 0.00 | 2 |
| Other | 0.05 | 244 |
| Missing | 0.00 | 3 |

**Table S14b: Variations Across Demographic Characteristics (Philippines)**

| Variable | Mean | SE | LCI | UCI | Global p-value |
| --- | --- | --- | --- | --- | --- |
| Age |  |  |  |  |  |
| 18-24 | 7.92 | 0.07 | 7.78 | 8.06 | 0.00 |
| 25-29 | 7.88 | 0.11 | 7.66 | 8.10 | . |
| 30-39 | 7.83 | 0.06 | 7.71 | 7.94 | . |
| 40-49 | 7.54 | 0.07 | 7.40 | 7.69 | . |
| 50-59 | 7.48 | 0.10 | 7.28 | 7.68 | . |
| 60-69 | 7.36 | 0.16 | 7.05 | 7.68 | . |
| 70-79 | 7.34 | 0.21 | 6.91 | 7.76 | . |
| 80 or Older | 6.56 | 0.29 | 5.63 | 7.49 | . |
| Gender |  |  |  |  |  |
| Male | 7.67 | 0.06 | 7.55 | 7.79 | 0.80 |
| Female | 7.72 | 0.04 | 7.64 | 7.80 | . |
| Other | 7.56 | 0.39 | 6.59 | 8.53 | . |
| Marital Status |  |  |  |  |  |
| Single/Never Been Married | 7.69 | 0.08 | 7.53 | 7.84 | 0.03 |
| Married | 7.69 | 0.05 | 7.59 | 7.80 | . |
| Separated | 7.28 | 0.17 | 6.94 | 7.62 | . |
| Divorced | 6.05 | 0.56 | 1.21 | 10.89 | . |
| Widowed | 7.67 | 0.15 | 7.38 | 7.97 | . |
| Domestic Partner | 7.80 | 0.07 | 7.66 | 7.94 | . |
| Employment |  |  |  |  |  |
| Employed for an Employer | 7.81 | 0.07 | 7.66 | 7.95 | 0.04 |
| Self-Employed | 7.69 | 0.07 | 7.55 | 7.83 | . |
| Retired | 7.20 | 0.25 | 6.70 | 7.71 | . |
| Student | 7.82 | 0.09 | 7.65 | 8.00 | . |
| Homemaker | 7.69 | 0.07 | 7.55 | 7.84 | . |
| Unemployed and Looking for a Job | 7.53 | 0.13 | 7.28 | 7.78 | . |
| None of These/Other | 7.19 | 0.19 | 6.80 | 7.57 | . |
| Education |  |  |  |  |  |
| Up to 8 Years | 7.66 | 0.10 | 7.47 | 7.85 | 0.88 |
| 9-15 Years | 7.70 | 0.04 | 7.62 | 7.77 | . |
| 16+ Years | 7.75 | 0.13 | 7.50 | 7.99 | . |
| Service Attendance |  |  |  |  |  |
| >1/Week | 7.77 | 0.11 | 7.55 | 7.99 | 0.00 |
| 1/Week | 7.86 | 0.06 | 7.75 | 7.97 | . |
| 1-3/Month | 7.66 | 0.07 | 7.53 | 7.80 | . |
| A Few Times a Year | 7.48 | 0.08 | 7.32 | 7.65 | . |
| Never | 6.91 | 0.22 | 6.47 | 7.35 | . |
| Immigration Status |  |  |  |  |  |
| Born in This Country | 7.69 | 0.04 | 7.62 | 7.76 | 0.00 |
| Born in Another Country | 6.34 | 0.49 | 4.25 | 8.43 | . |
| Religion |  |  |  |  |  |
| Christianity | 7.68 | 0.04 | 7.61 | 7.75 | 0.00 |
| Islam | 7.83 | 0.20 | 7.43 | 8.23 | . |
| Hinduism | . | . | . | . | . |
| Buddhism | . | . | . | . | . |
| Judaism | 10.00 | . | . | . | . |
| Sikhism | . | . | . | . | . |
| Baha'i | 8.00 | . | . | . | . |
| Jainism | . | . | . | . | . |
| Shinto | . | . | . | . | . |
| Taoism | . | . | . | . | . |
| Confucianism | . | . | . | . | . |
| Primal, Animist, or Folk Religion | 3.10 | 1.39 | -8.91 | 15.12 | . |
| Spiritism | . | . | . | . | . |
| African-Derived | . | . | . | . | . |
| Chinese | . | . | . | . | . |
| Some Other Religion | 7.52 | 0.21 | 7.09 | 7.96 | . |
| No Religion/Atheist/Agnostic | 7.86 | 0.18 | 7.42 | 8.30 | . |
| Race/Ethnicity |  |  |  |  |  |
| Tagalog | 7.76 | 0.06 | 7.64 | 7.88 | 0.00 |
| Cebuana | 7.53 | 0.10 | 7.33 | 7.74 | . |
| Ilocano/Ilokano | 7.87 | 0.16 | 7.55 | 8.18 | . |
| Visayan/Bisaya | 7.63 | 0.09 | 7.46 | 7.81 | . |
| Ilonggo/Hiligaynon | 7.80 | 0.12 | 7.56 | 8.03 | . |
| Bicolano/Bikolano | 7.48 | 0.14 | 7.21 | 7.76 | . |
| Waray | 7.42 | 0.18 | 7.06 | 7.78 | . |
| Tausug | 8.35 | 0.20 | 7.85 | 8.85 | . |
| Maranao | 7.01 | 0.22 | 5.15 | 8.88 | . |
| Maguindanaoan | 8.34 | 0.12 | 8.06 | 8.62 | . |
| Chinese-Filipino | . | . | . | . | . |
| Kapampangan | 7.55 | 0.19 | 7.14 | 7.95 | . |
| Pangasinese | 7.97 | 0.15 | 7.66 | 8.27 | . |
| Zamboangueno | 7.64 | 0.36 | 6.69 | 8.59 | . |
| Malay | . | . | . | . | . |
| Masbateno | 7.77 | 0.16 | 7.31 | 8.23 | . |
| Aeta | 4.33 | . | . | . | . |
| Igorot | 7.83 | 0.17 | 7.34 | 8.33 | . |
| Mangyan | 5.68 | . | . | . | . |
| Badjao | . | . | . | . | . |
| Other | 7.35 | 0.21 | 6.94 | 7.76 | . |

**Table S15a: Nationally-Representative Descriptive Statistics of the Observed Sample (Poland)**

| Variable | Proportion | Frequency |
| --- | --- | --- |
| Age |  |  |
| 18-24 | 0.09 | 955 |
| 25-29 | 0.07 | 761 |
| 30-39 | 0.21 | 2159 |
| 40-49 | 0.19 | 1956 |
| 50-59 | 0.16 | 1670 |
| 60-69 | 0.18 | 1909 |
| 70-79 | 0.08 | 833 |
| 80 or Older | 0.01 | 145 |
| Missing | 0.00 | 1 |
| Gender |  |  |
| Male | 0.48 | 4974 |
| Female | 0.52 | 5387 |
| Other | 0.00 | 3 |
| Missing | 0.00 | 26 |
| Marital Status |  |  |
| Single/Never Been Married | 0.17 | 1811 |
| Married | 0.58 | 6065 |
| Separated | 0.01 | 111 |
| Divorced | 0.05 | 529 |
| Widowed | 0.10 | 990 |
| Domestic Partner | 0.05 | 504 |
| Missing | 0.04 | 379 |
| Employment |  |  |
| Employed for an Employer | 0.56 | 5837 |
| Self-Employed | 0.07 | 686 |
| Retired | 0.23 | 2434 |
| Student | 0.05 | 515 |
| Homemaker | 0.03 | 338 |
| Unemployed and Looking for a Job | 0.03 | 284 |
| None of These/Other | 0.02 | 169 |
| Missing | 0.01 | 126 |
| Education |  |  |
| Up to 8 Years | 0.12 | 1238 |
| 9-15 Years | 0.59 | 6130 |
| 16+ Years | 0.29 | 3020 |
| Missing | 0.00 | 1 |
| Service Attendance |  |  |
| >1/Week | 0.03 | 305 |
| 1/Week | 0.31 | 3263 |
| 1-3/Month | 0.20 | 2081 |
| A Few Times a Year | 0.29 | 3064 |
| Never | 0.15 | 1597 |
| Missing | 0.01 | 78 |
| Immigration Status |  |  |
| Born in This Country | 0.99 | 10258 |
| Born in Another Country | 0.01 | 108 |
| Missing | 0.00 | 23 |
| Religion |  |  |
| Christianity | 0.90 | 9378 |
| Islam | 0.00 | 2 |
| Hinduism | . | . |
| Buddhism | 0.00 | 2 |
| Judaism | . | . |
| Sikhism | 0.00 | 1 |
| Baha'i | . | . |
| Jainism | 0.00 | 3 |
| Shinto | 0.00 | 1 |
| Taoism | . | . |
| Confucianism | . | . |
| Primal, Animist, or Folk Religion | 0.00 | 11 |
| Spiritism | . | . |
| African-Derived | . | . |
| Chinese | . | . |
| Some Other Religion | . | . |
| No Religion/Atheist/Agnostic | 0.09 | 942 |
| Missing | 0.00 | 50 |
| Race/Ethnicity |  |  |
| Polish | 0.99 | 10309 |
| German | 0.00 | 4 |
| Belarussian | 0.00 | 2 |
| Ukranian | 0.00 | 38 |
| Roma | . | . |
| Russian | . | . |
| Ethnic Jewish | . | . |
| Lemko | . | . |
| Silesia | 0.00 | 14 |
| Kashubians | 0.00 | 3 |
| Other | 0.00 | 4 |
| Missing | 0.00 | 14 |

**Table S15b: Variations Across Demographic Characteristics (Poland)**

| Variable | Mean | SE | LCI | UCI | Global p-value |
| --- | --- | --- | --- | --- | --- |
| Age |  |  |  |  |  |
| 18-24 | 8.76 | 0.11 | 8.54 | 8.98 | 0.00 |
| 25-29 | 8.54 | 0.09 | 8.36 | 8.71 | . |
| 30-39 | 8.24 | 0.07 | 8.10 | 8.38 | . |
| 40-49 | 7.81 | 0.08 | 7.65 | 7.97 | . |
| 50-59 | 7.41 | 0.08 | 7.25 | 7.57 | . |
| 60-69 | 6.88 | 0.09 | 6.69 | 7.06 | . |
| 70-79 | 6.48 | 0.17 | 6.14 | 6.81 | . |
| 80 or Older | 6.46 | 0.24 | 5.98 | 6.94 | . |
| Gender |  |  |  |  |  |
| Male | 7.79 | 0.07 | 7.66 | 7.92 | 0.01 |
| Female | 7.58 | 0.06 | 7.46 | 7.70 | . |
| Other | 6.88 | . | . | . | . |
| Marital Status |  |  |  |  |  |
| Single/Never Been Married | 8.34 | 0.10 | 8.15 | 8.53 | 0.00 |
| Married | 7.68 | 0.06 | 7.56 | 7.80 | . |
| Separated | 7.61 | 0.19 | 7.23 | 7.99 | . |
| Divorced | 7.18 | 0.14 | 6.90 | 7.45 | . |
| Widowed | 6.53 | 0.14 | 6.24 | 6.81 | . |
| Domestic Partner | 8.09 | 0.10 | 7.88 | 8.29 | . |
| Employment |  |  |  |  |  |
| Employed for an Employer | 7.96 | 0.06 | 7.85 | 8.08 | 0.00 |
| Self-Employed | 8.17 | 0.08 | 8.00 | 8.33 | . |
| Retired | 6.65 | 0.09 | 6.47 | 6.84 | . |
| Student | 9.06 | 0.13 | 8.80 | 9.31 | . |
| Homemaker | 7.79 | 0.24 | 7.31 | 8.27 | . |
| Unemployed and Looking for a Job | 7.51 | 0.22 | 7.07 | 7.96 | . |
| None of These/Other | 6.51 | 0.37 | 5.78 | 7.25 | . |
| Education |  |  |  |  |  |
| Up to 8 Years | 6.96 | 0.22 | 6.53 | 7.39 | 0.00 |
| 9-15 Years | 7.74 | 0.05 | 7.64 | 7.85 | . |
| 16+ Years | 7.84 | 0.06 | 7.73 | 7.95 | . |
| Service Attendance |  |  |  |  |  |
| >1/Week | 6.56 | 0.36 | 5.85 | 7.27 | 0.00 |
| 1/Week | 7.66 | 0.08 | 7.50 | 7.82 | . |
| 1-3/Month | 7.54 | 0.09 | 7.36 | 7.72 | . |
| A Few Times a Year | 7.91 | 0.08 | 7.76 | 8.06 | . |
| Never | 7.65 | 0.09 | 7.47 | 7.83 | . |
| Immigration Status |  |  |  |  |  |
| Born in This Country | 7.68 | 0.06 | 7.58 | 7.79 | 0.06 |
| Born in Another Country | 7.09 | 0.31 | 6.46 | 7.71 | . |
| Religion |  |  |  |  |  |
| Christianity | 7.66 | 0.06 | 7.55 | 7.78 | 0.00 |
| Islam | 9.66 | . | . | . | . |
| Hinduism | . | . | . | . | . |
| Buddhism | 5.12 | 1.86 | -11.01 | 21.24 | . |
| Judaism | . | . | . | . | . |
| Sikhism | 5.00 | . | . | . | . |
| Baha'i | . | . | . | . | . |
| Jainism | 7.00 | . | . | . | . |
| Shinto | 7.00 | . | . | . | . |
| Taoism | . | . | . | . | . |
| Confucianism | . | . | . | . | . |
| Primal, Animist, or Folk Religion | . | . | . | . | . |
| Spiritism | . | . | . | . | . |
| African-Derived | . | . | . | . | . |
| Chinese | . | . | . | . | . |
| Some Other Religion | . | . | . | . | . |
| No Religion/Atheist/Agnostic | 7.83 | 0.10 | 7.64 | 8.03 | . |
| Race/Ethnicity |  |  |  |  |  |
| Polish | 7.68 | 0.06 | 7.57 | 7.79 | 0.14 |
| German | 6.82 | . | . | . | . |
| Belarussian | 7.60 | . | . | . | . |
| Ukranian | 7.91 | 0.30 | 7.11 | 8.71 | . |
| Roma | . | . | . | . | . |
| Russian | . | . | . | . | . |
| Ethnic Jewish | . | . | . | . | . |
| Lemko | . | . | . | . | . |
| Silesia | 8.17 | 0.27 | 7.00 | 9.34 | . |
| Kashubians | 7.74 | . | . | . | . |
| Other | . | . | . | . | . |

**Table S16a: Nationally-Representative Descriptive Statistics of the Observed Sample (South Africa)**

| Variable | Proportion | Frequency |
| --- | --- | --- |
| Age |  |  |
| 18-24 | 0.17 | 461 |
| 25-29 | 0.14 | 364 |
| 30-39 | 0.25 | 655 |
| 40-49 | 0.20 | 522 |
| 50-59 | 0.12 | 309 |
| 60-69 | 0.07 | 195 |
| 70-79 | 0.05 | 120 |
| 80 or Older | 0.01 | 17 |
| Missing | 0.00 | 9 |
| Gender |  |  |
| Male | 0.49 | 1288 |
| Female | 0.51 | 1356 |
| Other | 0.00 | 2 |
| Missing | 0.00 | 4 |
| Marital Status |  |  |
| Single/Never Been Married | 0.59 | 1561 |
| Married | 0.20 | 539 |
| Separated | 0.03 | 76 |
| Divorced | 0.02 | 51 |
| Widowed | 0.05 | 133 |
| Domestic Partner | 0.10 | 264 |
| Missing | 0.01 | 28 |
| Employment |  |  |
| Employed for an Employer | 0.21 | 569 |
| Self-Employed | 0.16 | 412 |
| Retired | 0.09 | 243 |
| Student | 0.08 | 204 |
| Homemaker | 0.05 | 137 |
| Unemployed and Looking for a Job | 0.38 | 1008 |
| None of These/Other | 0.03 | 74 |
| Missing | 0.00 | 3 |
| Education |  |  |
| Up to 8 Years | 0.25 | 668 |
| 9-15 Years | 0.68 | 1796 |
| 16+ Years | 0.07 | 183 |
| Missing | 0.00 | 4 |
| Service Attendance |  |  |
| >1/Week | 0.16 | 414 |
| 1/Week | 0.34 | 891 |
| 1-3/Month | 0.22 | 574 |
| A Few Times a Year | 0.16 | 431 |
| Never | 0.13 | 334 |
| Missing | 0.00 | 7 |
| Immigration Status |  |  |
| Born in This Country | 0.95 | 2511 |
| Born in Another Country | 0.05 | 139 |
| Missing | 0.00 | 1 |
| Religion |  |  |
| Christianity | 0.82 | 2163 |
| Islam | 0.02 | 62 |
| Hinduism | 0.00 | 1 |
| Buddhism | 0.00 | 12 |
| Judaism | . | . |
| Sikhism | . | . |
| Baha'i | . | . |
| Jainism | 0.00 | 2 |
| Shinto | 0.00 | 2 |
| Taoism | 0.00 | 1 |
| Confucianism | . | . |
| Primal, Animist, or Folk Religion | 0.05 | 127 |
| Spiritism | . | . |
| African-Derived | . | . |
| Chinese | . | . |
| Some Other Religion | 0.00 | 5 |
| No Religion/Atheist/Agnostic | 0.10 | 253 |
| Missing | 0.01 | 23 |
| Race/Ethnicity |  |  |
| Black | 0.90 | 2381 |
| Asian/Indian | 0.00 | 6 |
| Colored | 0.10 | 252 |
| White | 0.00 | 8 |
| Other | 0.00 | 0 |
| Missing | 0.00 | 3 |

**Table S16b: Variations Across Demographic Characteristics (South Africa)**

| Variable | Mean | SE | LCI | UCI | Global p-value |
| --- | --- | --- | --- | --- | --- |
| Age |  |  |  |  |  |
| 18-24 | 8.24 | 0.12 | 8.01 | 8.48 | 0.00 |
| 25-29 | 7.88 | 0.12 | 7.64 | 8.12 | . |
| 30-39 | 8.02 | 0.11 | 7.81 | 8.24 | . |
| 40-49 | 7.42 | 0.16 | 7.11 | 7.72 | . |
| 50-59 | 6.71 | 0.27 | 6.17 | 7.24 | . |
| 60-69 | 5.98 | 0.40 | 5.17 | 6.78 | . |
| 70-79 | 6.22 | 0.48 | 5.25 | 7.20 | . |
| 80 or Older | 6.13 | 1.59 | -7.62 | 19.88 | . |
| Gender |  |  |  |  |  |
| Male | 7.72 | 0.10 | 7.52 | 7.93 | 0.00 |
| Female | 7.33 | 0.11 | 7.11 | 7.56 | . |
| Other | 8.29 | . | . | . | . |
| Marital Status |  |  |  |  |  |
| Single/Never Been Married | 7.78 | 0.09 | 7.62 | 7.95 | 0.00 |
| Married | 6.96 | 0.19 | 6.58 | 7.34 | . |
| Separated | 6.94 | 0.31 | 6.31 | 7.58 | . |
| Divorced | 7.13 | 0.42 | 6.26 | 8.00 | . |
| Widowed | 6.57 | 0.43 | 5.71 | 7.43 | . |
| Domestic Partner | 7.88 | 0.20 | 7.49 | 8.27 | . |
| Employment |  |  |  |  |  |
| Employed for an Employer | 7.83 | 0.12 | 7.59 | 8.07 | 0.00 |
| Self-Employed | 7.67 | 0.19 | 7.29 | 8.05 | . |
| Retired | 6.00 | 0.38 | 5.24 | 6.76 | . |
| Student | 8.15 | 0.18 | 7.79 | 8.51 | . |
| Homemaker | 6.55 | 0.37 | 5.81 | 7.29 | . |
| Unemployed and Looking for a Job | 7.73 | 0.09 | 7.55 | 7.91 | . |
| None of These/Other | 6.65 | 0.69 | 5.24 | 8.06 | . |
| Education |  |  |  |  |  |
| Up to 8 Years | 6.63 | 0.22 | 6.18 | 7.07 | 0.00 |
| 9-15 Years | 7.80 | 0.07 | 7.67 | 7.93 | . |
| 16+ Years | 8.14 | 0.17 | 7.80 | 8.48 | . |
| Service Attendance |  |  |  |  |  |
| >1/Week | 7.49 | 0.20 | 7.08 | 7.89 | 0.55 |
| 1/Week | 7.69 | 0.12 | 7.45 | 7.92 | . |
| 1-3/Month | 7.43 | 0.15 | 7.13 | 7.73 | . |
| A Few Times a Year | 7.42 | 0.16 | 7.09 | 7.74 | . |
| Never | 7.44 | 0.23 | 7.00 | 7.89 | . |
| Immigration Status |  |  |  |  |  |
| Born in This Country | 7.53 | 0.08 | 7.36 | 7.69 | 0.94 |
| Born in Another Country | 7.50 | 0.42 | 6.64 | 8.35 | . |
| Religion |  |  |  |  |  |
| Christianity | 7.50 | 0.09 | 7.33 | 7.68 | 0.00 |
| Islam | 7.56 | 0.36 | 6.81 | 8.31 | . |
| Hinduism | 9.00 | . | . | . | . |
| Buddhism | 7.41 | 0.23 | 6.76 | 8.06 | . |
| Judaism | . | . | . | . | . |
| Sikhism | . | . | . | . | . |
| Baha'i | . | . | . | . | . |
| Jainism | 9.00 | . | . | . | . |
| Shinto | 10.00 | . | . | . | . |
| Taoism | 9.08 | . | . | . | . |
| Confucianism | . | . | . | . | . |
| Primal, Animist, or Folk Religion | 7.41 | 0.42 | 6.57 | 8.25 | . |
| Spiritism | . | . | . | . | . |
| African-Derived | . | . | . | . | . |
| Chinese | . | . | . | . | . |
| Some Other Religion | . | . | . | . | . |
| No Religion/Atheist/Agnostic | 7.70 | 0.21 | 7.29 | 8.12 | . |
| Race/Ethnicity |  |  |  |  |  |
| Black | 7.53 | 0.09 | 7.36 | 7.70 | 0.00 |
| Asian/Indian | 9.00 | . | . | . | . |
| Colored | 7.47 | 0.21 | 7.03 | 7.90 | . |
| White | 6.46 | 0.26 | 4.25 | 8.68 | . |
| Other | 8.00 | . | . | . | . |

**Table S17a: Nationally-Representative Descriptive Statistics of the Observed Sample (Spain)**

| Variable | Proportion | Frequency |
| --- | --- | --- |
| Age |  |  |
| 18-24 | 0.09 | 594 |
| 25-29 | 0.07 | 450 |
| 30-39 | 0.18 | 1111 |
| 40-49 | 0.22 | 1396 |
| 50-59 | 0.20 | 1252 |
| 60-69 | 0.16 | 977 |
| 70-79 | 0.07 | 467 |
| 80 or Older | 0.01 | 43 |
| Missing | . | . |
| Gender |  |  |
| Male | 0.50 | 3142 |
| Female | 0.50 | 3119 |
| Other | 0.00 | 6 |
| Missing | 0.00 | 22 |
| Marital Status |  |  |
| Single/Never Been Married | 0.28 | 1742 |
| Married | 0.47 | 2947 |
| Separated | 0.04 | 237 |
| Divorced | 0.08 | 518 |
| Widowed | 0.03 | 189 |
| Domestic Partner | 0.09 | 589 |
| Missing | 0.01 | 67 |
| Employment |  |  |
| Employed for an Employer | 0.45 | 2862 |
| Self-Employed | 0.09 | 576 |
| Retired | 0.20 | 1278 |
| Student | 0.07 | 448 |
| Homemaker | 0.05 | 345 |
| Unemployed and Looking for a Job | 0.10 | 646 |
| None of These/Other | 0.02 | 123 |
| Missing | 0.00 | 11 |
| Education |  |  |
| Up to 8 Years | 0.13 | 802 |
| 9-15 Years | 0.66 | 4145 |
| 16+ Years | 0.21 | 1341 |
| Missing | 0.00 | 2 |
| Service Attendance |  |  |
| >1/Week | 0.05 | 317 |
| 1/Week | 0.11 | 662 |
| 1-3/Month | 0.07 | 437 |
| A Few Times a Year | 0.31 | 1972 |
| Never | 0.46 | 2875 |
| Missing | 0.00 | 27 |
| Immigration Status |  |  |
| Born in This Country | 0.87 | 5479 |
| Born in Another Country | 0.13 | 788 |
| Missing | 0.00 | 23 |
| Religion |  |  |
| Christianity | 0.65 | 4074 |
| Islam | 0.02 | 135 |
| Hinduism | 0.00 | 7 |
| Buddhism | 0.01 | 36 |
| Judaism | 0.00 | 4 |
| Sikhism | 0.00 | 3 |
| Baha'i | 0.00 | 2 |
| Jainism | 0.00 | 1 |
| Shinto | . | . |
| Taoism | 0.00 | 5 |
| Confucianism | 0.00 | 3 |
| Primal, Animist, or Folk Religion | 0.00 | 7 |
| Spiritism | . | . |
| African-Derived | . | . |
| Chinese | . | . |
| Some Other Religion | 0.00 | 27 |
| No Religion/Atheist/Agnostic | 0.31 | 1932 |
| Missing | 0.01 | 55 |
| Race/Ethnicity |  |  |
| No Data | . | . |

**Table S17b: Variations Across Demographic Characteristics (Spain)**

| Variable | Mean | SE | LCI | UCI | Global p-value |
| --- | --- | --- | --- | --- | --- |
| Age |  |  |  |  |  |
| 18-24 | 7.34 | 0.10 | 7.15 | 7.54 | 0.00 |
| 25-29 | 7.13 | 0.11 | 6.91 | 7.35 | . |
| 30-39 | 7.02 | 0.07 | 6.89 | 7.15 | . |
| 40-49 | 6.99 | 0.06 | 6.88 | 7.10 | . |
| 50-59 | 6.60 | 0.08 | 6.45 | 6.75 | . |
| 60-69 | 6.46 | 0.11 | 6.24 | 6.67 | . |
| 70-79 | 6.43 | 0.20 | 6.03 | 6.83 | . |
| 80 or Older | 6.71 | 0.49 | 5.71 | 7.71 | . |
| Gender |  |  |  |  |  |
| Male | 6.98 | 0.05 | 6.89 | 7.08 | 0.01 |
| Female | 6.68 | 0.05 | 6.58 | 6.78 | . |
| Other | 7.44 | 0.94 | 2.16 | 12.73 | . |
| Marital Status |  |  |  |  |  |
| Single/Never Been Married | 7.00 | 0.06 | 6.88 | 7.12 | 0.01 |
| Married | 6.71 | 0.05 | 6.61 | 6.82 | . |
| Separated | 6.85 | 0.18 | 6.49 | 7.21 | . |
| Divorced | 6.88 | 0.11 | 6.66 | 7.09 | . |
| Widowed | 6.52 | 0.24 | 6.05 | 6.99 | . |
| Domestic Partner | 7.00 | 0.10 | 6.81 | 7.20 | . |
| Employment |  |  |  |  |  |
| Employed for an Employer | 7.03 | 0.04 | 6.95 | 7.11 | 0.00 |
| Self-Employed | 7.02 | 0.10 | 6.82 | 7.22 | . |
| Retired | 6.32 | 0.10 | 6.12 | 6.52 | . |
| Student | 7.32 | 0.12 | 7.09 | 7.56 | . |
| Homemaker | 6.41 | 0.23 | 5.96 | 6.86 | . |
| Unemployed and Looking for a Job | 6.82 | 0.09 | 6.65 | 7.00 | . |
| None of These/Other | 6.22 | 0.25 | 5.73 | 6.70 | . |
| Education |  |  |  |  |  |
| Up to 8 Years | 6.79 | 0.15 | 6.50 | 7.09 | 0.00 |
| 9-15 Years | 6.74 | 0.04 | 6.66 | 6.82 | . |
| 16+ Years | 7.15 | 0.06 | 7.03 | 7.27 | . |
| Service Attendance |  |  |  |  |  |
| >1/Week | 7.46 | 0.13 | 7.20 | 7.72 | 0.00 |
| 1/Week | 7.26 | 0.12 | 7.02 | 7.50 | . |
| 1-3/Month | 7.07 | 0.14 | 6.79 | 7.34 | . |
| A Few Times a Year | 6.72 | 0.06 | 6.60 | 6.85 | . |
| Never | 6.71 | 0.05 | 6.61 | 6.81 | . |
| Immigration Status |  |  |  |  |  |
| Born in This Country | 6.74 | 0.04 | 6.67 | 6.82 | 0.00 |
| Born in Another Country | 7.48 | 0.08 | 7.33 | 7.63 | . |
| Religion |  |  |  |  |  |
| Christianity | 6.91 | 0.04 | 6.82 | 7.00 | 0.00 |
| Islam | 7.27 | 0.20 | 6.87 | 7.67 | . |
| Hinduism | 5.01 | 0.59 | 2.48 | 7.54 | . |
| Buddhism | 6.20 | 0.51 | 5.16 | 7.24 | . |
| Judaism | 9.06 | 0.72 | 5.97 | 12.16 | . |
| Sikhism | . | . | . | . | . |
| Baha'i | 6.40 | . | . | . | . |
| Jainism | 6.00 | . | . | . | . |
| Shinto | . | . | . | . | . |
| Taoism | . | . | . | . | . |
| Confucianism | 3.25 | 2.44 | -17.86 | 24.37 | . |
| Primal, Animist, or Folk Religion | 7.27 | 0.57 | 6.03 | 8.52 | . |
| Spiritism | . | . | . | . | . |
| African-Derived | . | . | . | . | . |
| Chinese | . | . | . | . | . |
| Some Other Religion | 6.71 | 0.44 | 5.82 | 7.60 | . |
| No Religion/Atheist/Agnostic | 6.67 | 0.06 | 6.55 | 6.78 | . |
| Race/Ethnicity |  |  |  |  |  |
| No Data | . | . | . | . | . |

**Table S18a: Nationally-Representative Descriptive Statistics of the Observed Sample (Sweden)**

| Variable | Proportion | Frequency |
| --- | --- | --- |
| Age |  |  |
| 18-24 | 0.10 | 1515 |
| 25-29 | 0.09 | 1399 |
| 30-39 | 0.16 | 2398 |
| 40-49 | 0.15 | 2221 |
| 50-59 | 0.17 | 2493 |
| 60-69 | 0.14 | 2168 |
| 70-79 | 0.15 | 2253 |
| 80 or Older | 0.04 | 621 |
| Missing | . | . |
| Gender |  |  |
| Male | 0.50 | 7536 |
| Female | 0.50 | 7493 |
| Other | 0.00 | 27 |
| Missing | 0.00 | 12 |
| Marital Status |  |  |
| Single/Never Been Married | 0.26 | 3854 |
| Married | 0.43 | 6408 |
| Separated | 0.03 | 426 |
| Divorced | 0.05 | 801 |
| Widowed | 0.03 | 433 |
| Domestic Partner | 0.20 | 3073 |
| Missing | 0.00 | 72 |
| Employment |  |  |
| Employed for an Employer | 0.52 | 7907 |
| Self-Employed | 0.08 | 1243 |
| Retired | 0.25 | 3832 |
| Student | 0.09 | 1332 |
| Homemaker | 0.00 | 75 |
| Unemployed and Looking for a Job | 0.02 | 324 |
| None of These/Other | 0.02 | 337 |
| Missing | 0.00 | 18 |
| Education |  |  |
| Up to 8 Years | 0.02 | 252 |
| 9-15 Years | 0.72 | 10790 |
| 16+ Years | 0.27 | 4026 |
| Missing | . | . |
| Service Attendance |  |  |
| >1/Week | 0.02 | 236 |
| 1/Week | 0.03 | 434 |
| 1-3/Month | 0.03 | 486 |
| A Few Times a Year | 0.26 | 3950 |
| Never | 0.66 | 9918 |
| Missing | 0.00 | 45 |
| Immigration Status |  |  |
| Born in This Country | 0.92 | 13922 |
| Born in Another Country | 0.07 | 1052 |
| Missing | 0.01 | 94 |
| Religion |  |  |
| Christianity | 0.55 | 8346 |
| Islam | 0.03 | 470 |
| Hinduism | 0.00 | 22 |
| Buddhism | 0.01 | 110 |
| Judaism | 0.00 | 54 |
| Sikhism | 0.00 | 4 |
| Baha'i | 0.00 | 6 |
| Jainism | . | . |
| Shinto | 0.00 | 0 |
| Taoism | 0.00 | 4 |
| Confucianism | . | . |
| Primal, Animist, or Folk Religion | 0.01 | 83 |
| Spiritism | . | . |
| African-Derived | . | . |
| Chinese | . | . |
| Some Other Religion | 0.01 | 198 |
| No Religion/Atheist/Agnostic | 0.38 | 5697 |
| Missing | 0.00 | 74 |
| Race/Ethnicity |  |  |
| No Data | . | . |

**Table S18b: Variations Across Demographic Characteristics (Sweden)**

| Variable | Mean | SE | LCI | UCI | Global p-value |
| --- | --- | --- | --- | --- | --- |
| Age |  |  |  |  |  |
| 18-24 | 6.71 | 0.06 | 6.59 | 6.83 | 0.00 |
| 25-29 | 6.41 | 0.06 | 6.29 | 6.53 | . |
| 30-39 | 6.36 | 0.05 | 6.26 | 6.45 | . |
| 40-49 | 6.20 | 0.05 | 6.09 | 6.31 | . |
| 50-59 | 6.19 | 0.05 | 6.08 | 6.29 | . |
| 60-69 | 6.39 | 0.05 | 6.28 | 6.49 | . |
| 70-79 | 6.69 | 0.05 | 6.59 | 6.79 | . |
| 80 or Older | 6.55 | 0.10 | 6.34 | 6.76 | . |
| Gender |  |  |  |  |  |
| Male | 6.54 | 0.03 | 6.49 | 6.60 | 0.00 |
| Female | 6.27 | 0.03 | 6.21 | 6.33 | . |
| Other | 6.38 | 0.45 | 5.46 | 7.31 | . |
| Marital Status |  |  |  |  |  |
| Single/Never Been Married | 6.21 | 0.04 | 6.12 | 6.30 | 0.00 |
| Married | 6.59 | 0.03 | 6.52 | 6.65 | . |
| Separated | 6.28 | 0.12 | 6.04 | 6.52 | . |
| Divorced | 6.09 | 0.09 | 5.92 | 6.26 | . |
| Widowed | 6.40 | 0.12 | 6.17 | 6.64 | . |
| Domestic Partner | 6.39 | 0.04 | 6.31 | 6.47 | . |
| Employment |  |  |  |  |  |
| Employed for an Employer | 6.44 | 0.03 | 6.39 | 6.49 | 0.00 |
| Self-Employed | 6.71 | 0.08 | 6.56 | 6.87 | . |
| Retired | 6.41 | 0.04 | 6.33 | 6.50 | . |
| Student | 6.61 | 0.07 | 6.48 | 6.75 | . |
| Homemaker | 6.14 | 0.31 | 5.53 | 6.76 | . |
| Unemployed and Looking for a Job | 5.15 | 0.14 | 4.88 | 5.42 | . |
| None of These/Other | 4.86 | 0.17 | 4.54 | 5.19 | . |
| Education |  |  |  |  |  |
| Up to 8 Years | 6.17 | 0.20 | 5.77 | 6.56 | 0.00 |
| 9-15 Years | 6.29 | 0.02 | 6.24 | 6.34 | . |
| 16+ Years | 6.74 | 0.04 | 6.67 | 6.81 | . |
| Service Attendance |  |  |  |  |  |
| >1/Week | 6.78 | 0.20 | 6.38 | 7.19 | 0.00 |
| 1/Week | 6.79 | 0.13 | 6.54 | 7.05 | . |
| 1-3/Month | 6.65 | 0.11 | 6.42 | 6.87 | . |
| A Few Times a Year | 6.58 | 0.04 | 6.51 | 6.66 | . |
| Never | 6.30 | 0.02 | 6.25 | 6.35 | . |
| Immigration Status |  |  |  |  |  |
| Born in This Country | 6.40 | 0.02 | 6.36 | 6.44 | 0.14 |
| Born in Another Country | 6.52 | 0.08 | 6.36 | 6.68 | . |
| Religion |  |  |  |  |  |
| Christianity | 6.53 | 0.03 | 6.48 | 6.59 | 0.00 |
| Islam | 6.71 | 0.14 | 6.43 | 6.99 | . |
| Hinduism | 6.18 | 1.05 | 3.73 | 8.64 | . |
| Buddhism | 6.14 | 0.33 | 5.47 | 6.80 | . |
| Judaism | 6.80 | 0.55 | 5.66 | 7.93 | . |
| Sikhism | 7.00 | . | . | . | . |
| Baha'i | 7.48 | 1.26 | -3.40 | 18.36 | . |
| Jainism | . | . | . | . | . |
| Shinto | 6.00 | . | . | . | . |
| Taoism | . | . | . | . | . |
| Confucianism | . | . | . | . | . |
| Primal, Animist, or Folk Religion | 5.98 | 0.36 | 5.25 | 6.72 | . |
| Spiritism | . | . | . | . | . |
| African-Derived | . | . | . | . | . |
| Chinese | . | . | . | . | . |
| Some Other Religion | 5.62 | 0.24 | 5.15 | 6.09 | . |
| No Religion/Atheist/Agnostic | 6.24 | 0.03 | 6.18 | 6.29 | . |
| Race/Ethnicity |  |  |  |  |  |
| No Data | . | . | . | . | . |

**Table S19a: Nationally-Representative Descriptive Statistics of the Observed Sample (Tanzania)**

| Variable | Proportion | Frequency |
| --- | --- | --- |
| Age |  |  |
| 18-24 | 0.25 | 2284 |
| 25-29 | 0.15 | 1349 |
| 30-39 | 0.23 | 2060 |
| 40-49 | 0.17 | 1503 |
| 50-59 | 0.10 | 912 |
| 60-69 | 0.06 | 575 |
| 70-79 | 0.03 | 297 |
| 80 or Older | 0.01 | 93 |
| Missing | 0.00 | 2 |
| Gender |  |  |
| Male | 0.47 | 4299 |
| Female | 0.53 | 4776 |
| Other | . | . |
| Missing | . | . |
| Marital Status |  |  |
| Single/Never Been Married | 0.25 | 2260 |
| Married | 0.61 | 5577 |
| Separated | 0.04 | 404 |
| Divorced | 0.01 | 103 |
| Widowed | 0.05 | 450 |
| Domestic Partner | 0.03 | 275 |
| Missing | 0.00 | 7 |
| Employment |  |  |
| Employed for an Employer | 0.06 | 513 |
| Self-Employed | 0.51 | 4625 |
| Retired | 0.02 | 139 |
| Student | 0.04 | 319 |
| Homemaker | 0.20 | 1796 |
| Unemployed and Looking for a Job | 0.16 | 1491 |
| None of These/Other | 0.02 | 186 |
| Missing | 0.00 | 6 |
| Education |  |  |
| Up to 8 Years | 0.74 | 6699 |
| 9-15 Years | 0.25 | 2252 |
| 16+ Years | 0.01 | 122 |
| Missing | 0.00 | 2 |
| Service Attendance |  |  |
| >1/Week | 0.29 | 2622 |
| 1/Week | 0.47 | 4268 |
| 1-3/Month | 0.12 | 1082 |
| A Few Times a Year | 0.09 | 814 |
| Never | 0.03 | 288 |
| Missing | 0.00 | 1 |
| Immigration Status |  |  |
| Born in This Country | 1.00 | 9048 |
| Born in Another Country | 0.00 | 25 |
| Missing | 0.00 | 1 |
| Religion |  |  |
| Christianity | 0.62 | 5647 |
| Islam | 0.35 | 3189 |
| Hinduism | . | . |
| Buddhism | . | . |
| Judaism | . | . |
| Sikhism | . | . |
| Baha'i | . | . |
| Jainism | . | . |
| Shinto | . | . |
| Taoism | 0.00 | 1 |
| Confucianism | . | . |
| Primal, Animist, or Folk Religion | 0.00 | 12 |
| Spiritism | . | . |
| African-Derived | . | . |
| Chinese | . | . |
| Some Other Religion | . | . |
| No Religion/Atheist/Agnostic | 0.02 | 216 |
| Missing | 0.00 | 10 |
| Race/Ethnicity |  |  |
| African | 1.00 | 9060 |
| Indian | 0.00 | 3 |
| Arab | 0.00 | 11 |
| Other | . | . |
| Missing | 0.00 | 2 |

**Table S19b: Variations Across Demographic Characteristics (Tanzania)**

| Variable | Mean | SE | LCI | UCI | Global p-value |
| --- | --- | --- | --- | --- | --- |
| Age |  |  |  |  |  |
| 18-24 | 8.61 | 0.07 | 8.47 | 8.76 | 0.00 |
| 25-29 | 8.41 | 0.09 | 8.23 | 8.59 | . |
| 30-39 | 8.11 | 0.08 | 7.95 | 8.27 | . |
| 40-49 | 7.59 | 0.09 | 7.40 | 7.78 | . |
| 50-59 | 6.83 | 0.14 | 6.56 | 7.10 | . |
| 60-69 | 5.71 | 0.24 | 5.24 | 6.17 | . |
| 70-79 | 5.03 | 0.34 | 4.36 | 5.70 | . |
| 80 or Older | 4.59 | 0.50 | 3.56 | 5.62 | . |
| Gender |  |  |  |  |  |
| Male | 7.98 | 0.07 | 7.84 | 8.12 | 0.00 |
| Female | 7.59 | 0.07 | 7.45 | 7.74 | . |
| Other | . | . | . | . | . |
| Marital Status |  |  |  |  |  |
| Single/Never Been Married | 8.51 | 0.08 | 8.37 | 8.66 | 0.00 |
| Married | 7.71 | 0.07 | 7.57 | 7.84 | . |
| Separated | 7.42 | 0.19 | 7.04 | 7.80 | . |
| Divorced | 7.47 | 0.39 | 6.68 | 8.25 | . |
| Widowed | 5.25 | 0.26 | 4.73 | 5.77 | . |
| Domestic Partner | 7.89 | 0.22 | 7.45 | 8.33 | . |
| Employment |  |  |  |  |  |
| Employed for an Employer | 8.40 | 0.12 | 8.16 | 8.63 | 0.00 |
| Self-Employed | 7.83 | 0.07 | 7.69 | 7.97 | . |
| Retired | 5.75 | 0.36 | 5.03 | 6.47 | . |
| Student | 8.84 | 0.12 | 8.60 | 9.09 | . |
| Homemaker | 7.21 | 0.12 | 6.97 | 7.46 | . |
| Unemployed and Looking for a Job | 8.21 | 0.10 | 8.01 | 8.40 | . |
| None of These/Other | 6.44 | 0.47 | 5.50 | 7.38 | . |
| Education |  |  |  |  |  |
| Up to 8 Years | 7.50 | 0.07 | 7.36 | 7.64 | 0.00 |
| 9-15 Years | 8.55 | 0.05 | 8.44 | 8.65 | . |
| 16+ Years | 8.83 | 0.15 | 8.53 | 9.14 | . |
| Service Attendance |  |  |  |  |  |
| >1/Week | 7.67 | 0.10 | 7.48 | 7.86 | 0.08 |
| 1/Week | 7.90 | 0.07 | 7.76 | 8.04 | . |
| 1-3/Month | 7.65 | 0.11 | 7.43 | 7.88 | . |
| A Few Times a Year | 7.73 | 0.14 | 7.45 | 8.01 | . |
| Never | 7.47 | 0.26 | 6.96 | 7.97 | . |
| Immigration Status |  |  |  |  |  |
| Born in This Country | 7.77 | 0.06 | 7.66 | 7.89 | 0.62 |
| Born in Another Country | 8.15 | 0.73 | 6.49 | 9.80 | . |
| Religion |  |  |  |  |  |
| Christianity | 7.89 | 0.07 | 7.76 | 8.03 | 0.00 |
| Islam | 7.58 | 0.09 | 7.40 | 7.76 | . |
| Hinduism | . | . | . | . | . |
| Buddhism | . | . | . | . | . |
| Judaism | . | . | . | . | . |
| Sikhism | . | . | . | . | . |
| Baha'i | . | . | . | . | . |
| Jainism | . | . | . | . | . |
| Shinto | . | . | . | . | . |
| Taoism | 10.00 | . | . | . | . |
| Confucianism | . | . | . | . | . |
| Primal, Animist, or Folk Religion | 8.58 | 0.20 | 7.73 | 9.44 | . |
| Spiritism | . | . | . | . | . |
| African-Derived | . | . | . | . | . |
| Chinese | . | . | . | . | . |
| Some Other Religion | . | . | . | . | . |
| No Religion/Atheist/Agnostic | 7.57 | 0.23 | 7.10 | 8.04 | . |
| Race/Ethnicity |  |  |  |  |  |
| African | 7.78 | 0.06 | 7.66 | 7.89 | 0.04 |
| Indian | 3.94 | . | . | . | . |
| Arab | 7.71 | 0.47 | 5.68 | 9.74 | . |
| Other | . | . | . | . | . |

**Table S20a: Nationally-Representative Descriptive Statistics of the Observed Sample (Türkiye)**

| Variable | Proportion | Frequency |
| --- | --- | --- |
| Age |  |  |
| 18-24 | 0.15 | 222 |
| 25-29 | 0.10 | 152 |
| 30-39 | 0.21 | 315 |
| 40-49 | 0.21 | 312 |
| 50-59 | 0.15 | 225 |
| 60-69 | 0.11 | 164 |
| 70-79 | 0.04 | 65 |
| 80 or Older | 0.01 | 18 |
| Missing | . | . |
| Gender |  |  |
| Male | 0.51 | 754 |
| Female | 0.49 | 719 |
| Other | . | . |
| Missing | . | . |
| Marital Status |  |  |
| Single/Never Been Married | 0.26 | 379 |
| Married | 0.64 | 936 |
| Separated | 0.01 | 13 |
| Divorced | 0.04 | 64 |
| Widowed | 0.04 | 64 |
| Domestic Partner | . | . |
| Missing | 0.01 | 17 |
| Employment |  |  |
| Employed for an Employer | 0.28 | 413 |
| Self-Employed | 0.17 | 255 |
| Retired | 0.14 | 205 |
| Student | 0.07 | 107 |
| Homemaker | 0.24 | 347 |
| Unemployed and Looking for a Job | 0.06 | 87 |
| None of These/Other | 0.04 | 59 |
| Missing | . | . |
| Education |  |  |
| Up to 8 Years | 0.30 | 436 |
| 9-15 Years | 0.48 | 711 |
| 16+ Years | 0.22 | 326 |
| Missing | . | . |
| Service Attendance |  |  |
| >1/Week | 0.33 | 493 |
| 1/Week | 0.18 | 271 |
| 1-3/Month | 0.12 | 174 |
| A Few Times a Year | 0.17 | 255 |
| Never | 0.19 | 274 |
| Missing | 0.00 | 6 |
| Immigration Status |  |  |
| Born in This Country | 0.96 | 1415 |
| Born in Another Country | 0.04 | 58 |
| Missing | . | . |
| Religion |  |  |
| Christianity | 0.00 | 2 |
| Islam | 0.94 | 1381 |
| Hinduism | . | . |
| Buddhism | 0.00 | 0 |
| Judaism | 0.00 | 1 |
| Sikhism | 0.00 | 1 |
| Baha'i | . | . |
| Jainism | . | . |
| Shinto | . | . |
| Taoism | . | . |
| Confucianism | . | . |
| Primal, Animist, or Folk Religion | 0.00 | 1 |
| Spiritism | . | . |
| African-Derived | . | . |
| Chinese | . | . |
| Some Other Religion | 0.00 | 1 |
| No Religion/Atheist/Agnostic | 0.04 | 66 |
| Missing | 0.01 | 19 |
| Race/Ethnicity |  |  |
| Turkish | 0.70 | 1030 |
| Kurdish/Zaza | 0.17 | 252 |
| Arab | 0.03 | 51 |
| Laz | 0.02 | 25 |
| Circassian | 0.01 | 19 |
| Bosnian | 0.00 | 5 |
| Armenian | 0.00 | 1 |
| Georgian | 0.00 | 4 |
| Uyghur | 0.00 | 1 |
| Jewish | . | . |
| Albanian | 0.01 | 8 |
| Greek | 0.00 | 1 |
| Azeri | 0.01 | 9 |
| Other | 0.04 | 58 |
| Missing | 0.01 | 9 |

**Table S20b: Variations Across Demographic Characteristics (Türkiye)**

| Variable | Mean | SE | LCI | UCI | Global p-value |
| --- | --- | --- | --- | --- | --- |
| Age |  |  |  |  |  |
| 18-24 | 7.19 | 0.16 | 6.88 | 7.50 | 0.00 |
| 25-29 | 7.30 | 0.27 | 6.76 | 7.83 | . |
| 30-39 | 6.60 | 0.20 | 6.20 | 6.99 | . |
| 40-49 | 6.39 | 0.18 | 6.04 | 6.73 | . |
| 50-59 | 6.94 | 0.23 | 6.50 | 7.39 | . |
| 60-69 | 6.29 | 0.37 | 5.54 | 7.04 | . |
| 70-79 | 5.93 | 0.42 | 5.01 | 6.84 | . |
| 80 or Older | . | . | . | . | . |
| Gender |  |  |  |  |  |
| Male | 7.00 | 0.11 | 6.77 | 7.22 | 0.00 |
| Female | 6.38 | 0.14 | 6.10 | 6.66 | . |
| Other | . | . | . | . | . |
| Marital Status |  |  |  |  |  |
| Single/Never Been Married | 7.03 | 0.15 | 6.74 | 7.33 | 0.06 |
| Married | 6.63 | 0.11 | 6.41 | 6.85 | . |
| Separated | 7.30 | 0.45 | 5.98 | 8.63 | . |
| Divorced | 6.05 | 0.53 | 4.98 | 7.12 | . |
| Widowed | 6.18 | 0.58 | 4.97 | 7.38 | . |
| Domestic Partner | . | . | . | . | . |
| Employment |  |  |  |  |  |
| Employed for an Employer | 7.19 | 0.13 | 6.94 | 7.44 | 0.00 |
| Self-Employed | 7.05 | 0.22 | 6.62 | 7.49 | . |
| Retired | 6.43 | 0.30 | 5.83 | 7.03 | . |
| Student | 7.00 | 0.21 | 6.58 | 7.41 | . |
| Homemaker | 6.16 | 0.23 | 5.71 | 6.62 | . |
| Unemployed and Looking for a Job | 6.26 | 0.31 | 5.64 | 6.88 | . |
| None of These/Other | 5.86 | 0.48 | 4.87 | 6.84 | . |
| Education |  |  |  |  |  |
| Up to 8 Years | 6.16 | 0.20 | 5.76 | 6.56 | 0.00 |
| 9-15 Years | 6.85 | 0.13 | 6.60 | 7.10 | . |
| 16+ Years | 7.07 | 0.11 | 6.87 | 7.28 | . |
| Service Attendance |  |  |  |  |  |
| >1/Week | 6.75 | 0.17 | 6.42 | 7.08 | 0.08 |
| 1/Week | 7.11 | 0.21 | 6.69 | 7.53 | . |
| 1-3/Month | 6.78 | 0.23 | 6.32 | 7.25 | . |
| A Few Times a Year | 6.48 | 0.20 | 6.08 | 6.88 | . |
| Never | 6.34 | 0.21 | 5.93 | 6.75 | . |
| Immigration Status |  |  |  |  |  |
| Born in This Country | 6.66 | 0.09 | 6.48 | 6.84 | 0.02 |
| Born in Another Country | 7.58 | 0.35 | 6.86 | 8.29 | . |
| Religion |  |  |  |  |  |
| Christianity | . | . | . | . | 0.00 |
| Islam | 6.68 | 0.10 | 6.49 | 6.87 | . |
| Hinduism | . | . | . | . | . |
| Buddhism | 9.00 | . | . | . | . |
| Judaism | 10.00 | . | . | . | . |
| Sikhism | 6.00 | . | . | . | . |
| Baha'i | . | . | . | . | . |
| Jainism | . | . | . | . | . |
| Shinto | . | . | . | . | . |
| Taoism | . | . | . | . | . |
| Confucianism | . | . | . | . | . |
| Primal, Animist, or Folk Religion | 5.57 | . | . | . | . |
| Spiritism | . | . | . | . | . |
| African-Derived | . | . | . | . | . |
| Chinese | . | . | . | . | . |
| Some Other Religion | 6.05 | . | . | . | . |
| No Religion/Atheist/Agnostic | 7.06 | 0.29 | 6.48 | 7.63 | . |
| Race/Ethnicity |  |  |  |  |  |
| Turkish | 6.80 | 0.10 | 6.60 | 7.01 | 0.00 |
| Kurdish/Zaza | 6.17 | 0.27 | 5.64 | 6.70 | . |
| Arab | 6.98 | 0.44 | 6.08 | 7.87 | . |
| Laz | 7.35 | 0.48 | 6.31 | 8.40 | . |
| Circassian | 6.23 | 0.90 | 4.14 | 8.32 | . |
| Bosnian | 7.80 | 1.17 | -2.36 | 17.96 | . |
| Armenian | 8.86 | . | . | . | . |
| Georgian | . | . | . | . | . |
| Uyghur | 8.00 | . | . | . | . |
| Jewish | . | . | . | . | . |
| Albanian | . | . | . | . | . |
| Greek | 8.29 | . | . | . | . |
| Azeri | 8.09 | 0.60 | 6.11 | 10.07 | . |
| Other | 6.54 | 0.51 | 5.49 | 7.58 | . |

**Table S21a: Nationally-Representative Descriptive Statistics of the Observed Sample (United Kingdom)**

| Variable | Proportion | Frequency |
| --- | --- | --- |
| Age |  |  |
| 18-24 | 0.09 | 490 |
| 25-29 | 0.07 | 391 |
| 30-39 | 0.18 | 946 |
| 40-49 | 0.15 | 827 |
| 50-59 | 0.18 | 949 |
| 60-69 | 0.17 | 889 |
| 70-79 | 0.13 | 711 |
| 80 or Older | 0.03 | 163 |
| Missing | 0.00 | 1 |
| Gender |  |  |
| Male | 0.48 | 2557 |
| Female | 0.52 | 2789 |
| Other | 0.00 | 14 |
| Missing | 0.00 | 9 |
| Marital Status |  |  |
| Single/Never Been Married | 0.27 | 1456 |
| Married | 0.47 | 2510 |
| Separated | 0.02 | 114 |
| Divorced | 0.08 | 435 |
| Widowed | 0.05 | 294 |
| Domestic Partner | 0.10 | 512 |
| Missing | 0.01 | 48 |
| Employment |  |  |
| Employed for an Employer | 0.52 | 2798 |
| Self-Employed | 0.09 | 469 |
| Retired | 0.24 | 1262 |
| Student | 0.04 | 229 |
| Homemaker | 0.03 | 184 |
| Unemployed and Looking for a Job | 0.04 | 215 |
| None of These/Other | 0.04 | 201 |
| Missing | 0.00 | 11 |
| Education |  |  |
| Up to 8 Years | 0.24 | 1314 |
| 9-15 Years | 0.39 | 2072 |
| 16+ Years | 0.37 | 1974 |
| Missing | 0.00 | 8 |
| Service Attendance |  |  |
| >1/Week | 0.05 | 291 |
| 1/Week | 0.09 | 499 |
| 1-3/Month | 0.05 | 293 |
| A Few Times a Year | 0.22 | 1165 |
| Never | 0.58 | 3110 |
| Missing | 0.00 | 10 |
| Immigration Status |  |  |
| Born in This Country | 0.87 | 4659 |
| Born in Another Country | 0.13 | 682 |
| Missing | 0.00 | 27 |
| Religion |  |  |
| Christianity | 0.51 | 2750 |
| Islam | 0.04 | 218 |
| Hinduism | 0.01 | 61 |
| Buddhism | 0.01 | 30 |
| Judaism | 0.01 | 44 |
| Sikhism | 0.01 | 29 |
| Baha'i | 0.00 | 6 |
| Jainism | 0.00 | 4 |
| Shinto | . | . |
| Taoism | 0.00 | 4 |
| Confucianism | 0.00 | 2 |
| Primal, Animist, or Folk Religion | 0.01 | 36 |
| Spiritism | . | . |
| African-Derived | . | . |
| Chinese | . | . |
| Some Other Religion | 0.01 | 61 |
| No Religion/Atheist/Agnostic | 0.39 | 2099 |
| Missing | 0.00 | 25 |
| Race/Ethnicity |  |  |
| Asian | 0.08 | 426 |
| Black | 0.03 | 152 |
| White | 0.87 | 4647 |
| Other | 0.02 | 96 |
| Missing | 0.01 | 47 |

**Table S21b: Variations Across Demographic Characteristics (United Kingdom)**

| Variable | Mean | SE | LCI | UCI | Global p-value |
| --- | --- | --- | --- | --- | --- |
| Age |  |  |  |  |  |
| 18-24 | 6.49 | 0.17 | 6.15 | 6.83 | 0.00 |
| 25-29 | 7.03 | 0.14 | 6.75 | 7.31 | . |
| 30-39 | 6.65 | 0.10 | 6.45 | 6.85 | . |
| 40-49 | 6.47 | 0.11 | 6.25 | 6.68 | . |
| 50-59 | 6.41 | 0.09 | 6.22 | 6.59 | . |
| 60-69 | 5.92 | 0.12 | 5.69 | 6.15 | . |
| 70-79 | 6.04 | 0.13 | 5.79 | 6.29 | . |
| 80 or Older | 6.05 | 0.17 | 5.72 | 6.39 | . |
| Gender |  |  |  |  |  |
| Male | 6.48 | 0.06 | 6.35 | 6.60 | 0.00 |
| Female | 6.28 | 0.06 | 6.15 | 6.40 | . |
| Other | 5.57 | 0.29 | 4.87 | 6.26 | . |
| Marital Status |  |  |  |  |  |
| Single/Never Been Married | 6.26 | 0.09 | 6.08 | 6.44 | 0.00 |
| Married | 6.60 | 0.06 | 6.48 | 6.72 | . |
| Separated | 5.83 | 0.28 | 5.28 | 6.38 | . |
| Divorced | 5.63 | 0.17 | 5.29 | 5.96 | . |
| Widowed | 6.11 | 0.19 | 5.74 | 6.48 | . |
| Domestic Partner | 6.47 | 0.13 | 6.22 | 6.72 | . |
| Employment |  |  |  |  |  |
| Employed for an Employer | 6.75 | 0.05 | 6.65 | 6.85 | 0.00 |
| Self-Employed | 6.77 | 0.13 | 6.51 | 7.02 | . |
| Retired | 5.93 | 0.09 | 5.75 | 6.12 | . |
| Student | 6.64 | 0.21 | 6.23 | 7.05 | . |
| Homemaker | 6.06 | 0.27 | 5.54 | 6.59 | . |
| Unemployed and Looking for a Job | 5.56 | 0.35 | 4.87 | 6.24 | . |
| None of These/Other | 3.74 | 0.25 | 3.24 | 4.23 | . |
| Education |  |  |  |  |  |
| Up to 8 Years | 5.80 | 0.13 | 5.54 | 6.06 | 0.00 |
| 9-15 Years | 6.21 | 0.06 | 6.10 | 6.33 | . |
| 16+ Years | 6.92 | 0.05 | 6.82 | 7.02 | . |
| Service Attendance |  |  |  |  |  |
| >1/Week | 7.05 | 0.17 | 6.71 | 7.38 | 0.00 |
| 1/Week | 6.97 | 0.13 | 6.71 | 7.24 | . |
| 1-3/Month | 6.93 | 0.20 | 6.54 | 7.31 | . |
| A Few Times a Year | 6.53 | 0.09 | 6.36 | 6.70 | . |
| Never | 6.10 | 0.06 | 5.98 | 6.22 | . |
| Immigration Status |  |  |  |  |  |
| Born in This Country | 6.30 | 0.05 | 6.21 | 6.39 | 0.00 |
| Born in Another Country | 6.86 | 0.12 | 6.63 | 7.09 | . |
| Religion |  |  |  |  |  |
| Christianity | 6.48 | 0.07 | 6.35 | 6.61 | 0.00 |
| Islam | 6.80 | 0.22 | 6.37 | 7.23 | . |
| Hinduism | 6.50 | 0.30 | 5.88 | 7.11 | . |
| Buddhism | 6.22 | 0.51 | 5.17 | 7.27 | . |
| Judaism | 6.59 | 0.50 | 5.58 | 7.61 | . |
| Sikhism | 7.65 | 0.54 | 6.46 | 8.84 | . |
| Baha'i | . | . | . | . | . |
| Jainism | 5.00 | . | . | . | . |
| Shinto | . | . | . | . | . |
| Taoism | . | . | . | . | . |
| Confucianism | 3.00 | . | . | . | . |
| Primal, Animist, or Folk Religion | 5.95 | 0.43 | 5.03 | 6.87 | . |
| Spiritism | . | . | . | . | . |
| African-Derived | . | . | . | . | . |
| Chinese | . | . | . | . | . |
| Some Other Religion | 6.11 | 0.30 | 5.51 | 6.71 | . |
| No Religion/Atheist/Agnostic | 6.19 | 0.07 | 6.06 | 6.32 | . |
| Race/Ethnicity |  |  |  |  |  |
| Asian | 6.58 | 0.17 | 6.25 | 6.91 | 0.02 |
| Black | 6.93 | 0.22 | 6.50 | 7.36 | . |
| White | 6.33 | 0.05 | 6.23 | 6.42 | . |
| Other | 6.68 | 0.24 | 6.20 | 7.16 | . |

**Table S22a: Nationally-Representative Descriptive Statistics of the Observed Sample (United States)**

| Variable | Proportion | Frequency |
| --- | --- | --- |
| Age |  |  |
| 18-24 | 0.07 | 2682 |
| 25-29 | 0.09 | 3540 |
| 30-39 | 0.19 | 7284 |
| 40-49 | 0.15 | 5649 |
| 50-59 | 0.18 | 6745 |
| 60-69 | 0.18 | 6832 |
| 70-79 | 0.11 | 4054 |
| 80 or Older | 0.04 | 1525 |
| Missing | . | . |
| Gender |  |  |
| Male | 0.48 | 18222 |
| Female | 0.51 | 19562 |
| Other | 0.01 | 392 |
| Missing | 0.00 | 136 |
| Marital Status |  |  |
| Single/Never Been Married | 0.25 | 9431 |
| Married | 0.53 | 20360 |
| Separated | 0.02 | 727 |
| Divorced | 0.09 | 3636 |
| Widowed | 0.05 | 1978 |
| Domestic Partner | 0.05 | 1971 |
| Missing | 0.01 | 207 |
| Employment |  |  |
| Employed for an Employer | 0.51 | 19502 |
| Self-Employed | 0.09 | 3445 |
| Retired | 0.24 | 9016 |
| Student | 0.03 | 1144 |
| Homemaker | 0.05 | 2049 |
| Unemployed and Looking for a Job | 0.05 | 1777 |
| None of These/Other | 0.03 | 1292 |
| Missing | 0.00 | 87 |
| Education |  |  |
| Up to 8 Years | 0.01 | 210 |
| 9-15 Years | 0.66 | 25322 |
| 16+ Years | 0.33 | 12705 |
| Missing | 0.00 | 75 |
| Service Attendance |  |  |
| >1/Week | 0.07 | 2633 |
| 1/Week | 0.15 | 5887 |
| 1-3/Month | 0.07 | 2819 |
| A Few Times a Year | 0.23 | 8870 |
| Never | 0.47 | 17975 |
| Missing | 0.00 | 128 |
| Immigration Status |  |  |
| Born in This Country | 0.91 | 34865 |
| Born in Another Country | 0.08 | 3020 |
| Missing | 0.01 | 427 |
| Religion |  |  |
| Christianity | 0.60 | 22954 |
| Islam | 0.01 | 205 |
| Hinduism | 0.00 | 167 |
| Buddhism | 0.01 | 336 |
| Judaism | 0.02 | 638 |
| Sikhism | 0.00 | 24 |
| Baha'i | 0.00 | 13 |
| Jainism | 0.00 | 18 |
| Shinto | 0.00 | 12 |
| Taoism | 0.00 | 93 |
| Confucianism | 0.00 | 8 |
| Primal, Animist, or Folk Religion | 0.01 | 240 |
| Spiritism | . | . |
| African-Derived | . | . |
| Chinese | . | . |
| Some Other Religion | 0.03 | 1267 |
| No Religion/Atheist/Agnostic | 0.31 | 11870 |
| Missing | 0.01 | 467 |
| Race/Ethnicity |  |  |
| White | 0.62 | 23605 |
| Other | 0.03 | 997 |
| Black | 0.12 | 4501 |
| Asian | 0.06 | 2466 |
| Hispanic | 0.18 | 6724 |
| Other | . | . |
| Missing | 0.00 | 20 |

**Table S22b: Variations Across Demographic Characteristics (United States)**

| Variable | Mean | SE | LCI | UCI | Global p-value |
| --- | --- | --- | --- | --- | --- |
| Age |  |  |  |  |  |
| 18-24 | 6.64 | 0.17 | 6.30 | 6.98 | 0.00 |
| 25-29 | 6.49 | 0.13 | 6.23 | 6.75 | . |
| 30-39 | 6.73 | 0.06 | 6.60 | 6.85 | . |
| 40-49 | 6.61 | 0.05 | 6.51 | 6.72 | . |
| 50-59 | 6.81 | 0.04 | 6.73 | 6.89 | . |
| 60-69 | 7.06 | 0.03 | 7.00 | 7.11 | . |
| 70-79 | 7.11 | 0.04 | 7.04 | 7.18 | . |
| 80 or Older | 7.02 | 0.10 | 6.82 | 7.23 | . |
| Gender |  |  |  |  |  |
| Male | 6.83 | 0.04 | 6.76 | 6.90 | 0.57 |
| Female | 6.79 | 0.04 | 6.72 | 6.86 | . |
| Other | 6.55 | 0.31 | 5.95 | 7.15 | . |
| Marital Status |  |  |  |  |  |
| Single/Never Been Married | 6.40 | 0.07 | 6.26 | 6.54 | 0.00 |
| Married | 7.04 | 0.02 | 6.99 | 7.08 | . |
| Separated | 6.65 | 0.33 | 6.01 | 7.29 | . |
| Divorced | 6.64 | 0.06 | 6.53 | 6.75 | . |
| Widowed | 6.85 | 0.08 | 6.69 | 7.02 | . |
| Domestic Partner | 6.70 | 0.12 | 6.47 | 6.92 | . |
| Employment |  |  |  |  |  |
| Employed for an Employer | 6.88 | 0.03 | 6.82 | 6.94 | 0.00 |
| Self-Employed | 7.08 | 0.10 | 6.89 | 7.27 | . |
| Retired | 6.95 | 0.03 | 6.89 | 7.01 | . |
| Student | 6.76 | 0.25 | 6.27 | 7.25 | . |
| Homemaker | 6.59 | 0.09 | 6.40 | 6.78 | . |
| Unemployed and Looking for a Job | 6.13 | 0.24 | 5.67 | 6.60 | . |
| None of These/Other | 5.26 | 0.23 | 4.81 | 5.71 | . |
| Education |  |  |  |  |  |
| Up to 8 Years | 6.74 | 0.47 | 5.76 | 7.72 | 0.00 |
| 9-15 Years | 6.63 | 0.04 | 6.56 | 6.70 | . |
| 16+ Years | 7.16 | 0.02 | 7.12 | 7.19 | . |
| Service Attendance |  |  |  |  |  |
| >1/Week | 7.39 | 0.07 | 7.24 | 7.53 | 0.00 |
| 1/Week | 7.23 | 0.04 | 7.15 | 7.31 | . |
| 1-3/Month | 7.07 | 0.11 | 6.86 | 7.29 | . |
| A Few Times a Year | 6.86 | 0.05 | 6.77 | 6.95 | . |
| Never | 6.52 | 0.04 | 6.44 | 6.59 | . |
| Immigration Status |  |  |  |  |  |
| Born in This Country | 6.79 | 0.03 | 6.74 | 6.84 | 0.09 |
| Born in Another Country | 6.99 | 0.12 | 6.76 | 7.22 | . |
| Religion |  |  |  |  |  |
| Christianity | 6.97 | 0.03 | 6.92 | 7.03 | 0.00 |
| Islam | 6.37 | 0.42 | 5.52 | 7.21 | . |
| Hinduism | 7.38 | 0.18 | 7.02 | 7.73 | . |
| Buddhism | 6.99 | 0.23 | 6.53 | 7.45 | . |
| Judaism | 7.23 | 0.11 | 7.00 | 7.45 | . |
| Sikhism | 6.49 | 0.59 | 5.20 | 7.77 | . |
| Baha'i | 5.63 | 1.31 | 2.87 | 8.40 | . |
| Jainism | 5.29 | 0.96 | 3.00 | 7.58 | . |
| Shinto | 7.78 | 0.67 | 6.24 | 9.31 | . |
| Taoism | 6.22 | 0.45 | 5.31 | 7.12 | . |
| Confucianism | 8.13 | 0.88 | 5.59 | 10.66 | . |
| Primal, Animist, or Folk Religion | 6.59 | 0.77 | 5.06 | 8.12 | . |
| Spiritism | . | . | . | . | . |
| African-Derived | . | . | . | . | . |
| Chinese | . | . | . | . | . |
| Some Other Religion | 6.17 | 0.23 | 5.73 | 6.62 | . |
| No Religion/Atheist/Agnostic | 6.53 | 0.05 | 6.44 | 6.62 | . |
| Race/Ethnicity |  |  |  |  |  |
| White | 6.85 | 0.02 | 6.81 | 6.90 | 0.01 |
| Other | 6.58 | 0.11 | 6.36 | 6.80 | . |
| Black | 6.90 | 0.08 | 6.73 | 7.06 | . |
| Asian | 6.89 | 0.10 | 6.69 | 7.09 | . |
| Hispanic | 6.58 | 0.10 | 6.39 | 6.77 | . |
| Other | . | . | . | . | . |

**Table S23. Population weighted meta-analysis of results demographic group means.**

| Variable | Category | Est | 95% CI | SE |
| --- | --- | --- | --- | --- |
| Age group |  |  |  |  |
|  | 18-24 | 7.30 | (7.18,7.43) | 0.061 |
|  | 25-29 | 7.16 | (7.04,7.29) | 0.065 |
|  | 30-39 | 7.07 | (6.99,7.15) | 0.041 |
|  | 40-49 | 6.93 | (6.84,7.02) | 0.046 |
|  | 50-59 | 6.83 | (6.70,6.95) | 0.063 |
|  | 60-69 | 6.53 | (6.35,6.71) | 0.092 |
|  | 70-79 | 6.44 | (6.20,6.69) | 0.127 |
|  | 80 or older | 6.41 | (5.71,7.11) | 0.357 |
| Gender |  |  |  |  |
|  | Male | 7.12 | (7.06,7.18) | 0.031 |
|  | Female | 6.90 | (6.83,6.96) | 0.031 |
|  | Other | 6.69 | (6.06,7.33) | 0.325 |
| Marital status |  |  |  |  |
|  | Married | 7.04 | (6.96,7.13) | 0.044 |
|  | Separated | 6.27 | (5.61,6.93) | 0.337 |
|  | Divorced | 6.67 | (6.44,6.91) | 0.120 |
|  | Widowed | 6.33 | (6.08,6.58) | 0.127 |
|  | Domestic partner | 6.94 | (6.74,7.15) | 0.105 |
|  | Single, never married | 6.87 | (6.80,6.94) | 0.036 |
| Employment status |  |  |  |  |
|  | Employed for an employer | 7.21 | (7.15,7.27) | 0.030 |
|  | Self-employed | 7.28 | (7.14,7.41) | 0.070 |
|  | Retired | 6.43 | (6.25,6.61) | 0.092 |
|  | Student | 7.21 | (7.02,7.39) | 0.094 |
|  | Homemaker | 6.54 | (6.32,6.75) | 0.110 |
|  | Unemployed and looking for a job | 6.50 | (6.29,6.70) | 0.105 |
|  | None of these/other | 5.53 | (5.06,6.00) | 0.241 |
| Education |  |  |  |  |
|  | Up to 8 years | 6.69 | (6.46,6.93) | 0.119 |
|  | 9-15 years | 7.06 | (7.01,7.11) | 0.024 |
|  | 16+ years | 7.34 | (7.25,7.42) | 0.044 |
| Religious service attendance |  |  |  |  |
|  | >1/week | 7.69 | (7.57,7.81) | 0.061 |
|  | 1/week | 7.39 | (7.30,7.48) | 0.045 |
|  | 1-3/month | 7.24 | (7.12,7.36) | 0.062 |
|  | A few times a year | 6.95 | (6.86,7.04) | 0.047 |
|  | Never | 6.72 | (6.62,6.82) | 0.052 |
| Immigration status |  |  |  |  |
|  | Born in this country | 7.00 | (6.96,7.05) | 0.023 |
|  | Born in another country | 6.90 | (6.68,7.11) | 0.110 |


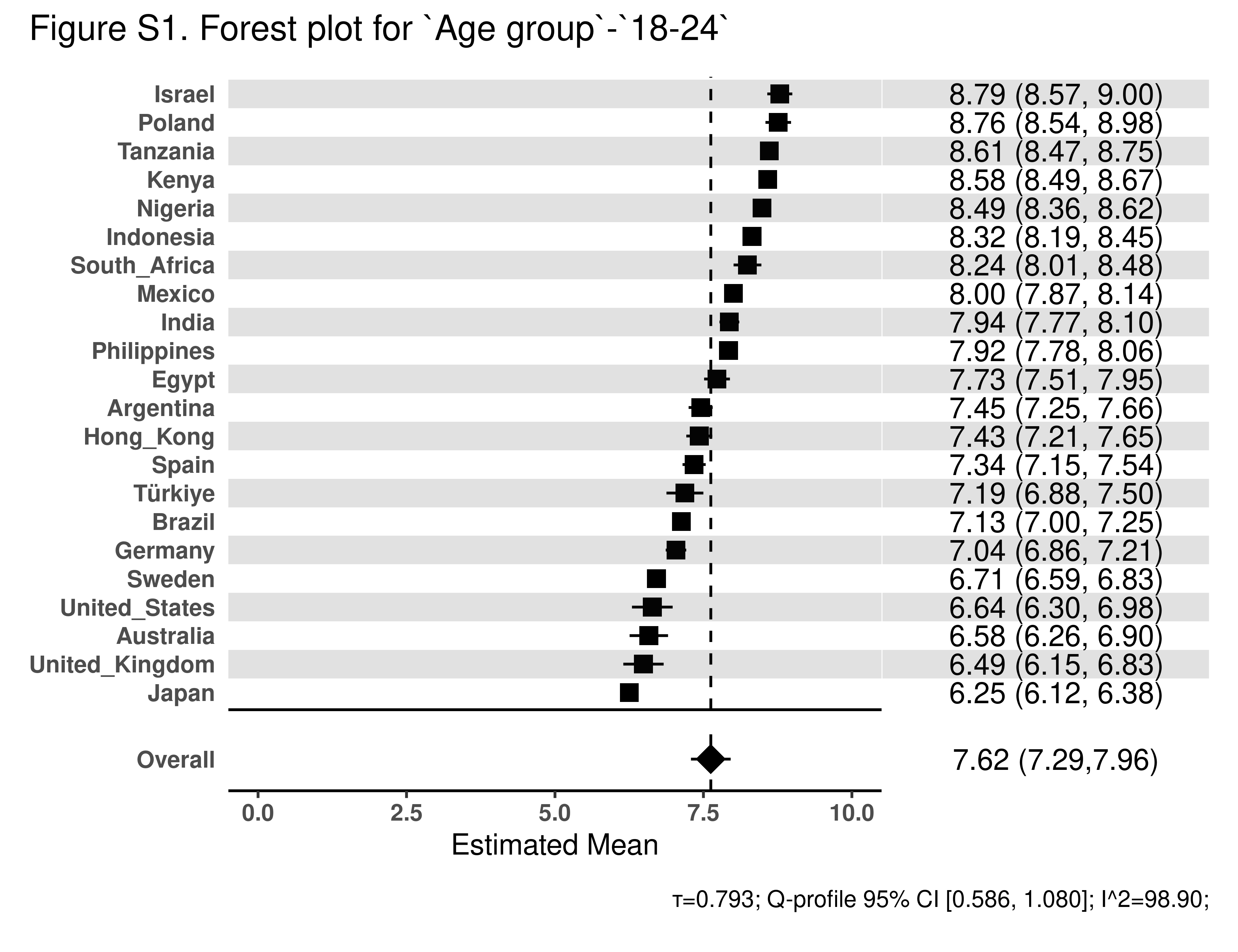


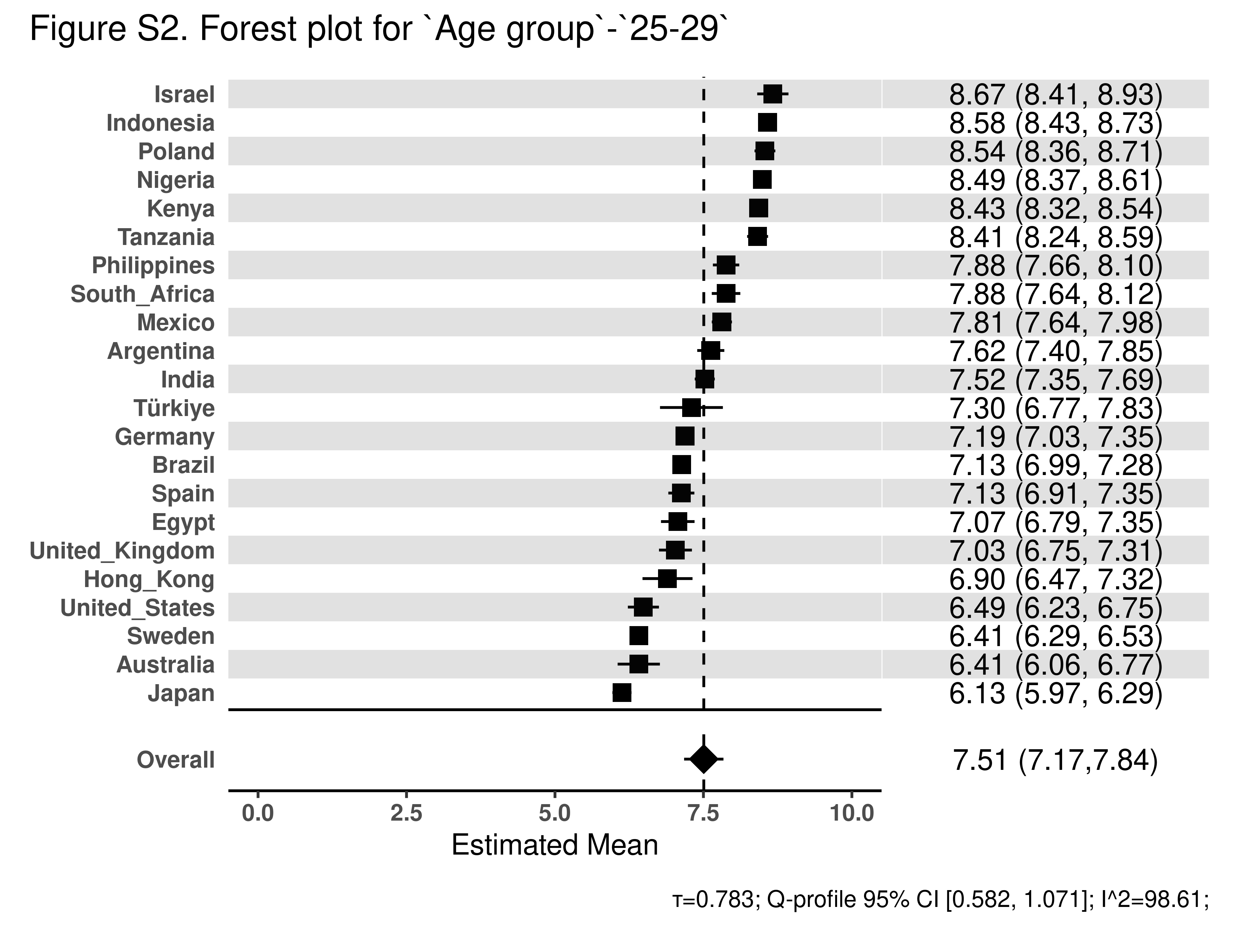


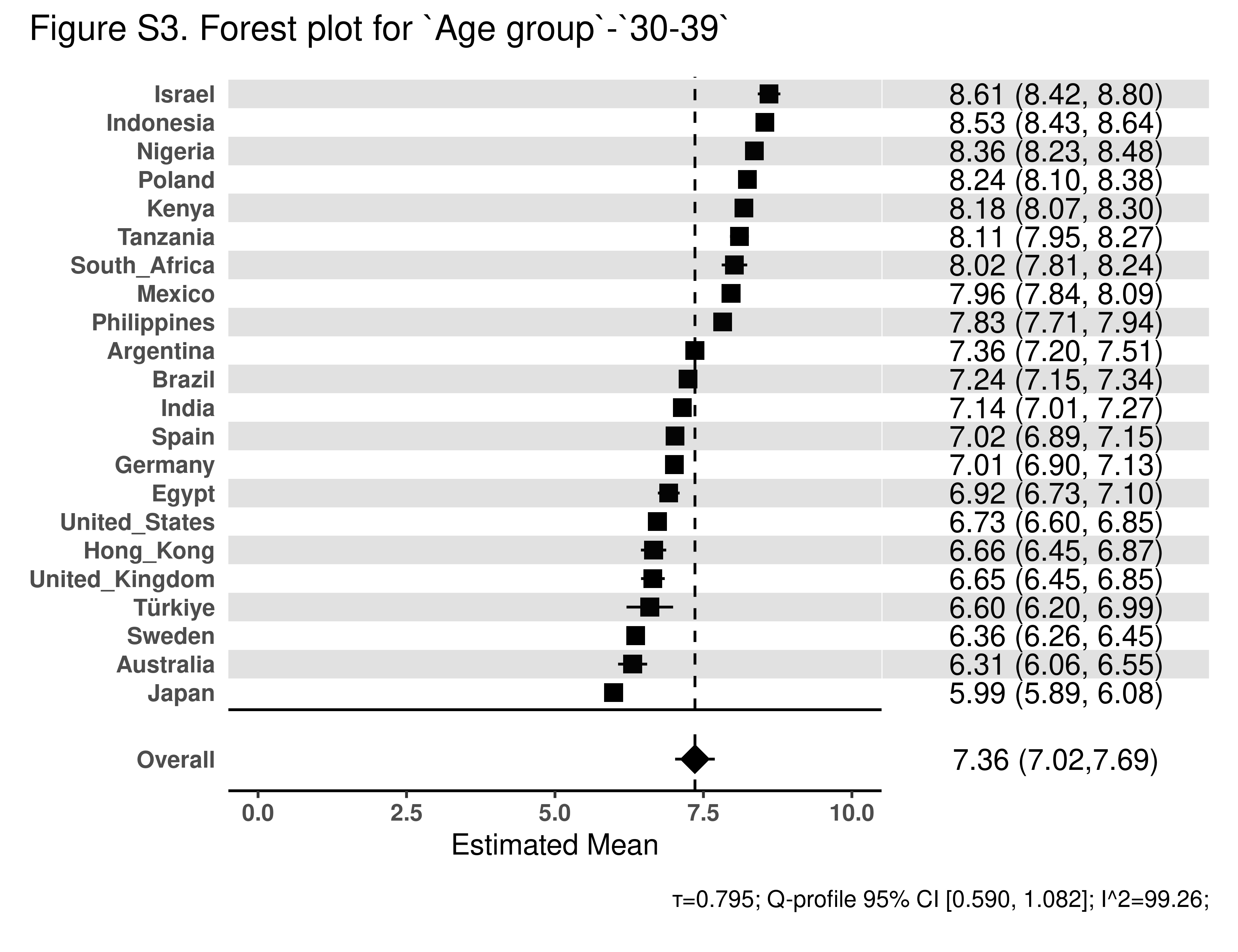


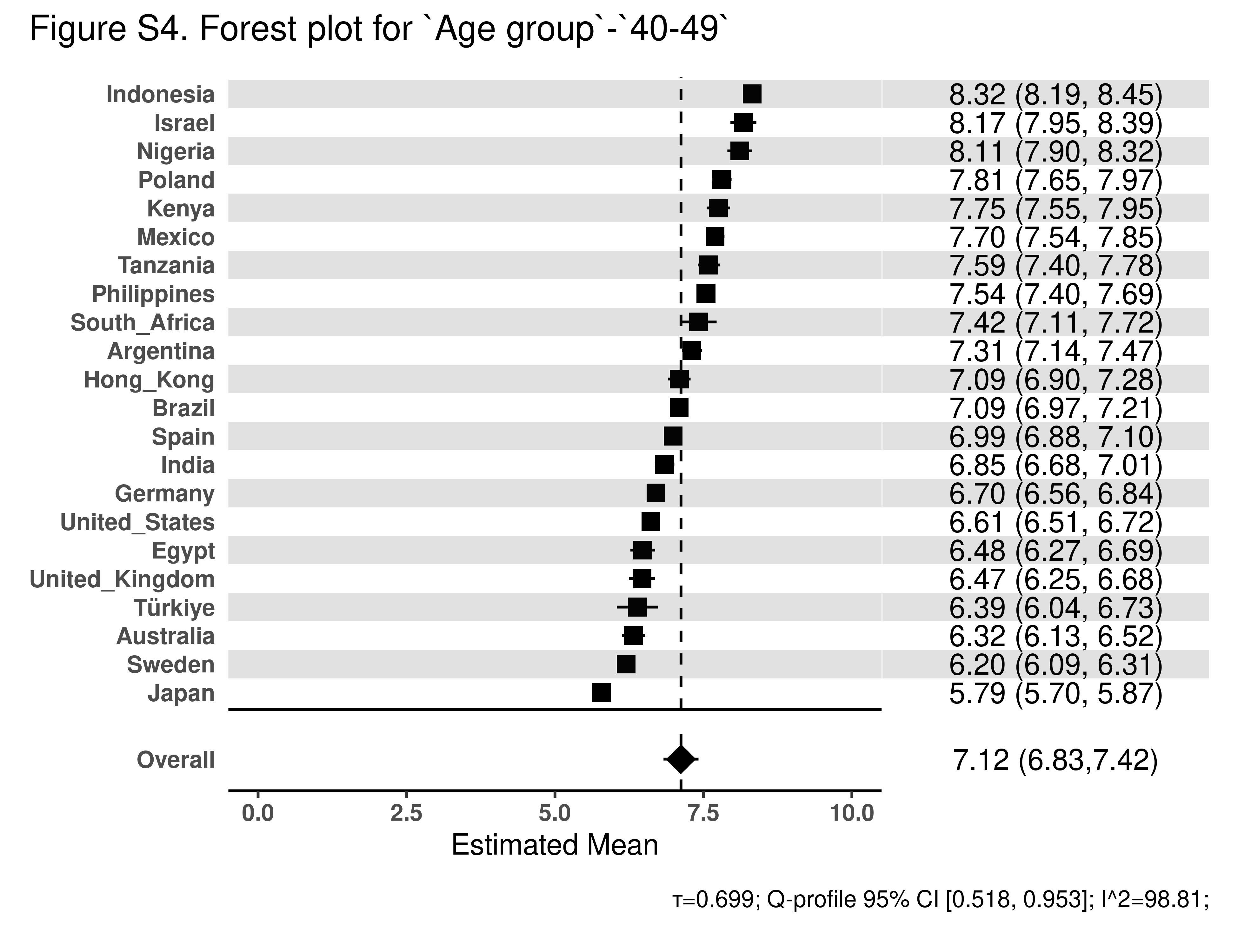


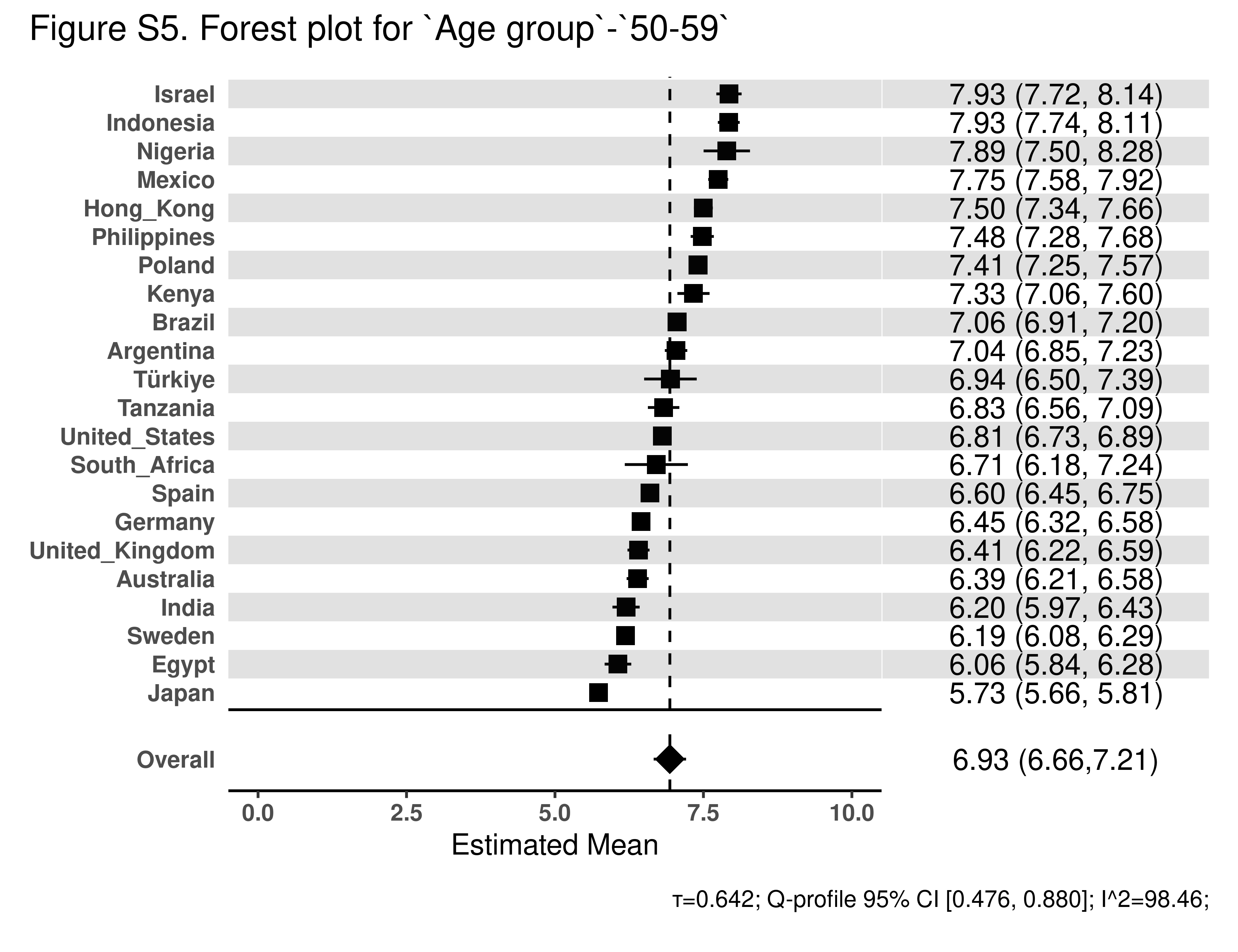


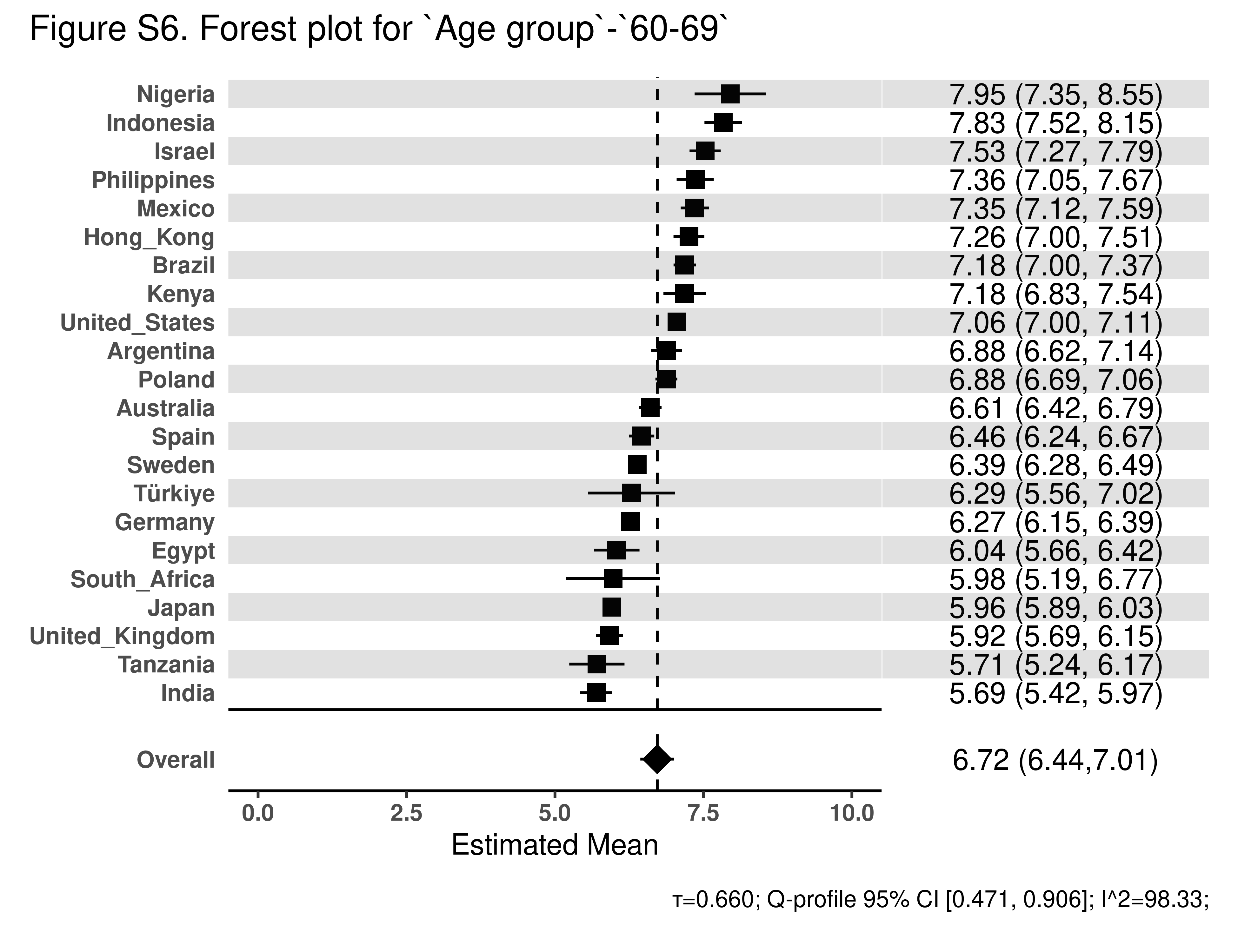


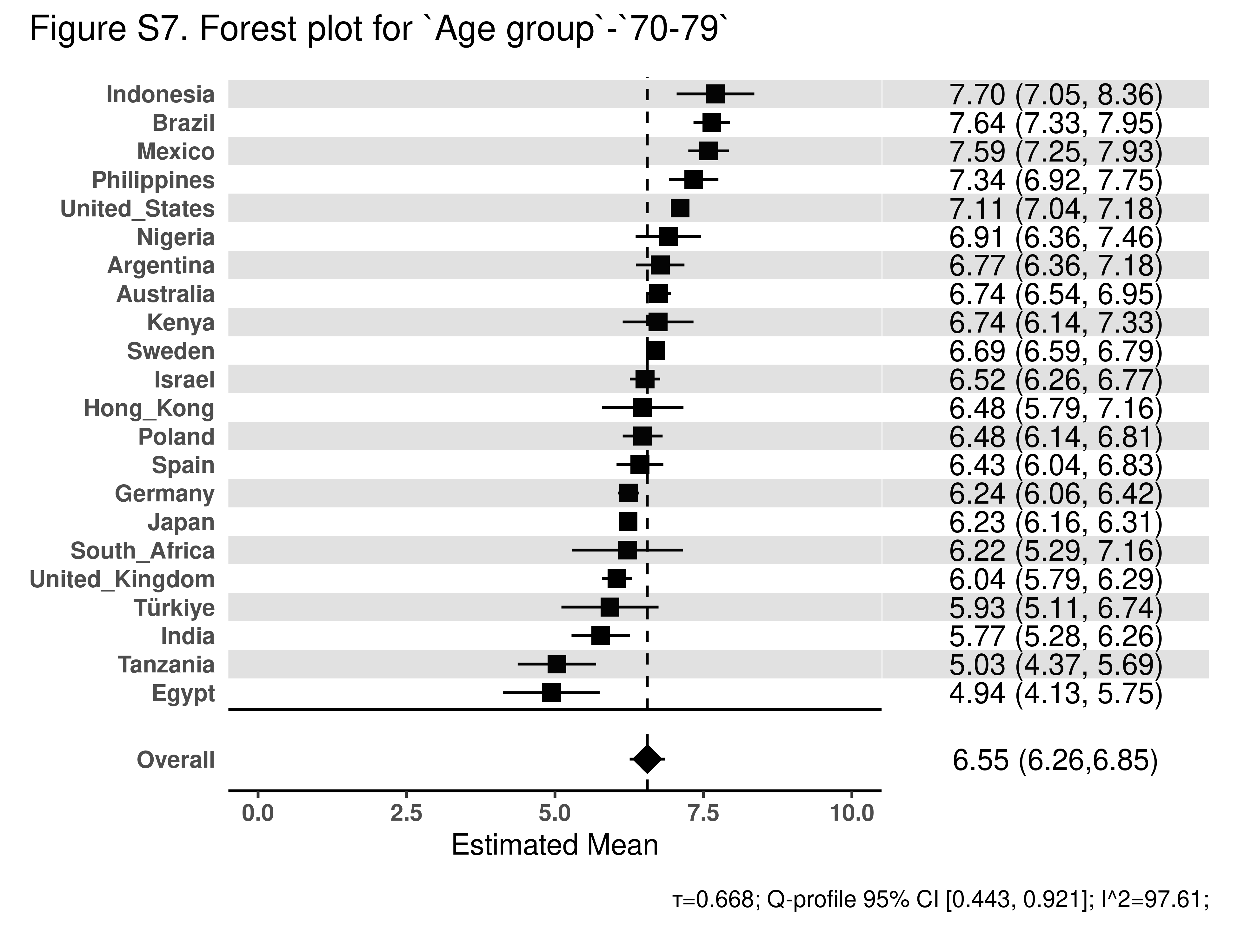


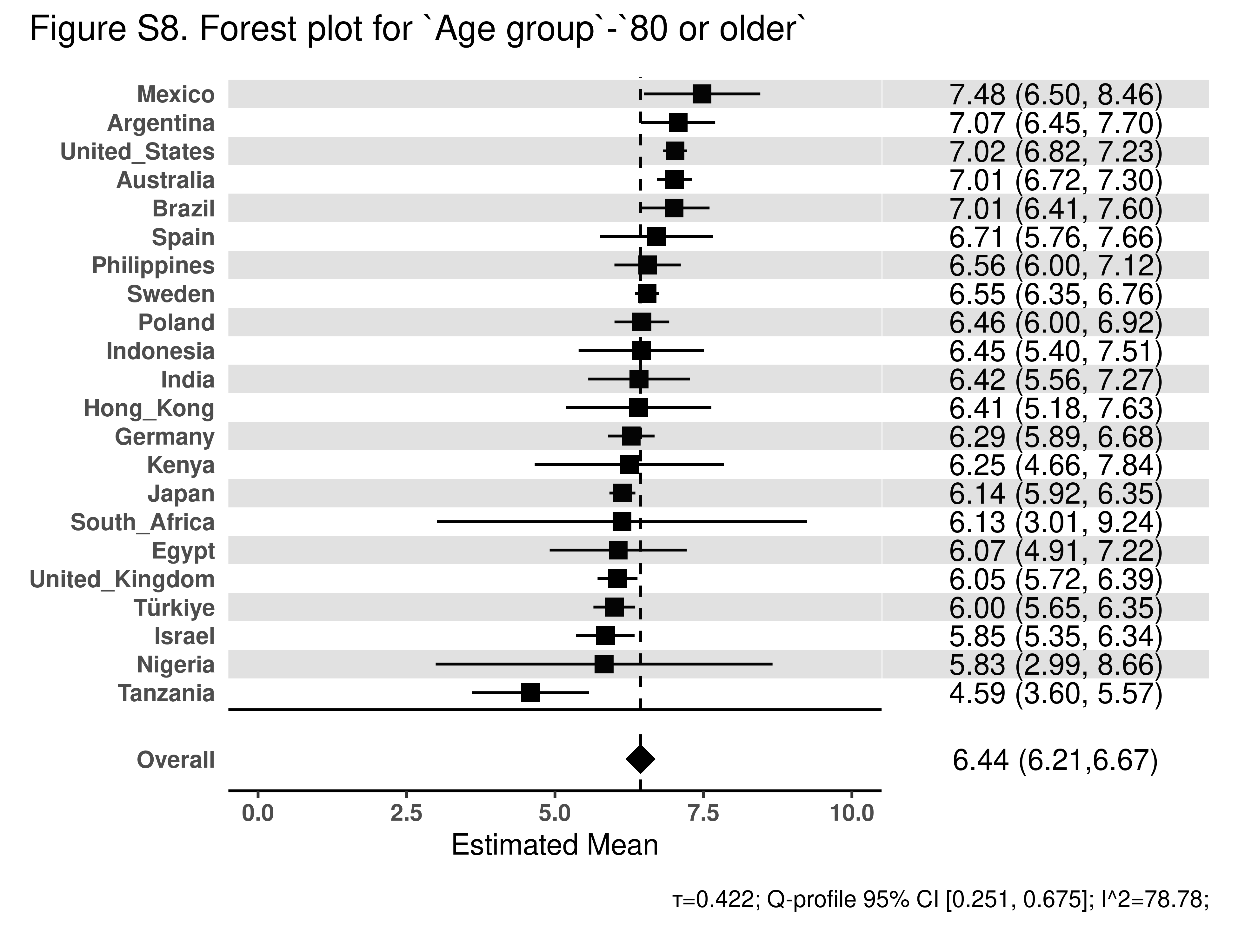


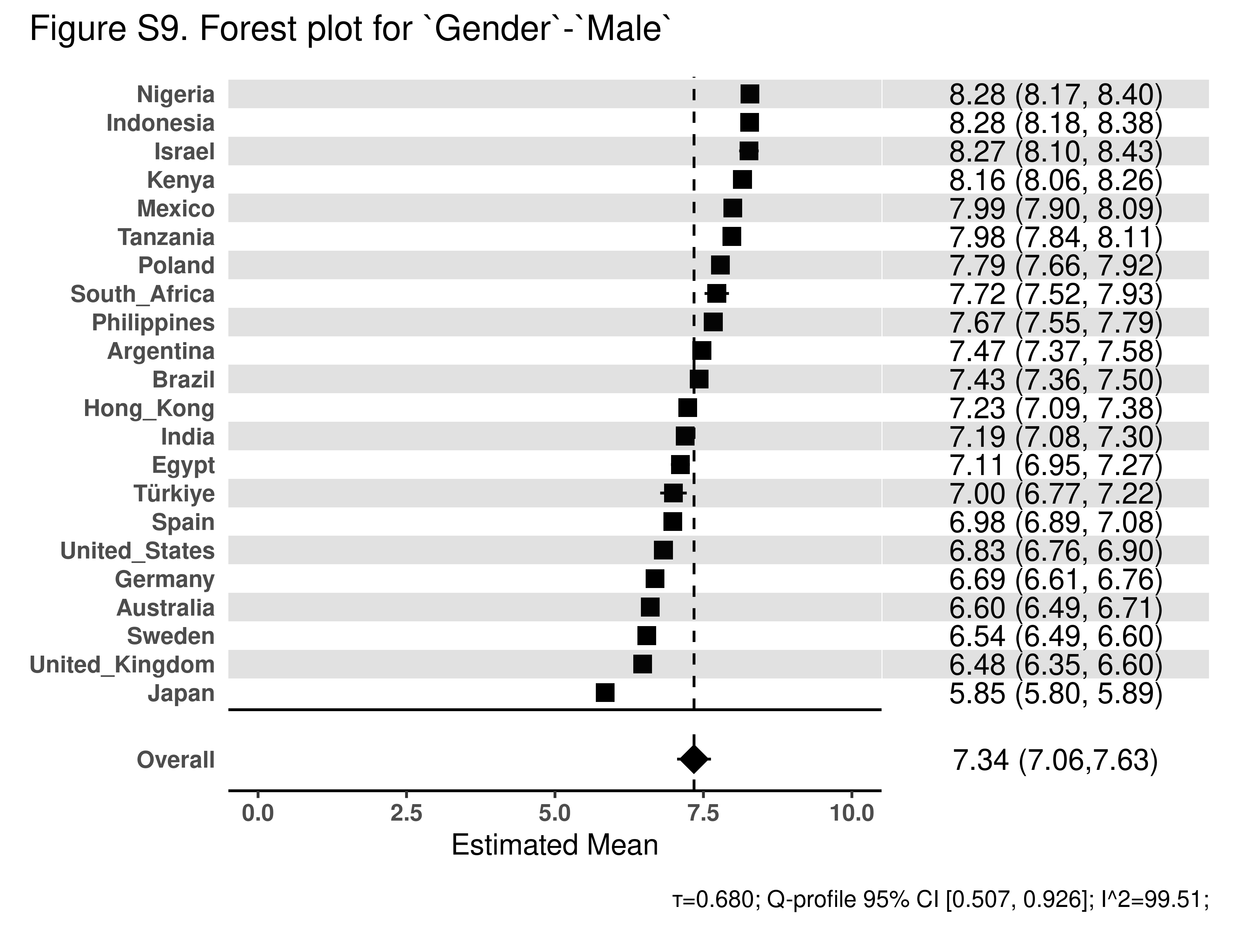


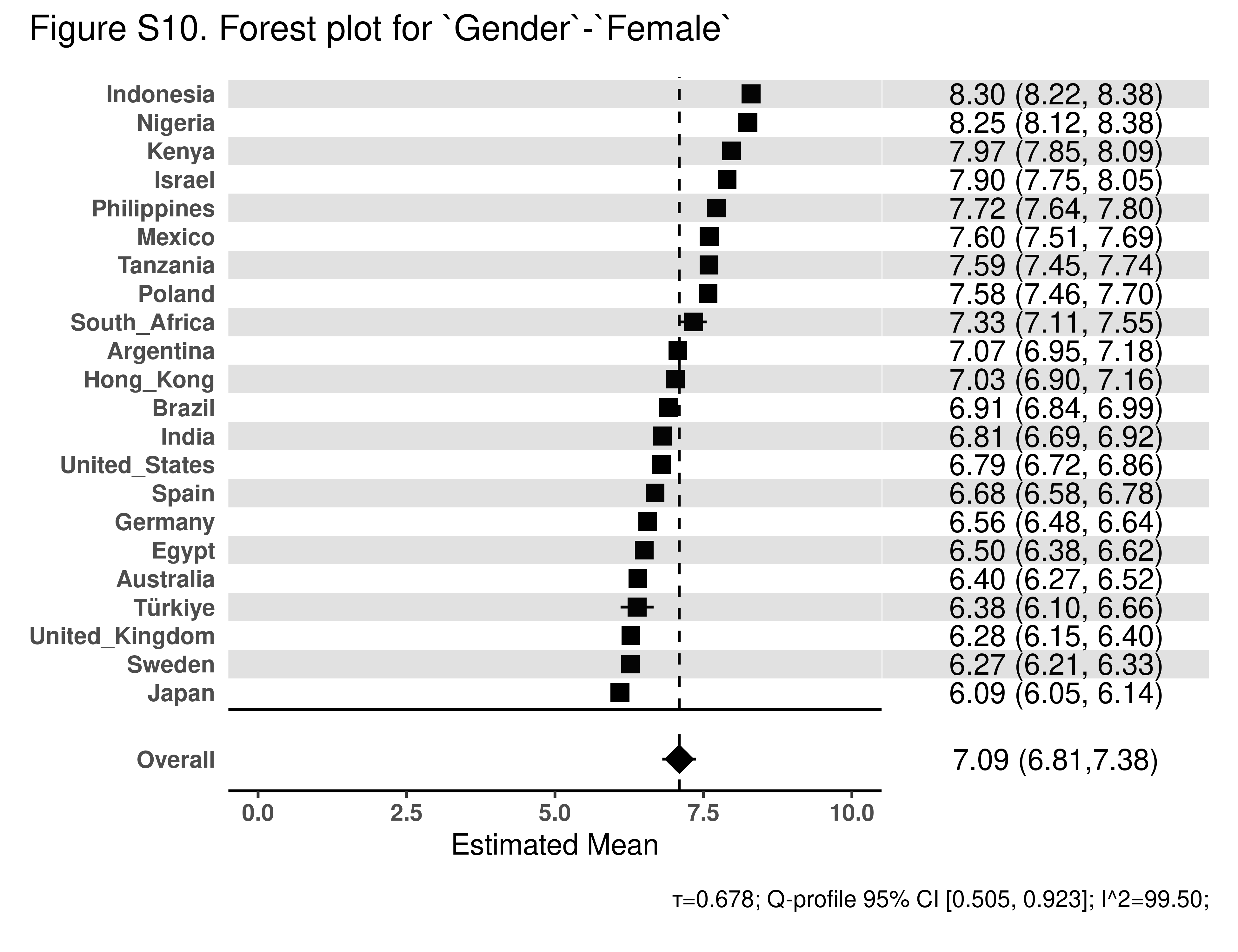


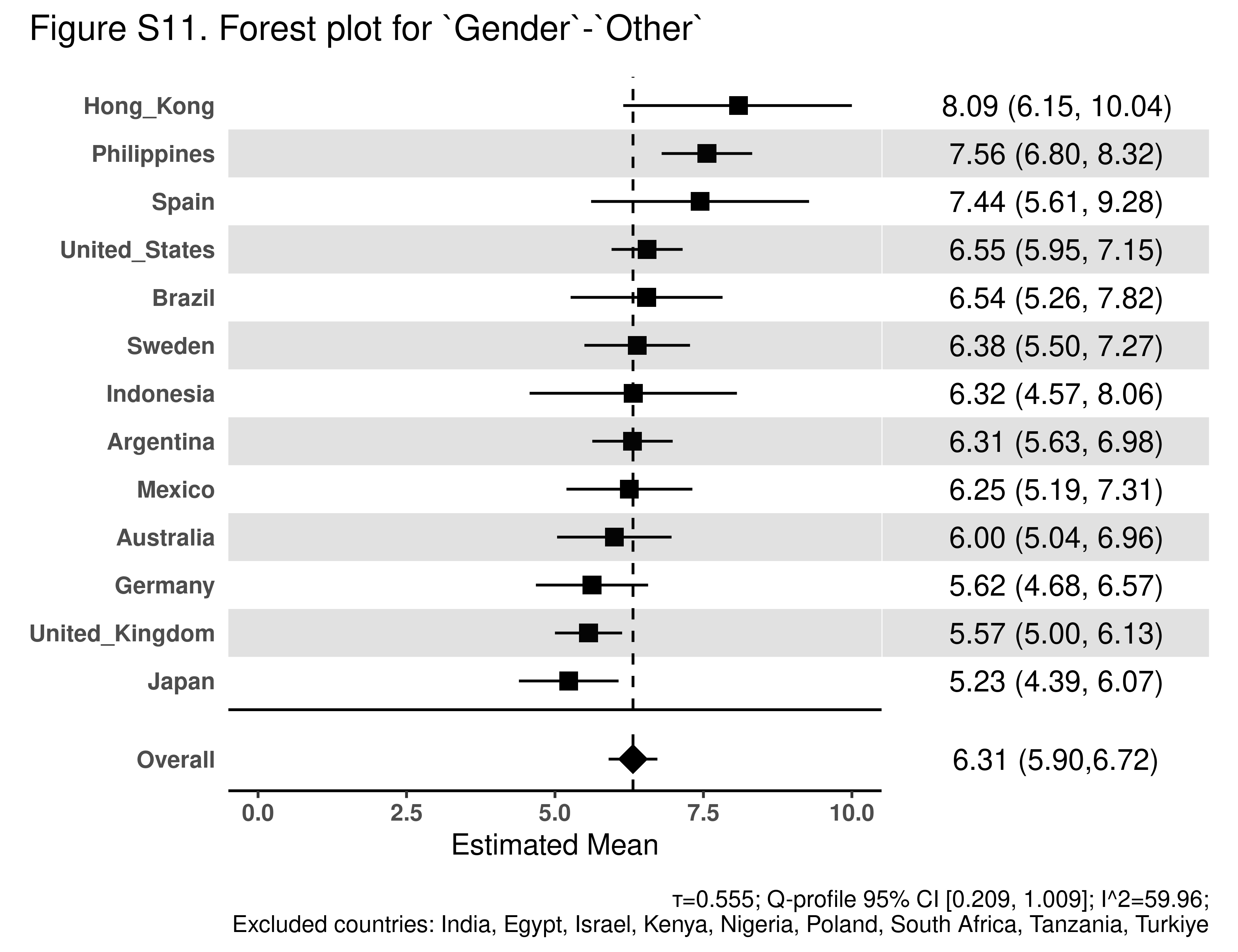


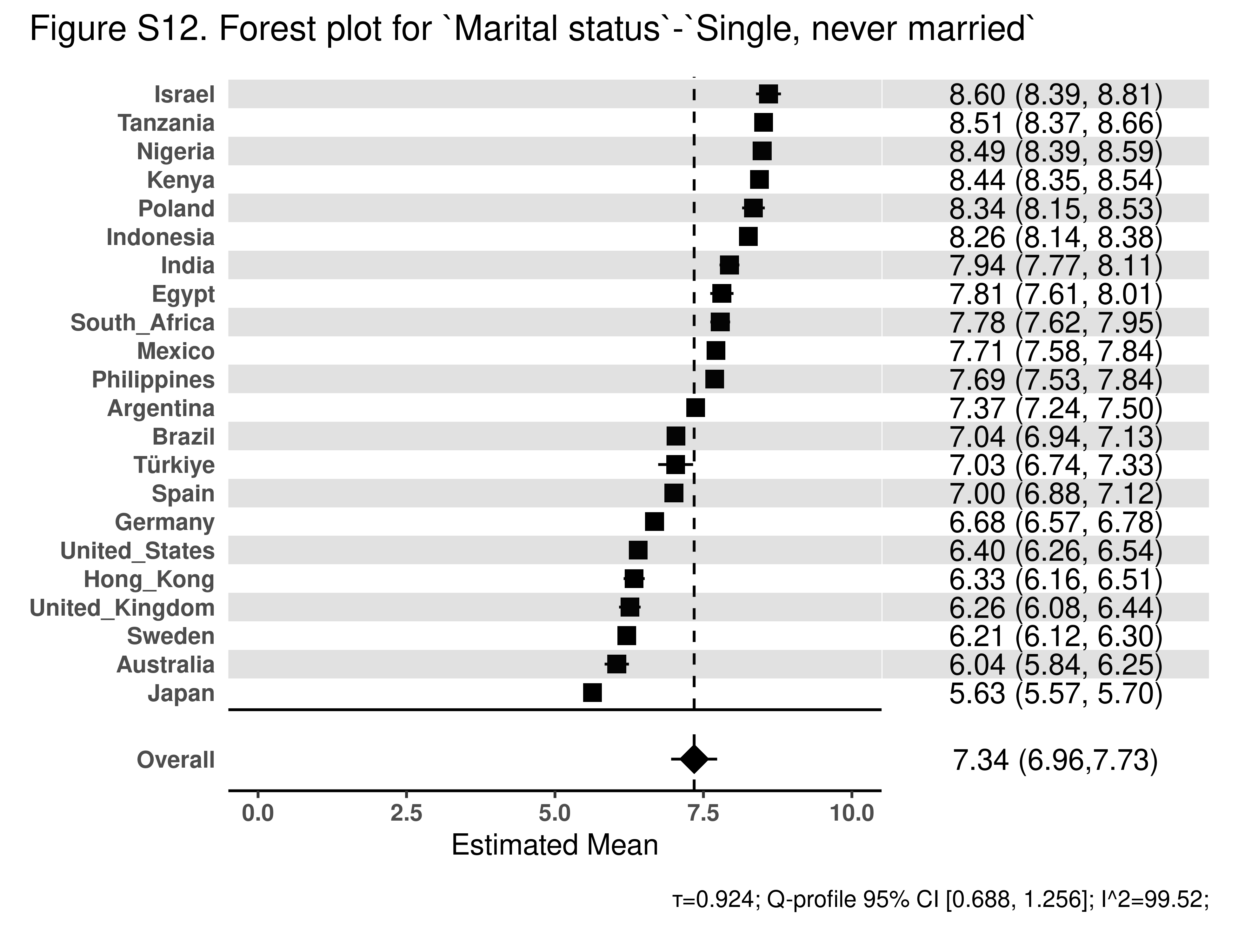


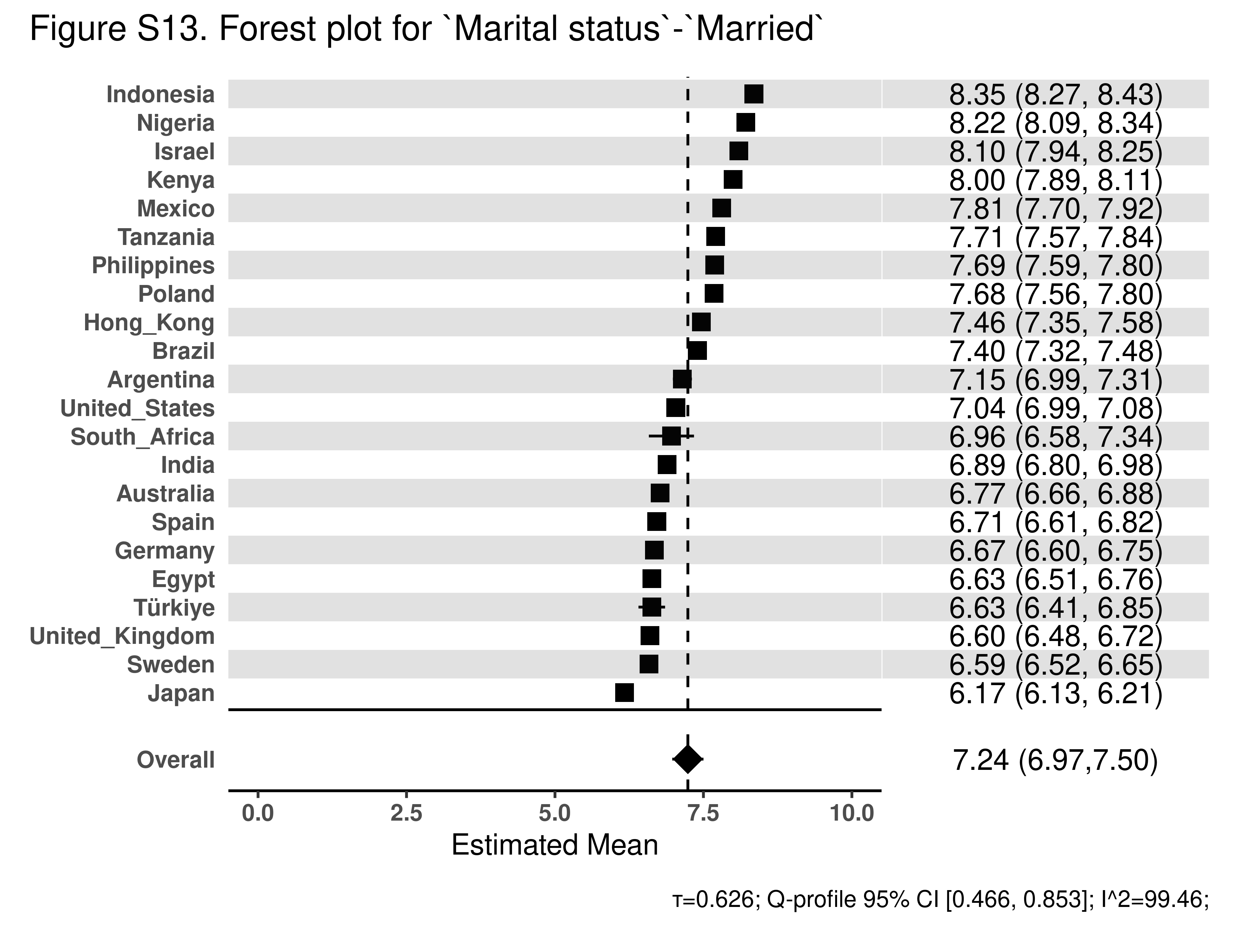


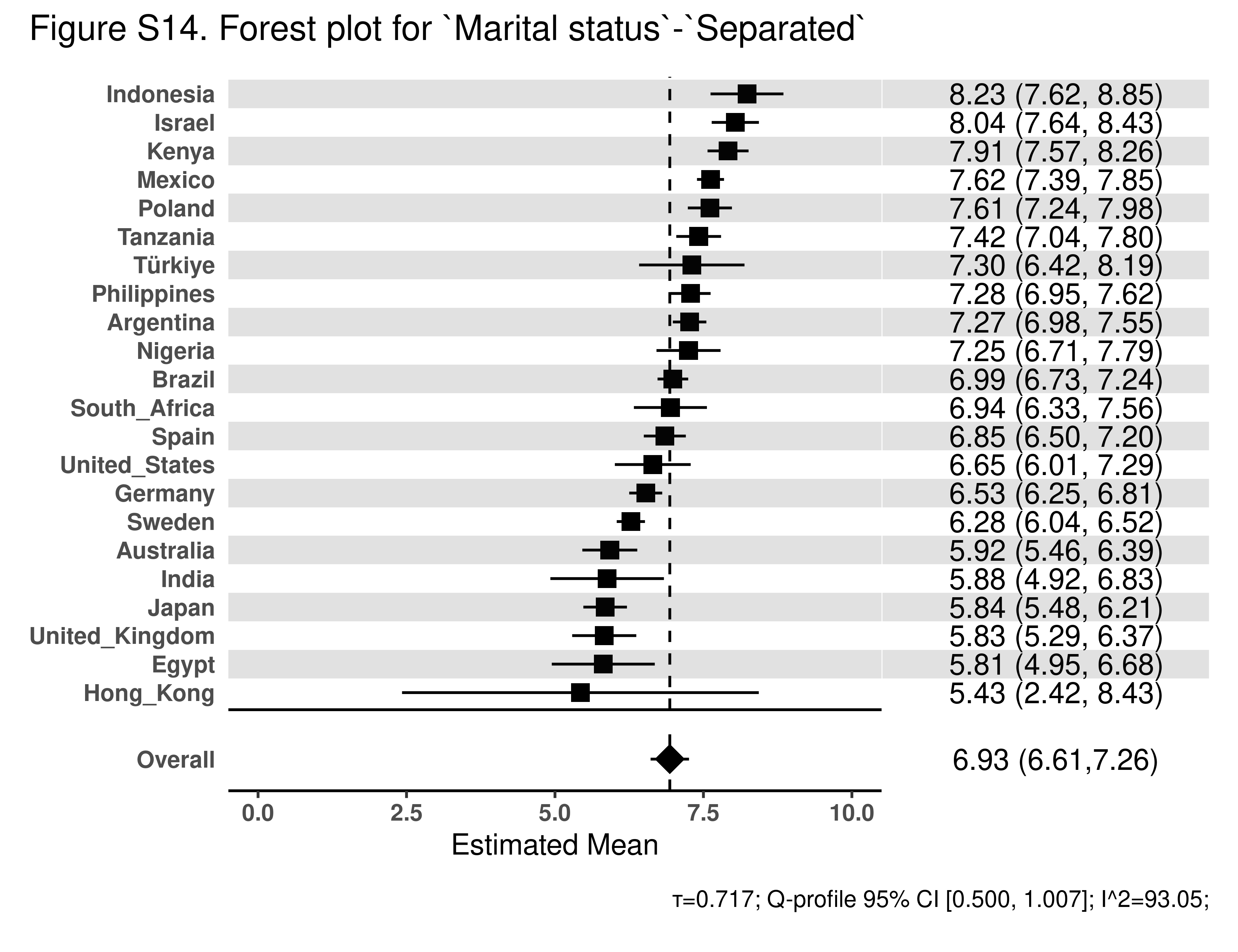


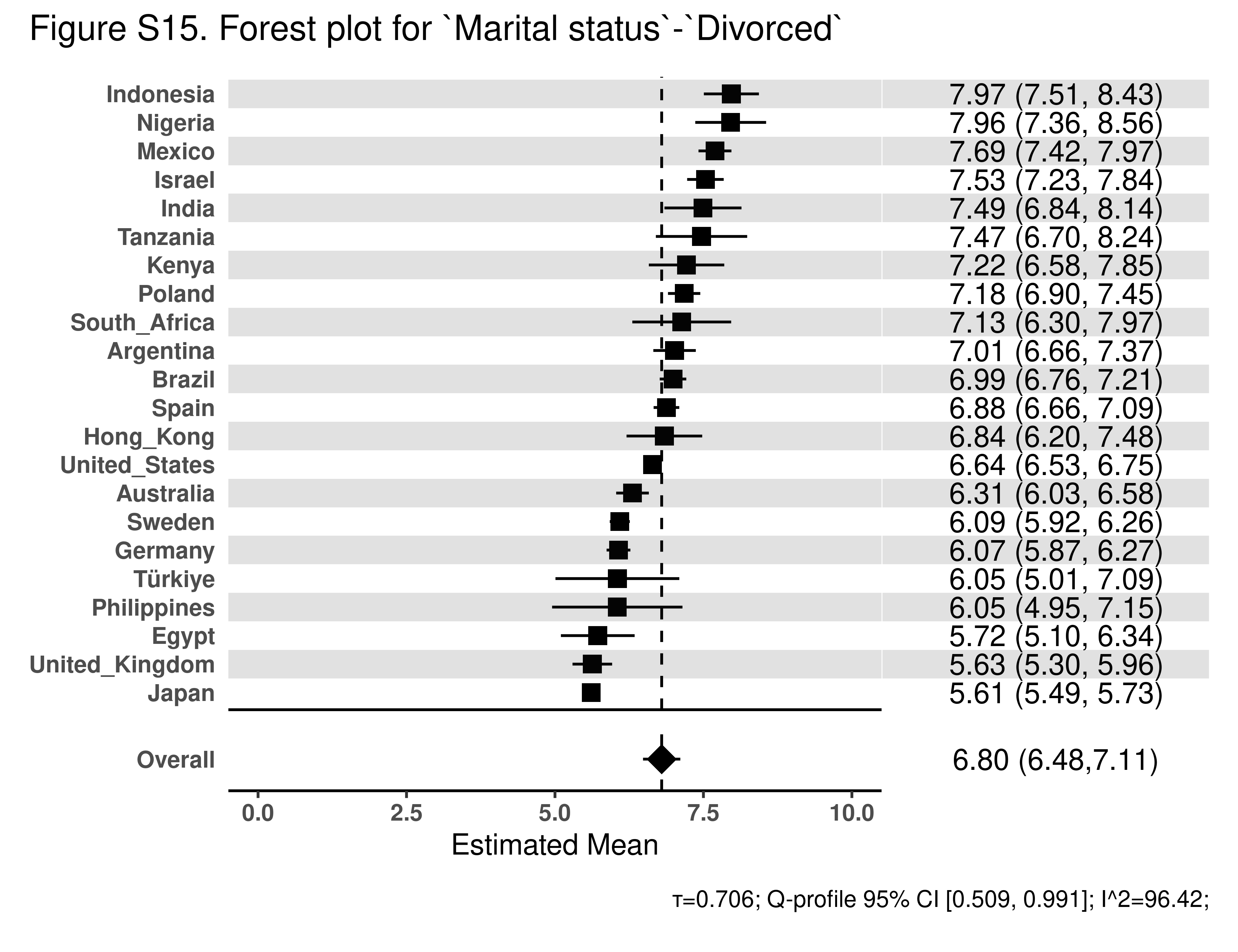


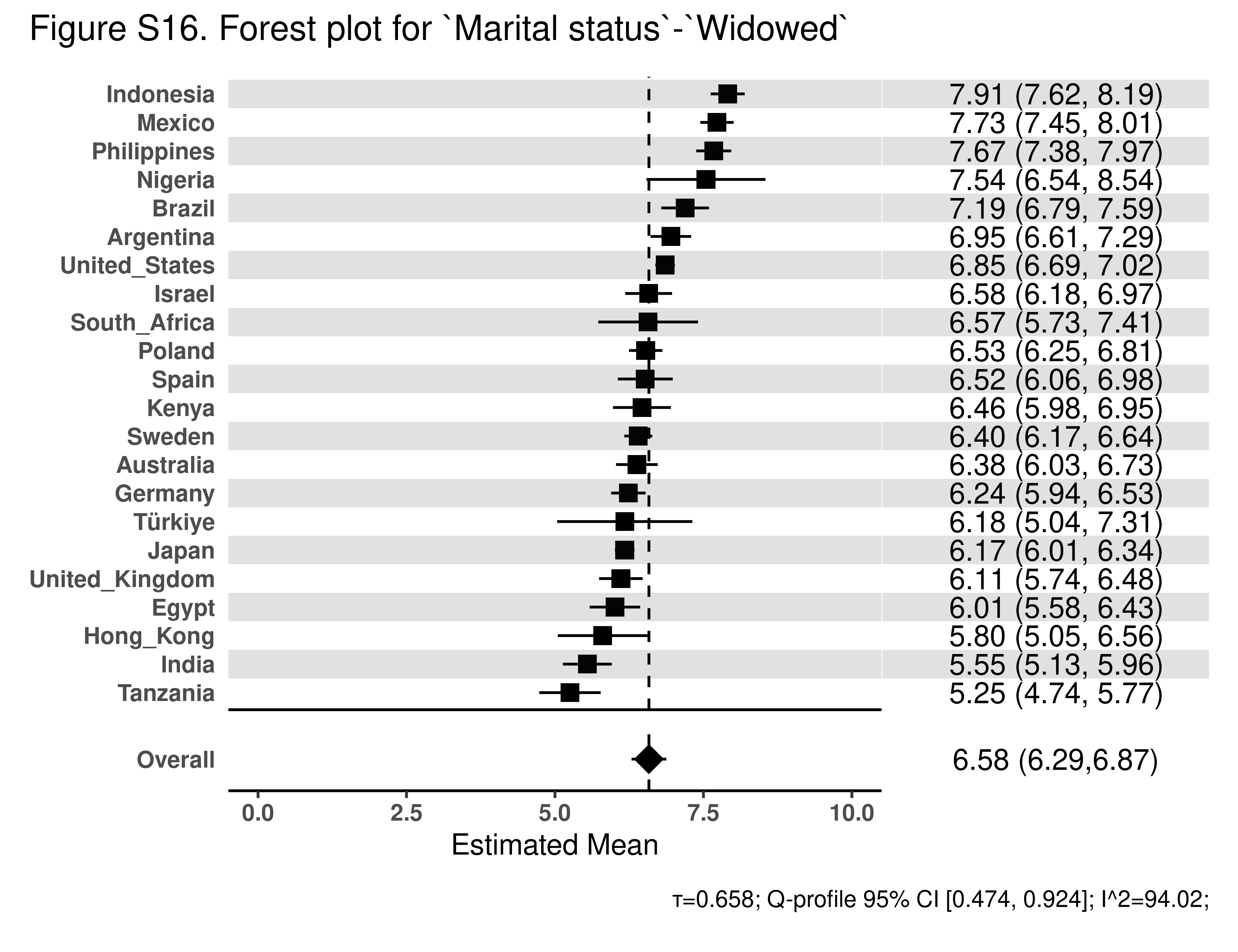


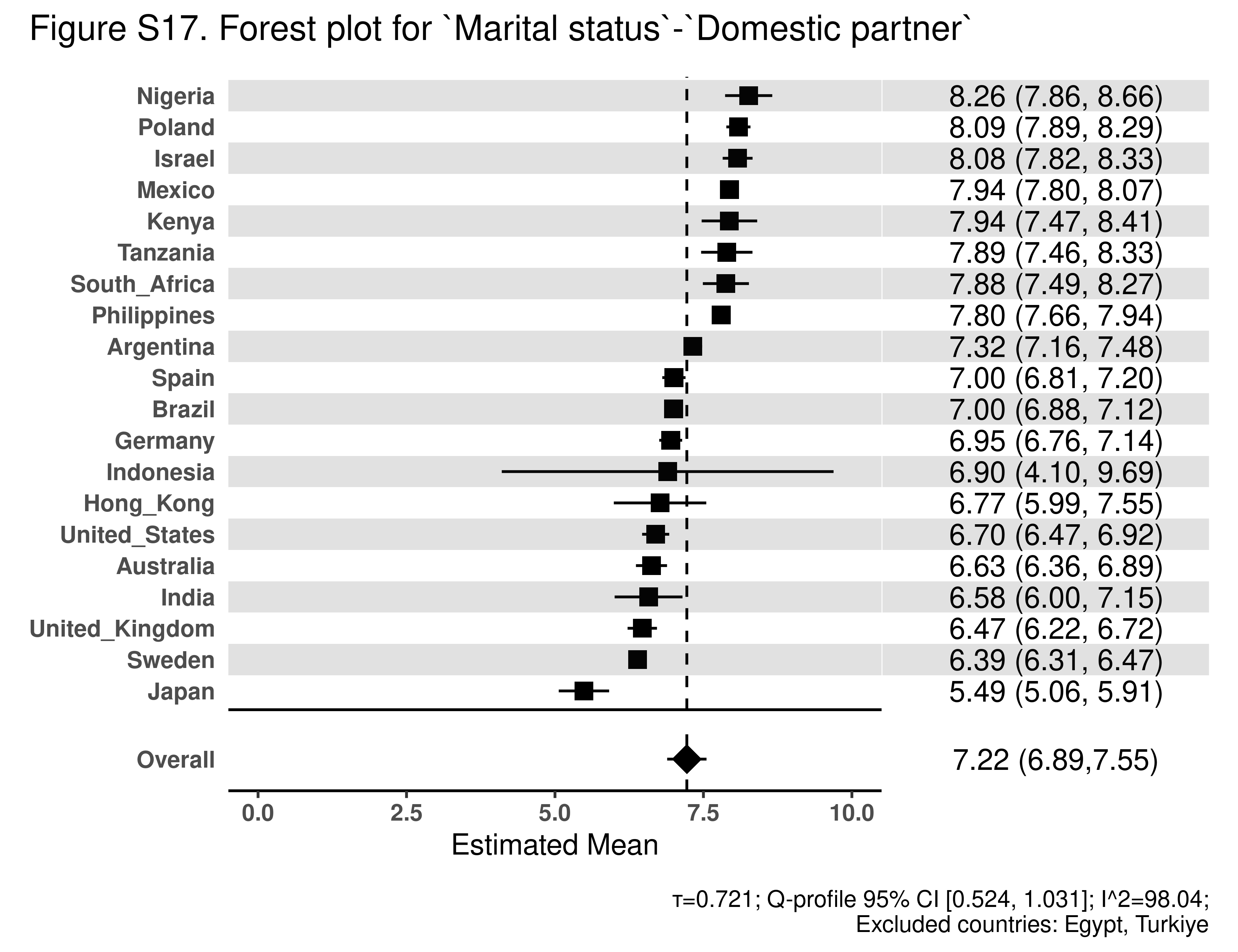


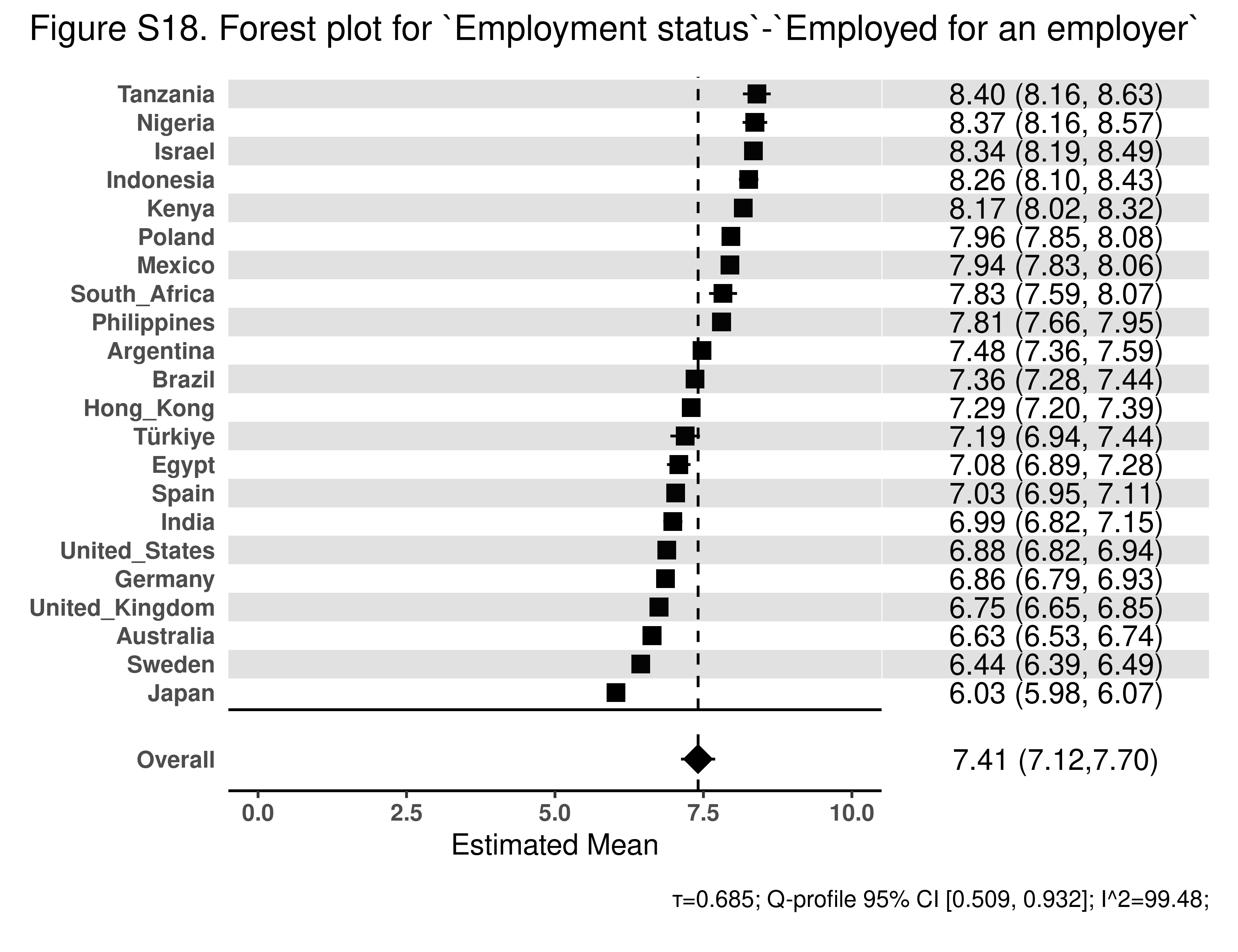


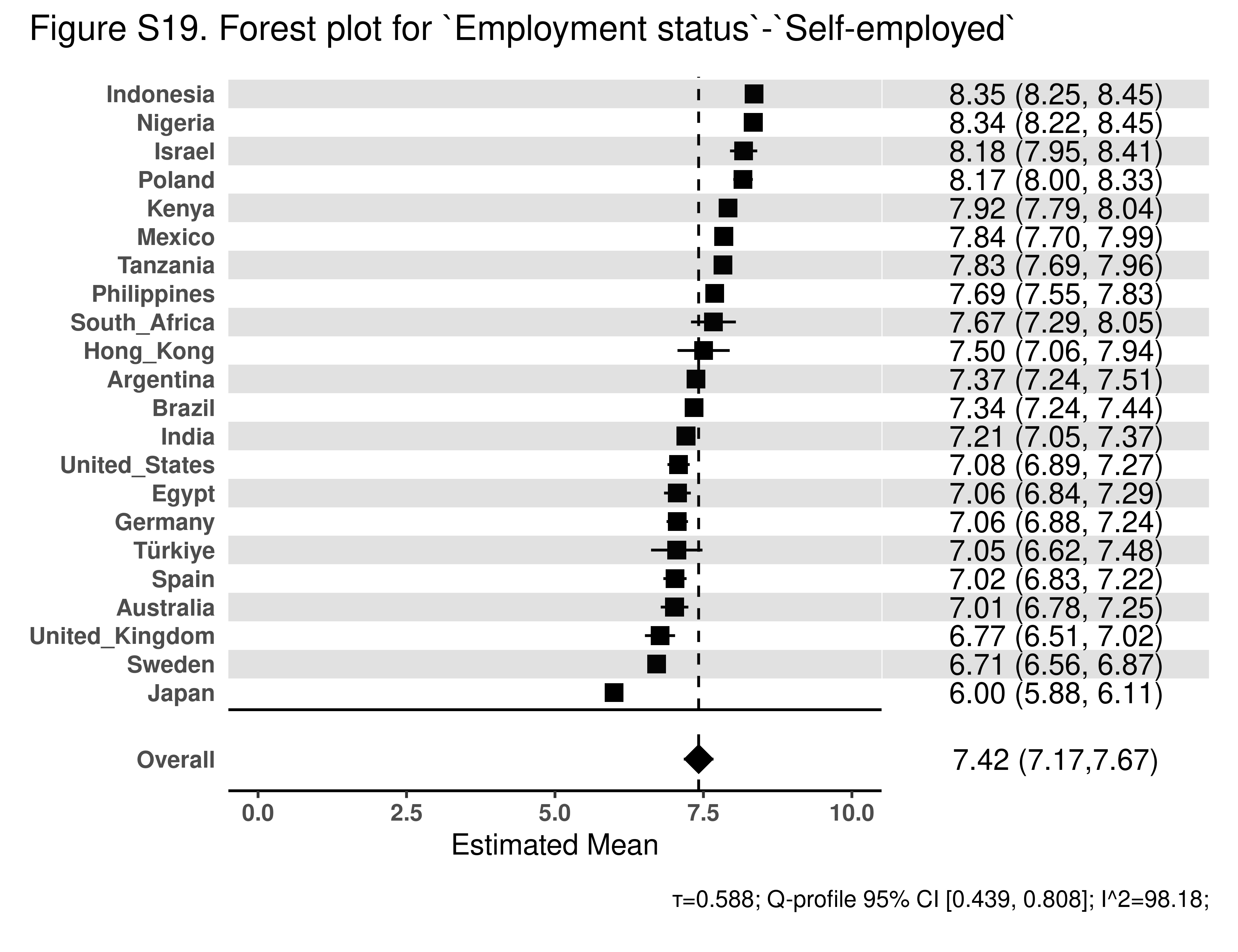


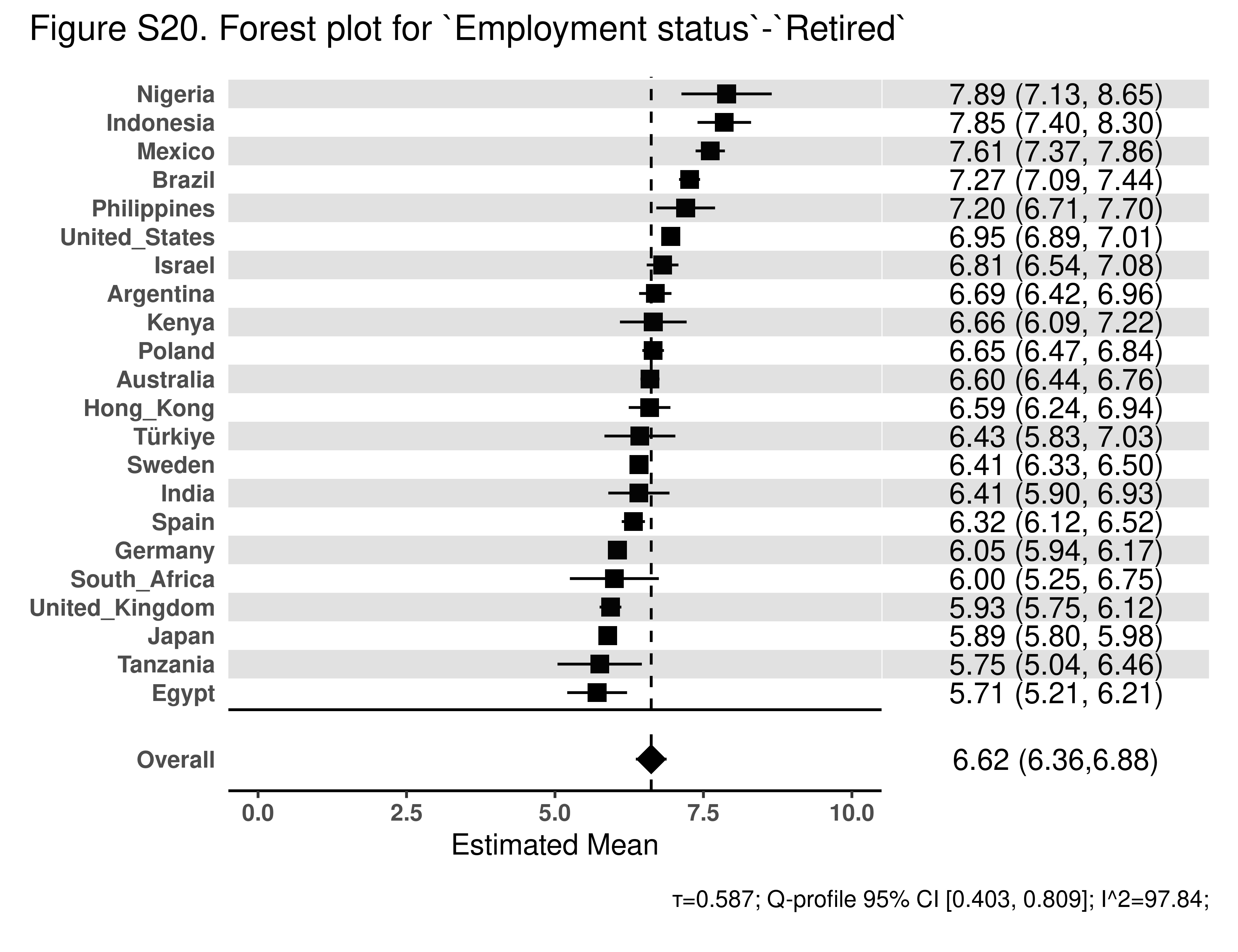


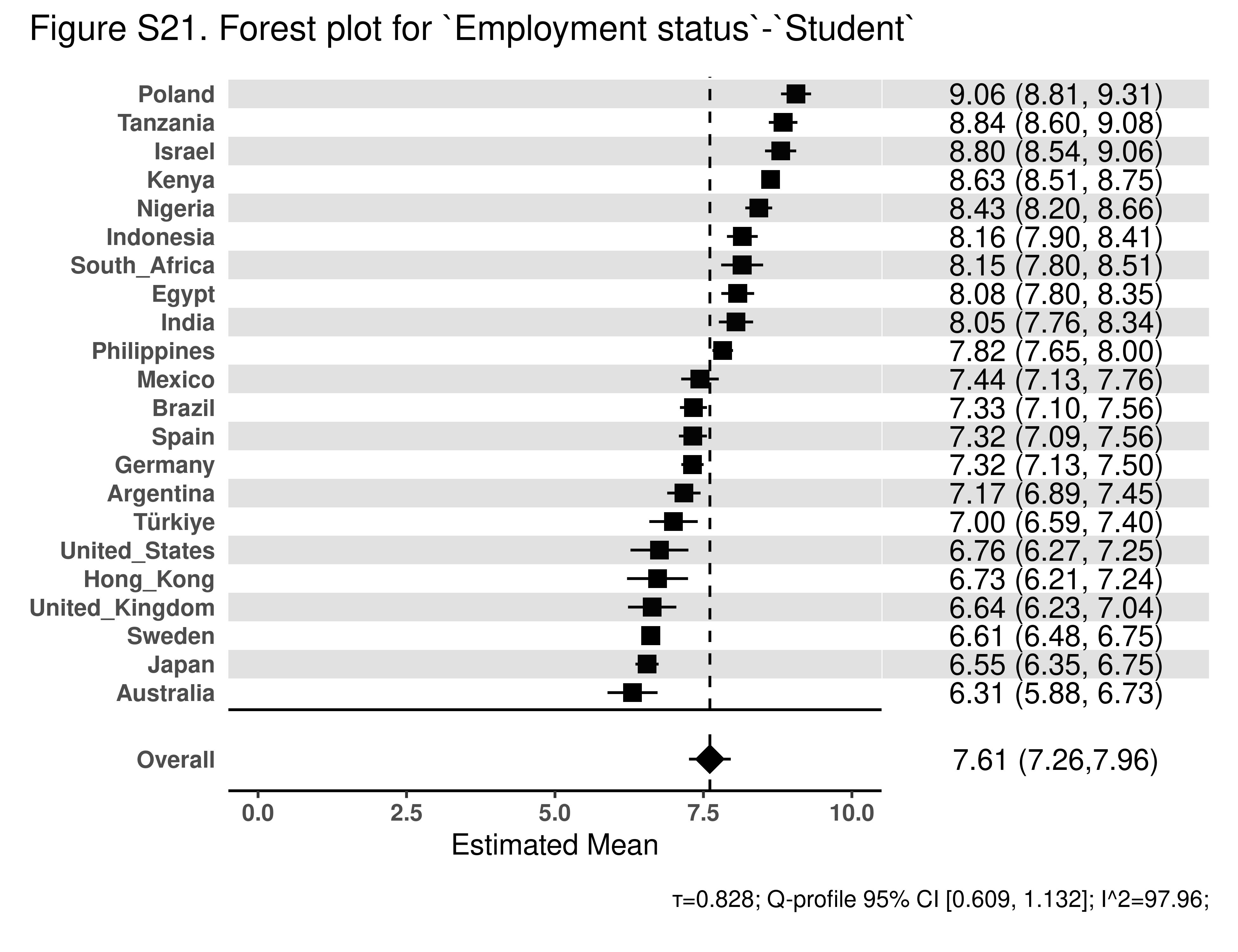


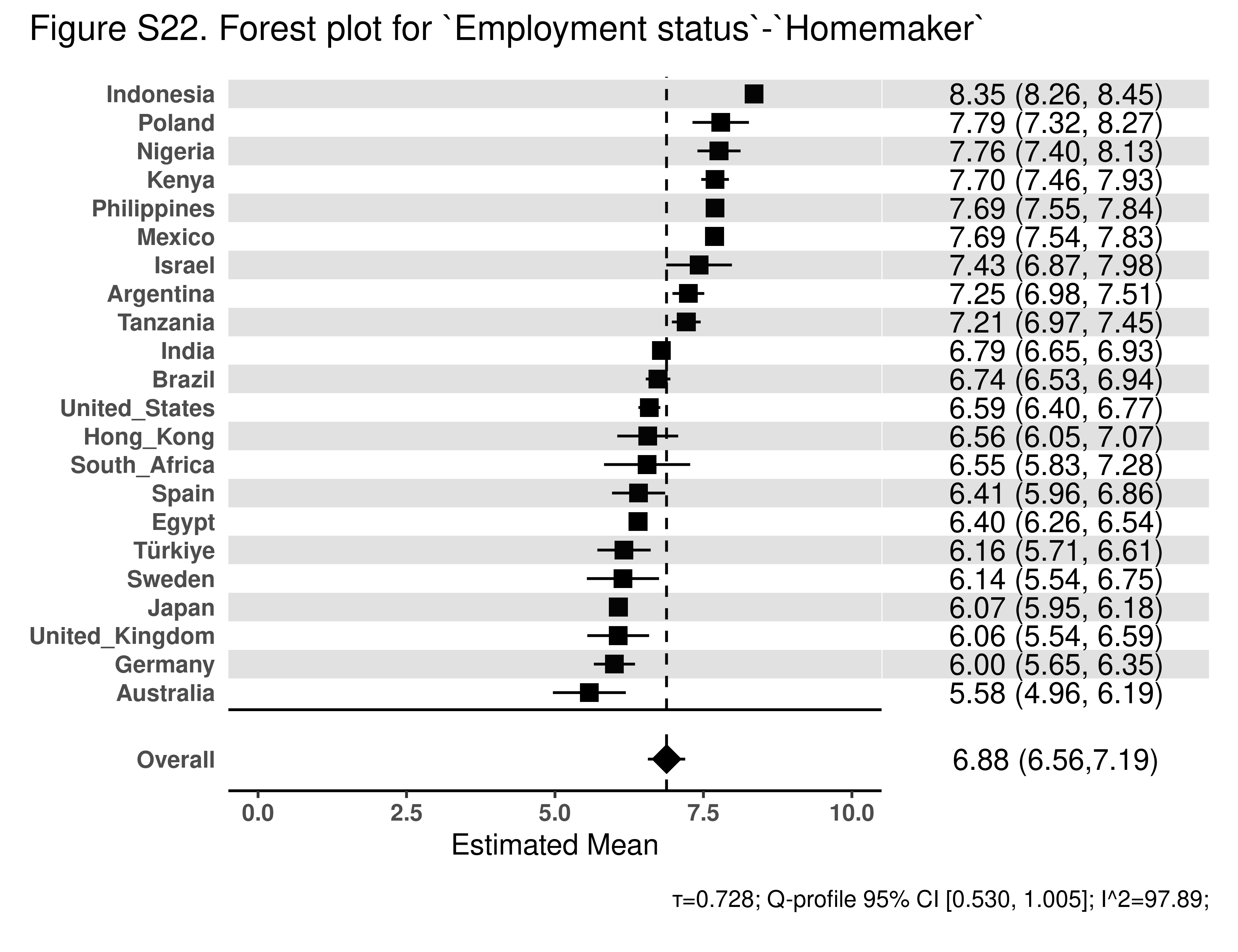


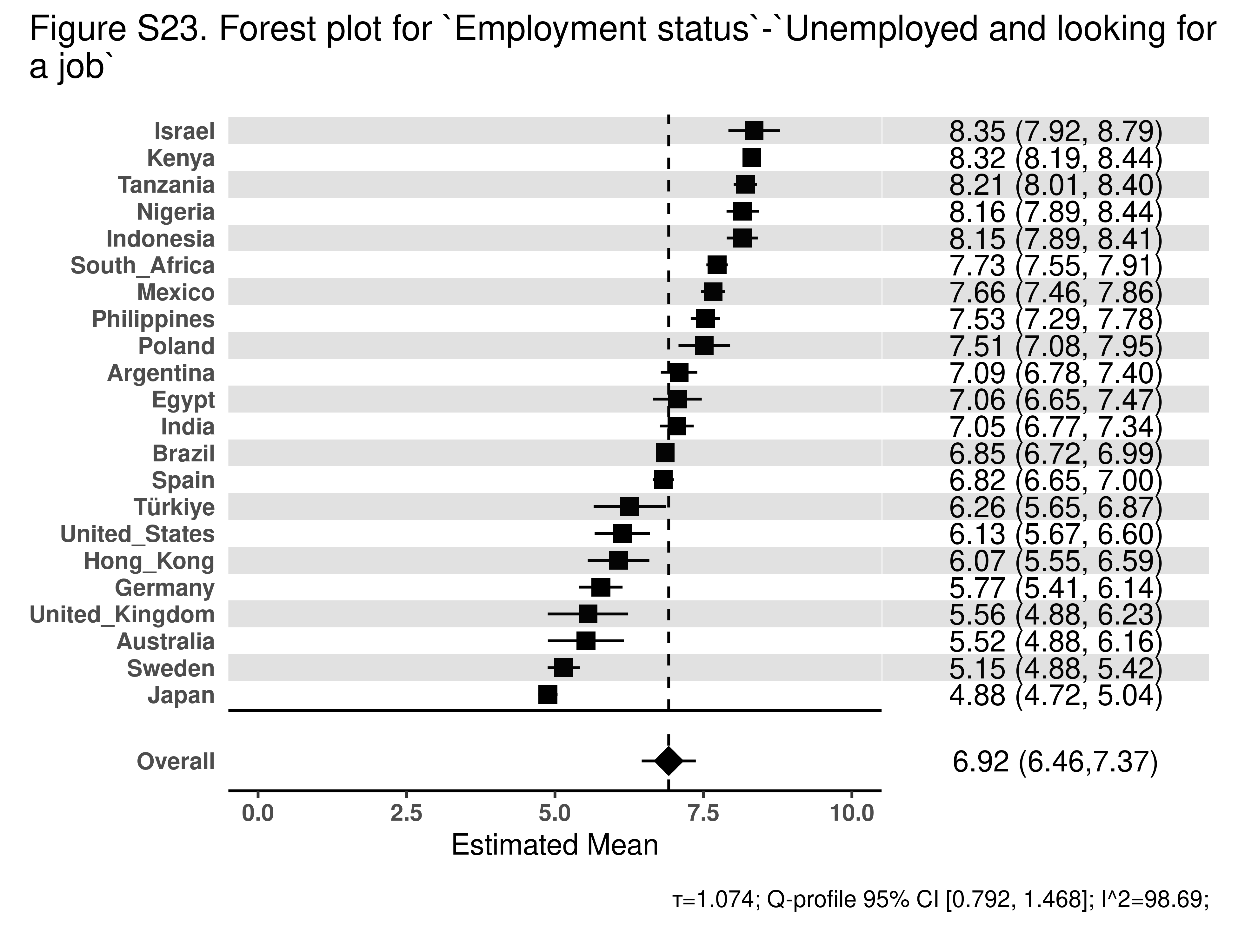


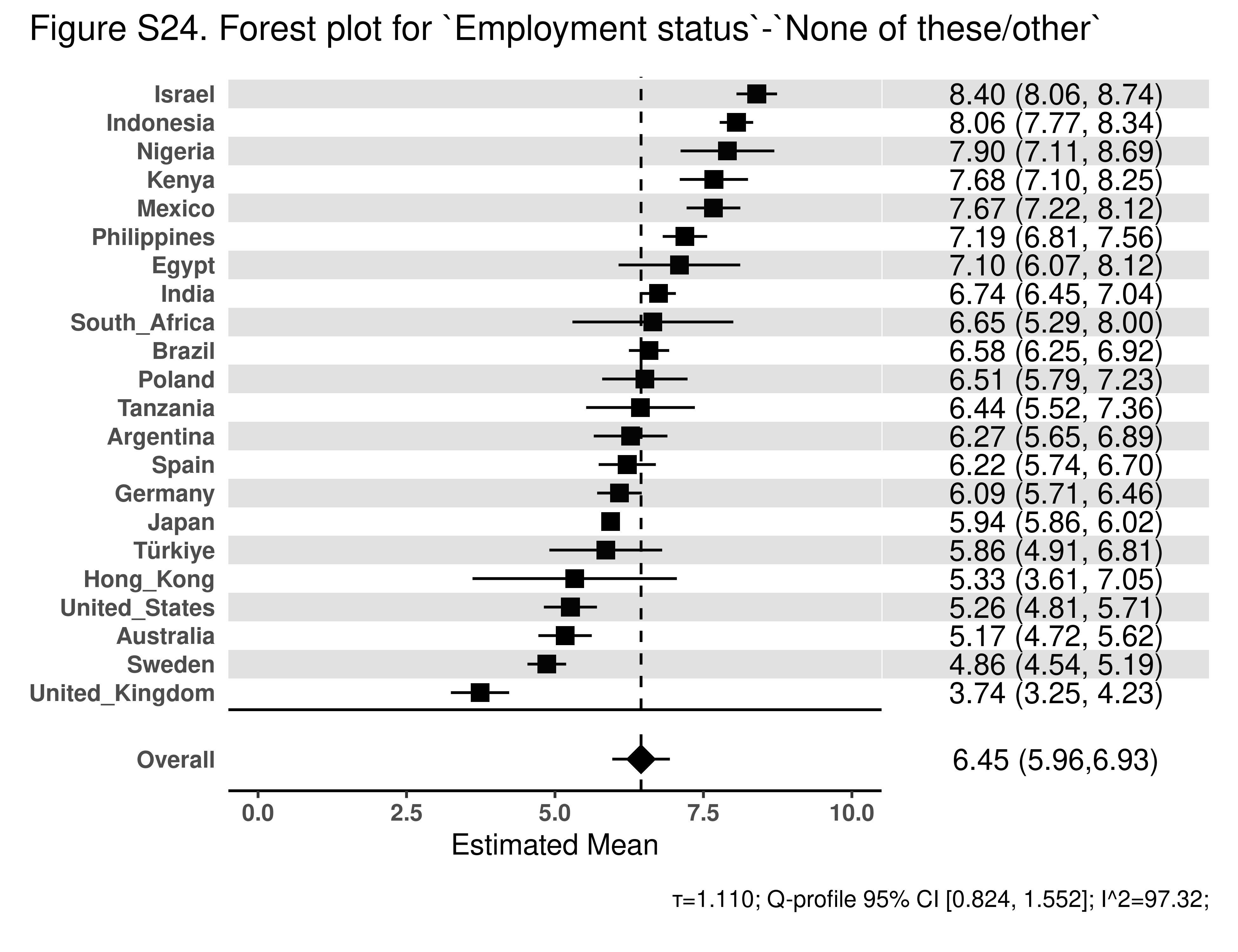


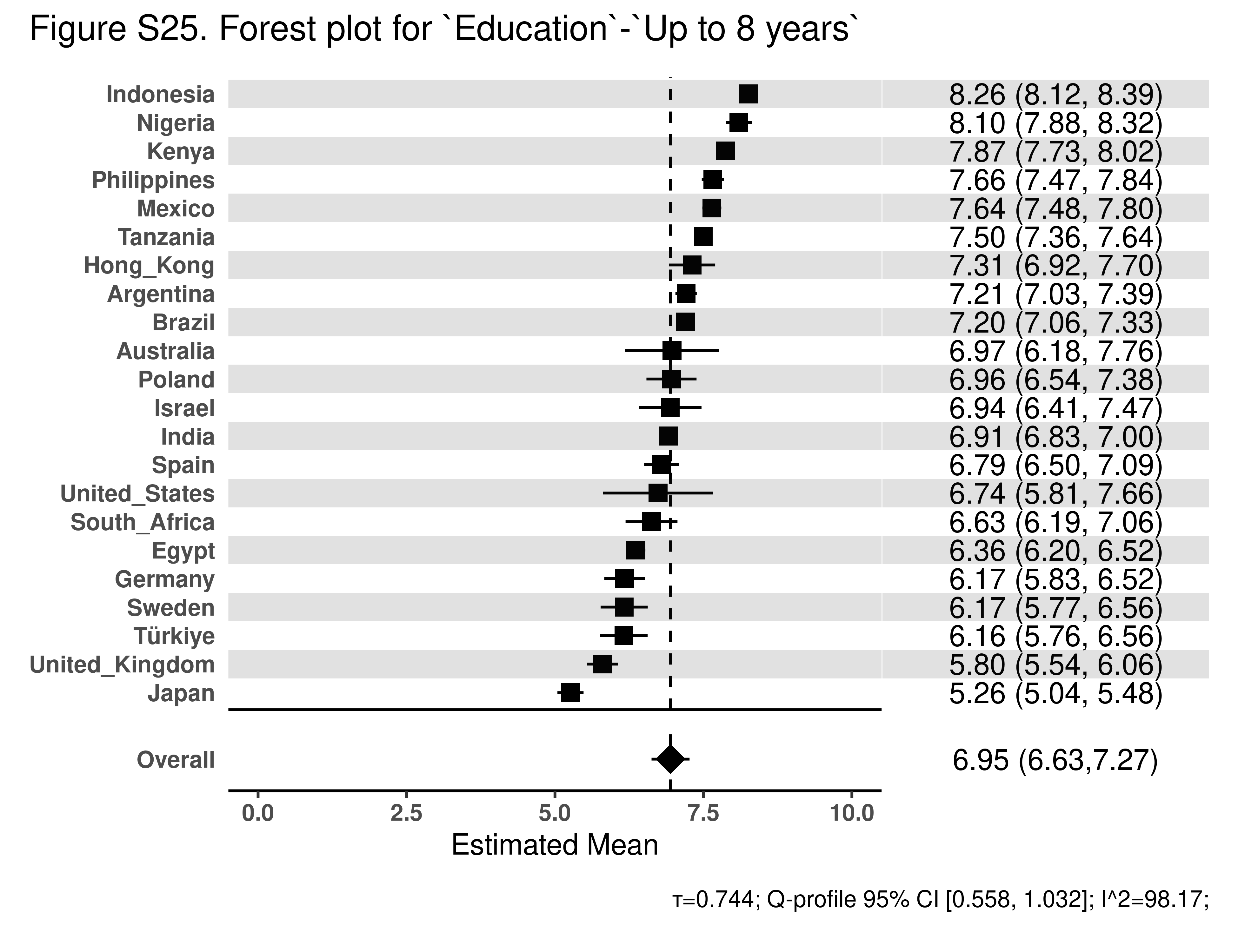


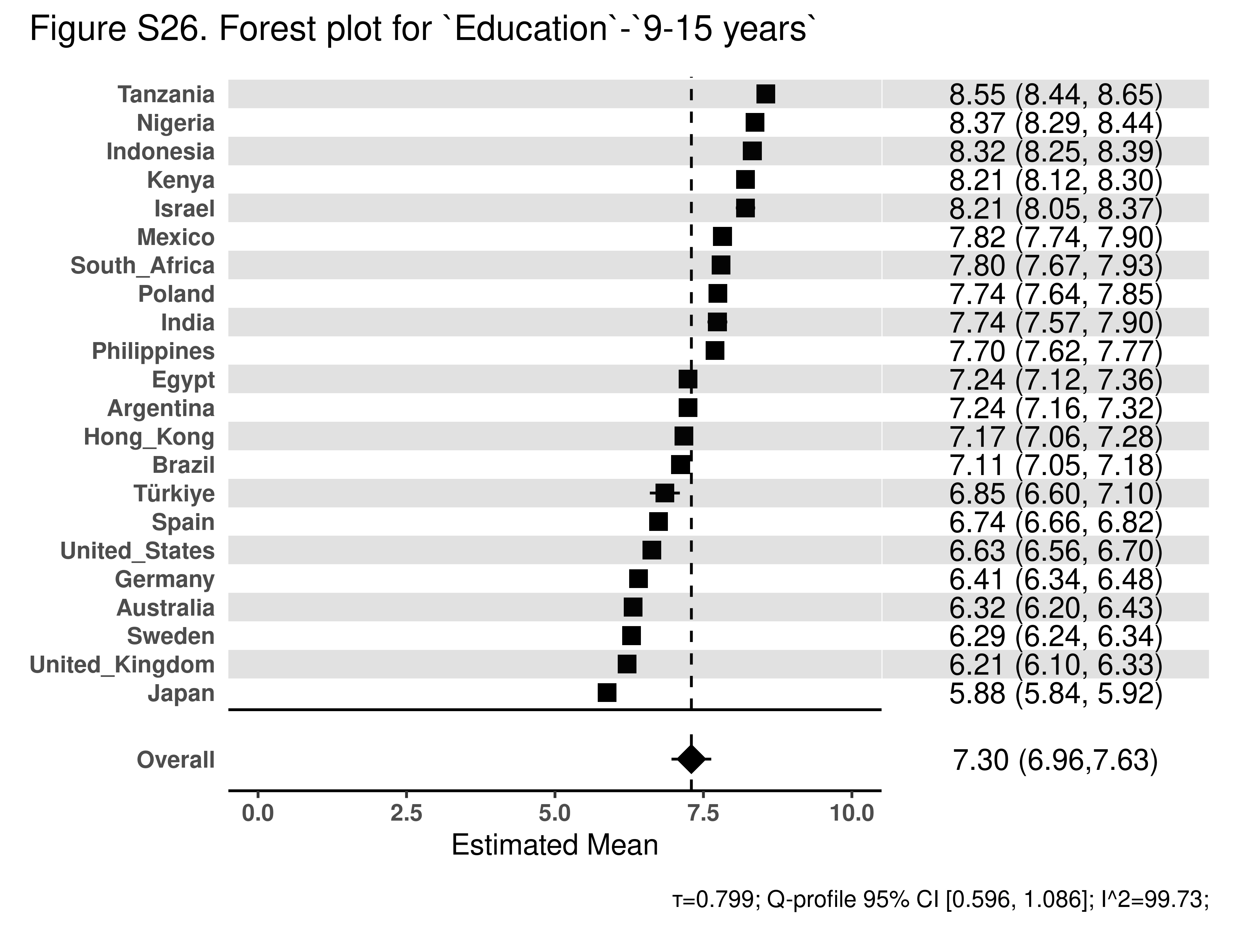


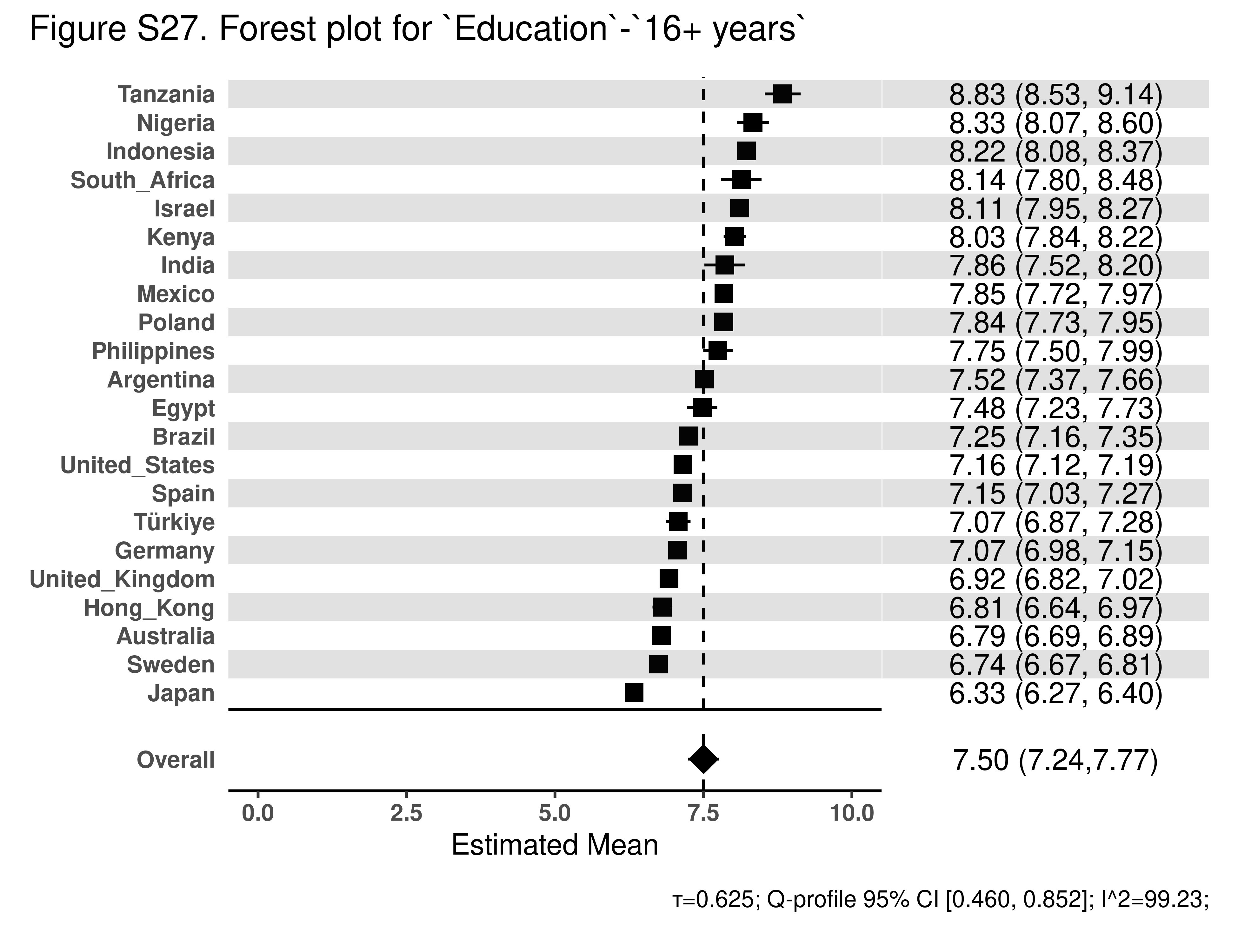


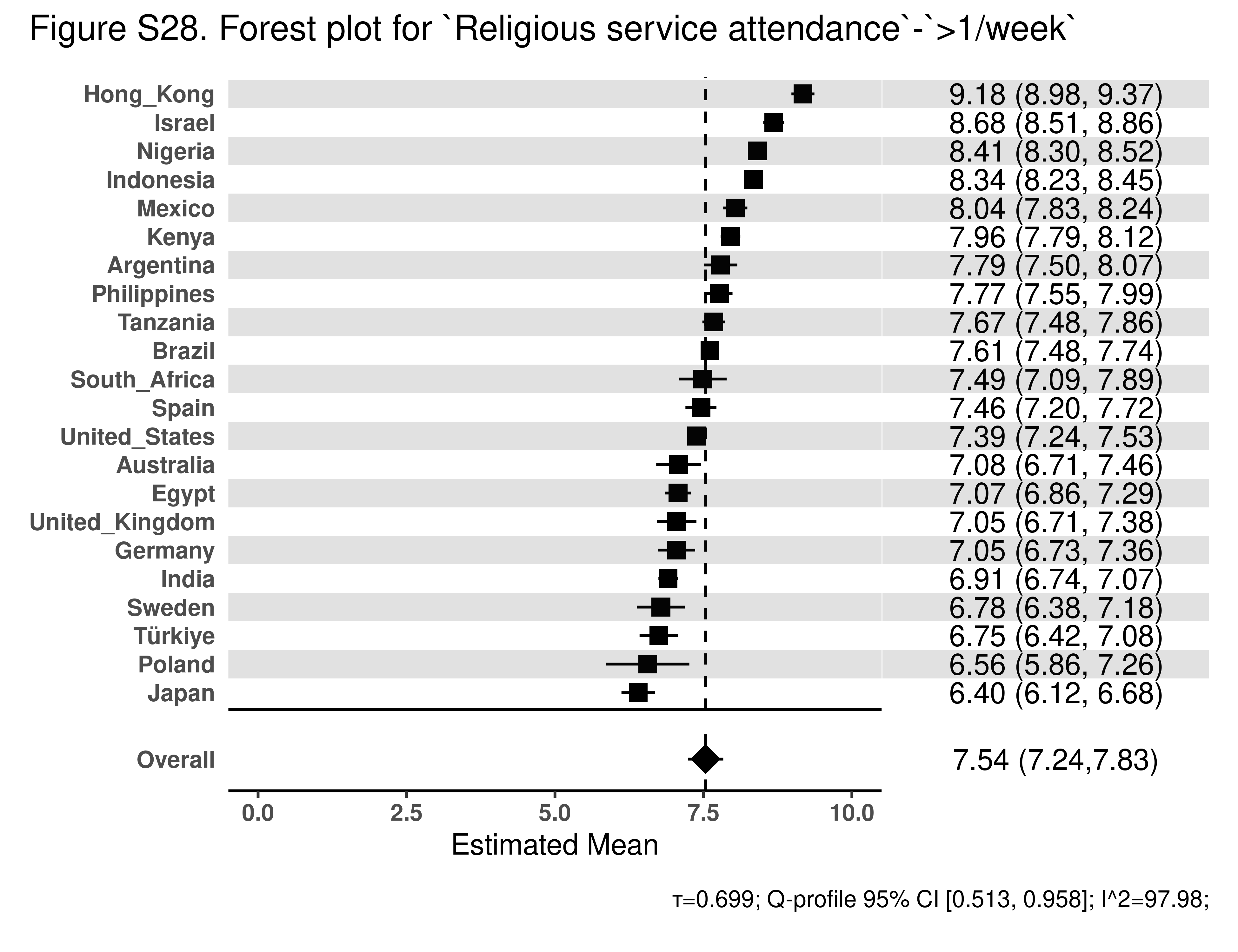


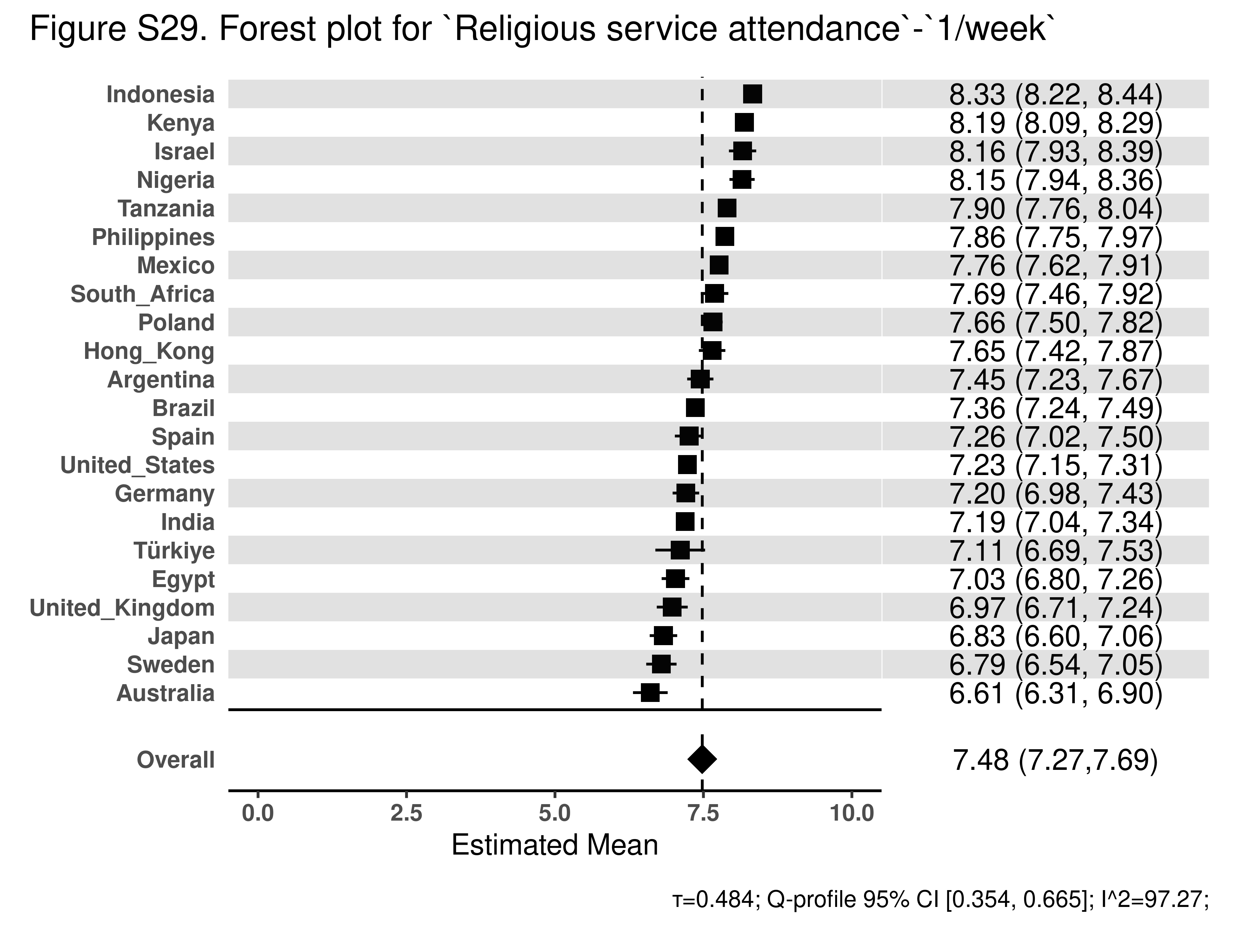


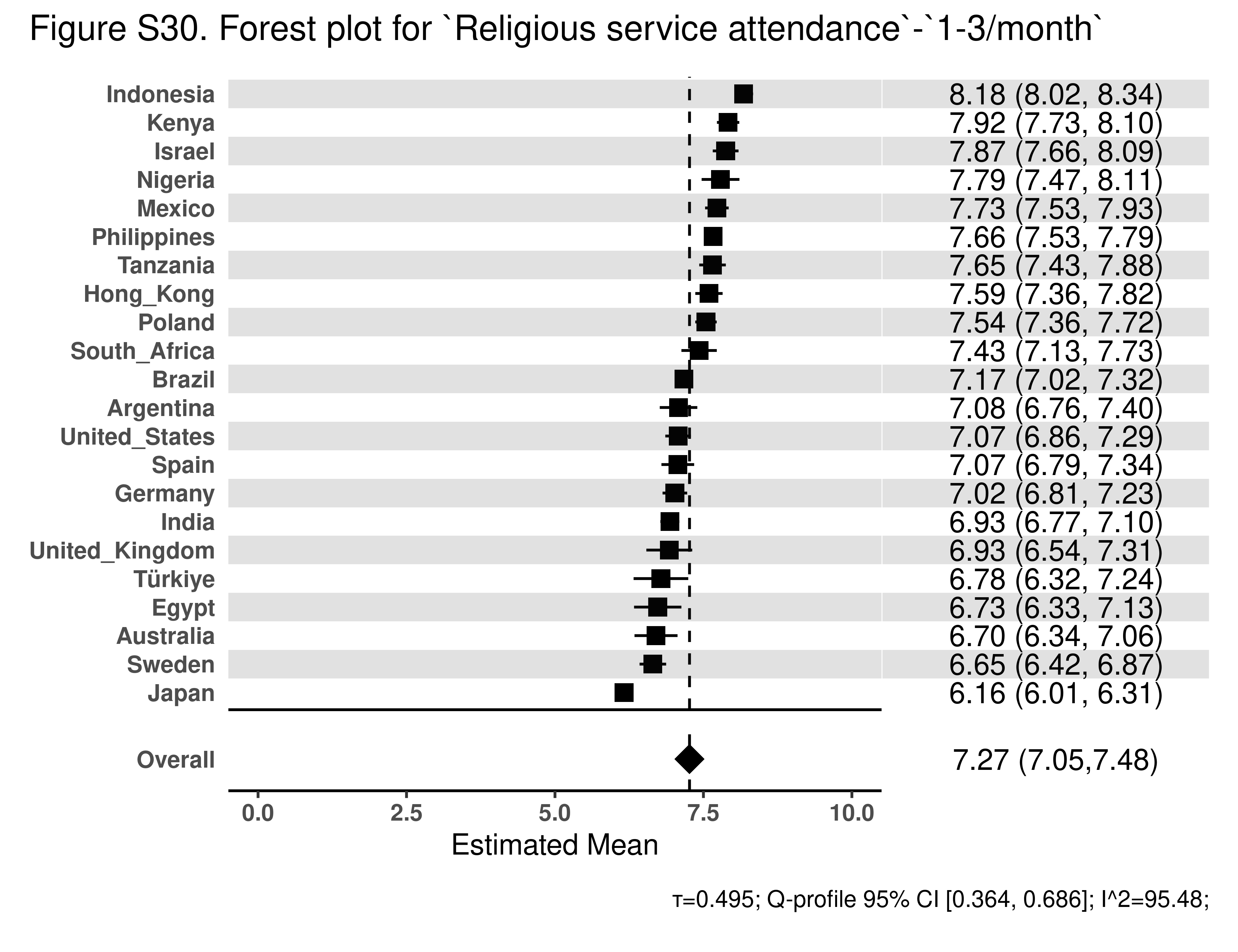


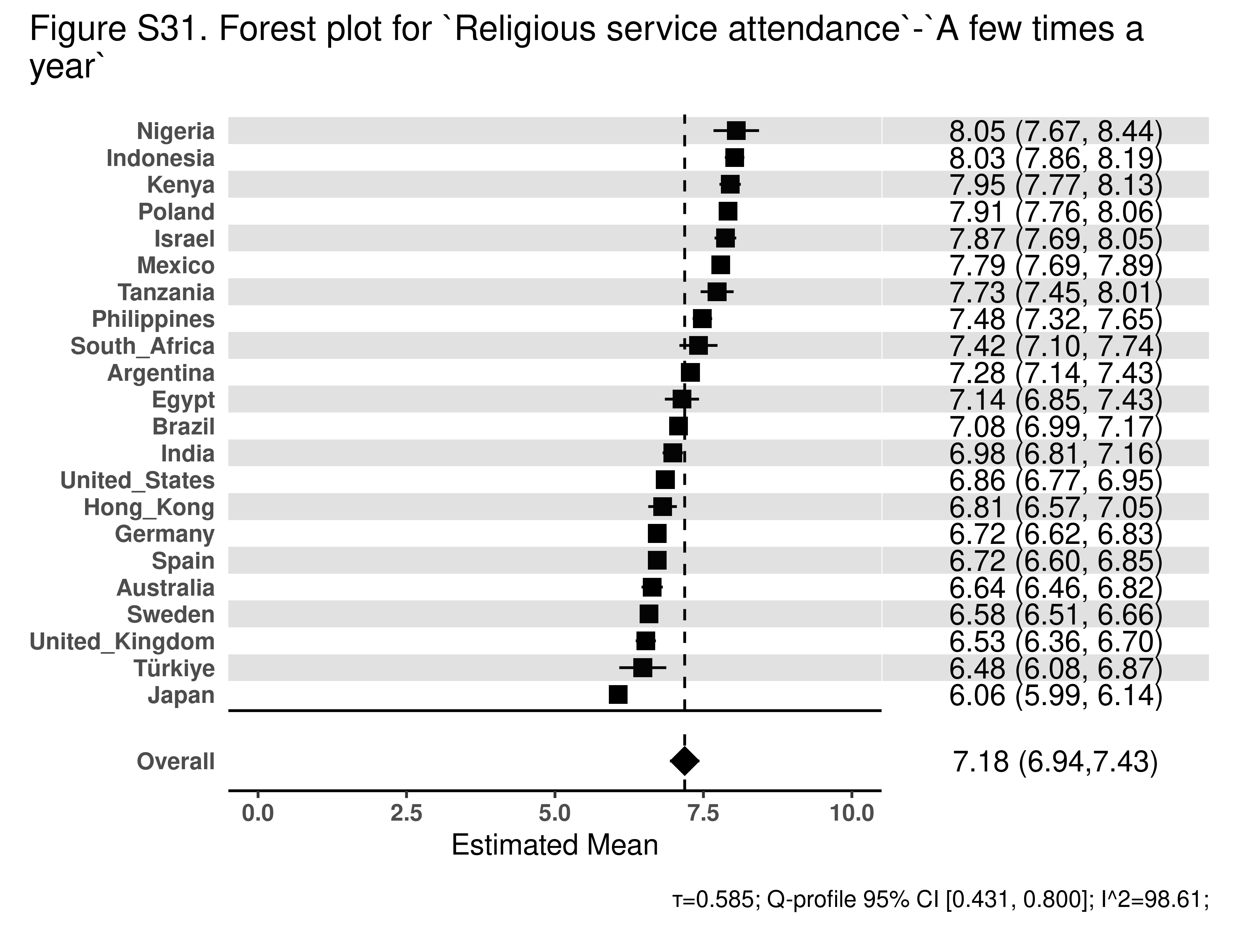


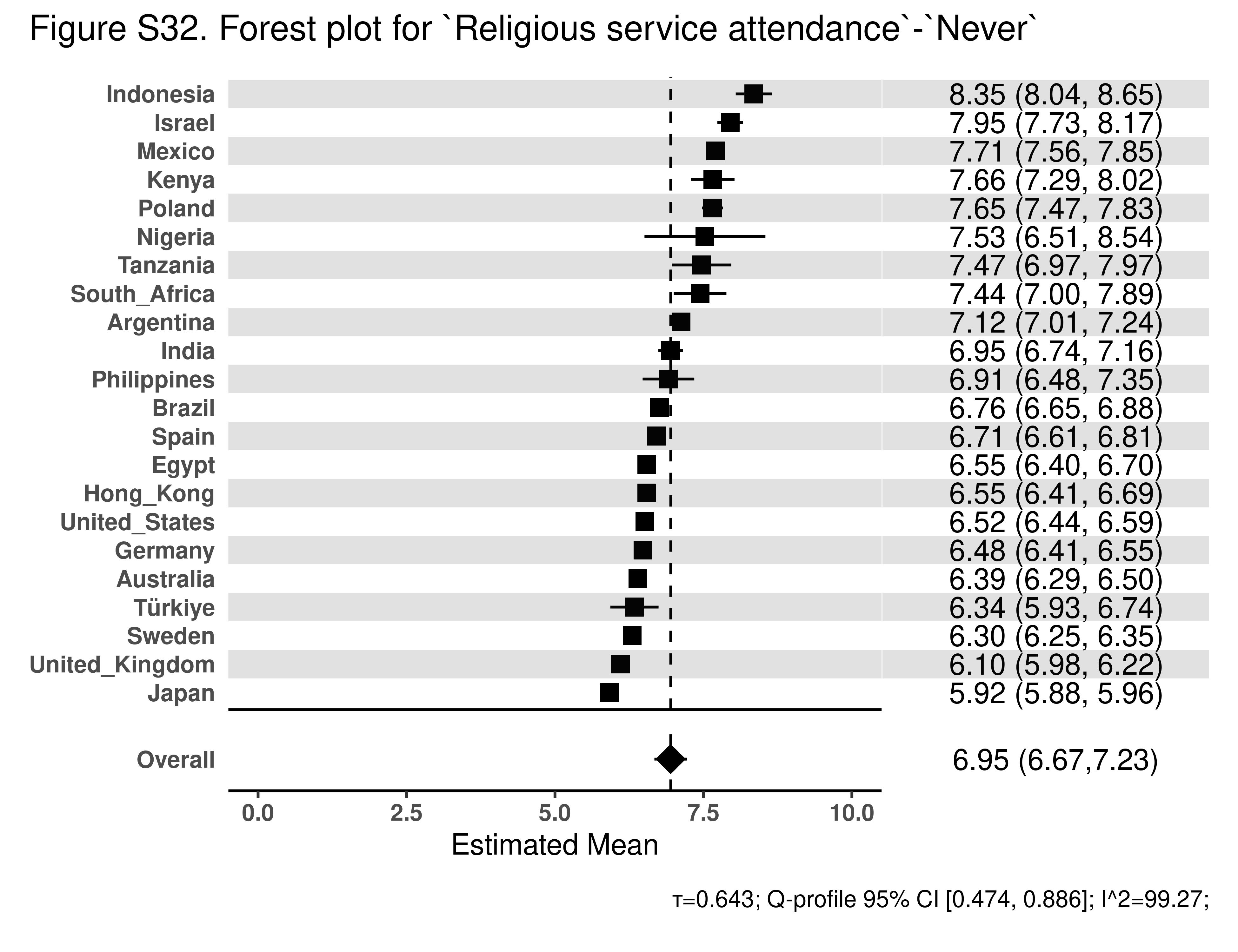


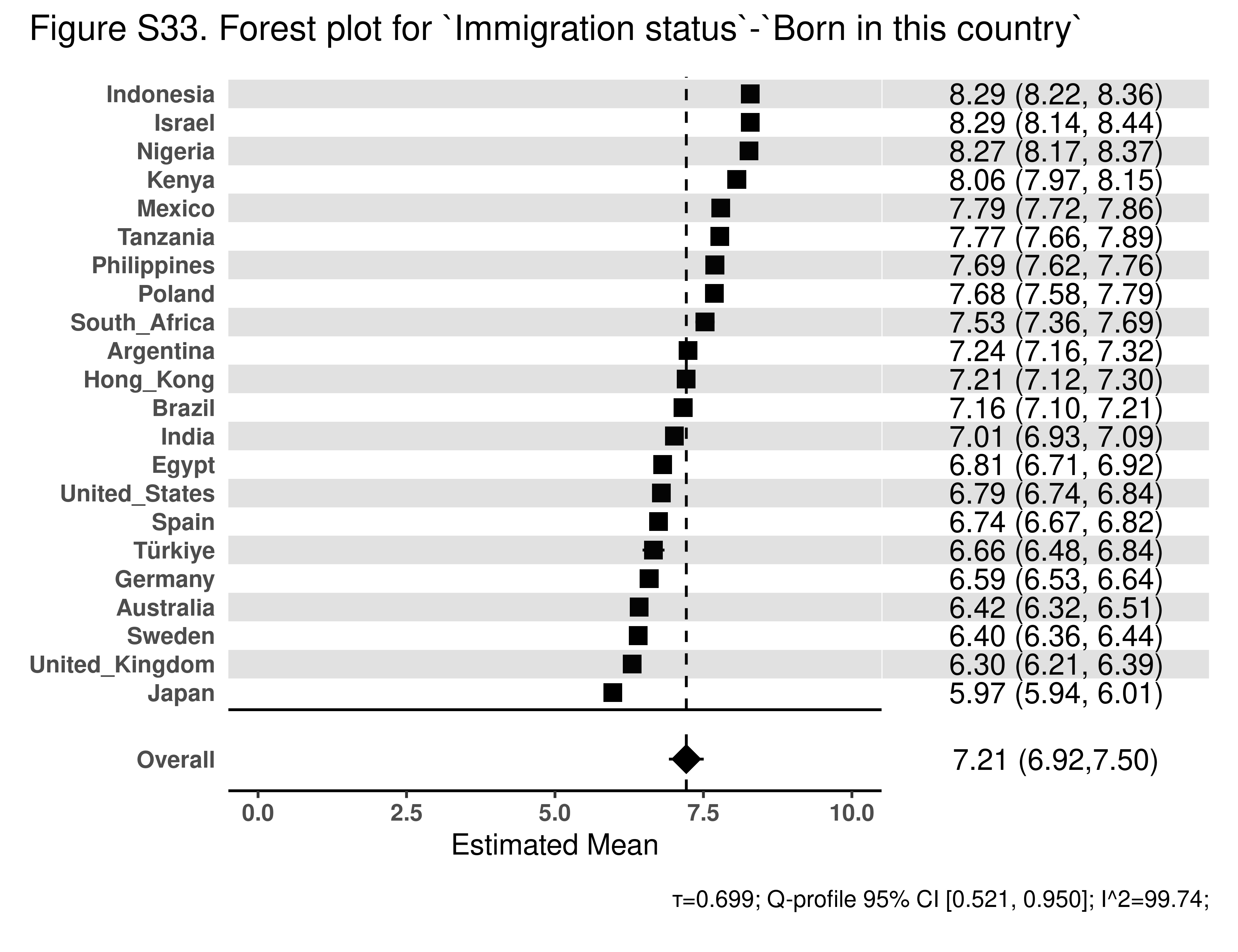


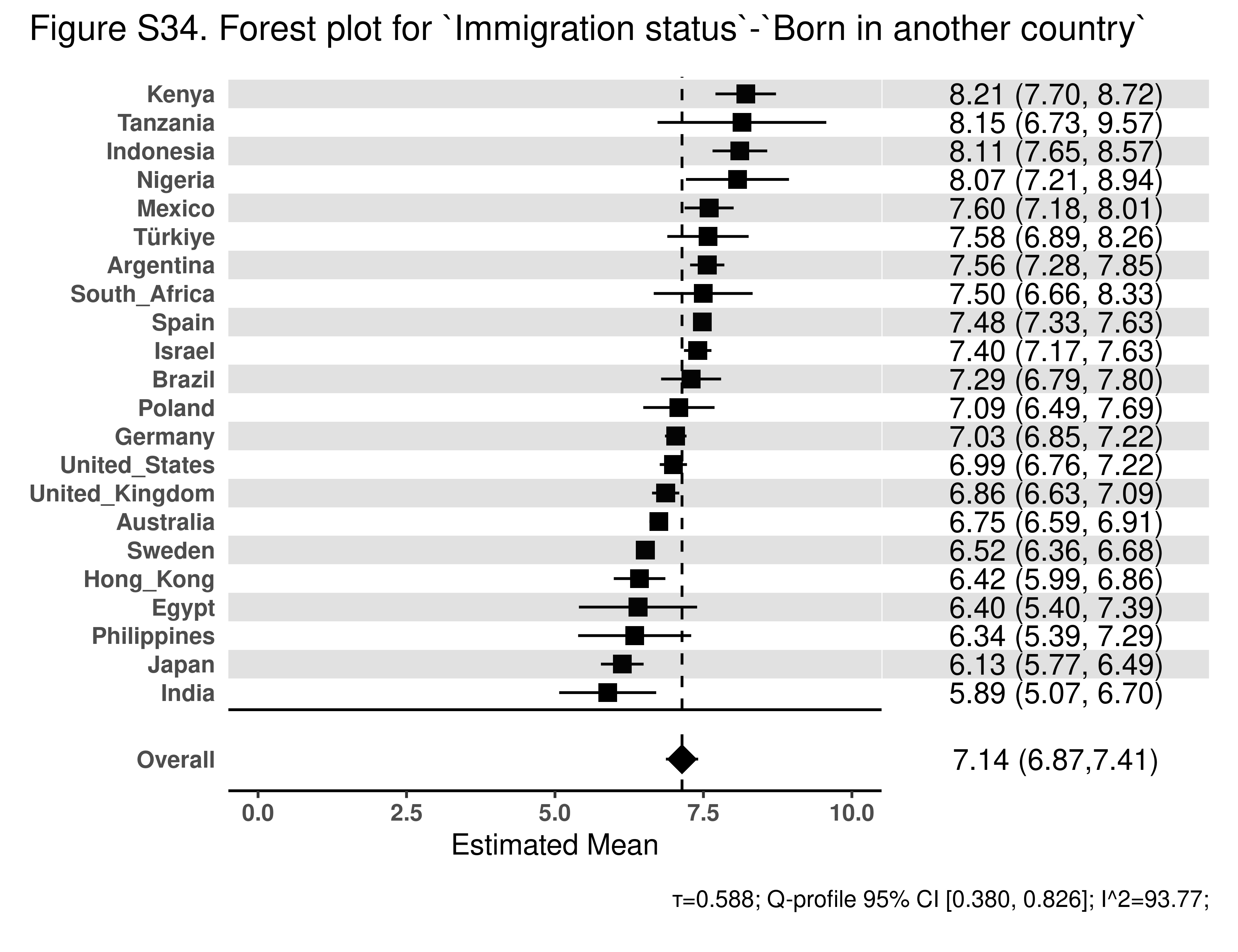

Supplement: Supplementary file 1 — Additional file 1: Demographic Variation in Self-Rated Physical Health Across 22 Countries: Findings from the Global Flourishing Study. Table S1a: Nationally-Representative Descriptive Statistics of the Observed Sample (Argentina). Table S1b: Variations Across Demographic Characteristics (Argentina). Table S2a: Nationally-Representative Descriptive Statistics of the Observed Sample (Australia). Table S2b: Variations Across Demographic Characteristics (Australia). Table S3a: Nationally-Representative Descriptive Statistics of the Observed Sample (Brazil). Table S3b: Variations Across Demographic Characteristics (Brazil). Table S4a: Nationally-Representative Descriptive Statistics of the Observed Sample (Egypt). Table S4b: Variations Across Demographic Characteristics (Egypt). Table S5a: Nationally-Representative Descriptive Statistics of the Observed Sample (Germany). Table S5b: Variations Across Demographic Characteristics (Germany). Table S6a: Nationally-Representative Descriptive Statistics of the Observed Sample (Hong Kong). Table S6b: Variations Across Demographic Characteristics (Hong Kong). Table S7a: Nationally-Representative Descriptive Statistics of the Observed Sample (India). Table S7b: Variations Across Demographic Characteristics (India). Table S8a: Nationally-Representative Descriptive Statistics of the Observed Sample (Indonesia). Table S8b: Variations Across Demographic Characteristics (Indonesia). Table S9a: Nationally-Representative Descriptive Statistics of the Observed Sample (Israel). Table S9b: Variations Across Demographic Characteristics (Israel). Table S9b: Variations Across Demographic Characteristics (Israel). Table S10b: Variations Across Demographic Characteristics (Japan). Table S11a: Nationally-Representative Descriptive Statistics of the Observed Sample (Kenya). Table S11b: Variations Across Demographic Characteristics (Kenya). Table S12a: Nationally-Representative Descriptive Statistics of the Observed Sample (Mexico). Table S12b: Va [file 44263_2025_141_MOESM1_ESM.docx]
